# Design, synthesis, and apoptotic antiproliferative action of new benzimidazole/1,2,3-triazole hybrids as EGFR inhibitors

**DOI:** 10.3389/fchem.2024.1541846

**Published:** 2025-01-13

**Authors:** Alshimaa A. Y. Ahmed, Anber F. Mohammed, Zainab M. Almarhoon, Stefan Bräse, Bahaa G. M. Youssif

**Affiliations:** ^1^ Pharmaceutical Organic Chemistry Department, Faculty of Pharmacy, Assiut University, Assiut, Egypt; ^2^ Department of Chemistry, College of Science, King Saud University, Riyadh, Saudi Arabia; ^3^ Institute of Biological and Chemical Systems, Institute of Biological and Chemical Systems—Functional Molecular Systems, Karlsruhe Institute of Technology, Karlsruhe, Germany

**Keywords:** benzimidazole, triazole, EGFR, apoptosis, caspases

## Abstract

**Introduction:**

This work outlines the design, synthesis, and biological evaluation of a new series of benzimidazole/1,2,3-triazole hybrids as apoptotic antiproliferative agents that inhibit the EGFR pathway.

**Methods:**

The research assesses the antiproliferative efficacy of compounds **6a-i** and **10a-i** against various cancer cell lines.

**Results and Discussion:**

The research emphasizing hybrids **6i** and **10e** for their remarkable activity, with GI_50_ values of 29 nM and 25 nM, respectively. The inhibitory effects of the most potent hybrids **6e**, **6i**, **10d**, **10e**, and **10g** on EGFR were assessed. Compounds **6i** and **10e** exhibited greater potency than erlotinib as EGFR inhibitors. Compounds **6i** and **10e** were also examined for their apoptotic potential, revealing that these compounds promote apoptosis by activating caspase-3, caspase-8, and Bax, while down-regulating the anti-apoptotic protein Bcl-2. Molecular docking experiments are thoroughly examined to validate the binding interactions of the most active hybrids, **6i** and **10e**, with the EGFR active site. Furthermore, our new study examined the ADME properties of the new hybrids.

## 1 Introduction

Cancer remains one of the most severe diseases globally. The World Health Organization (WHO) states that cancer is the second leading cause of death globally (Workie et al., 2023). By 2030, 21.6 million new cancer cases will be annually (Gona et al., 2024). The epidermal growth factor receptor (EGFR) is a member of the ErbB family of receptor tyrosine kinases. Various malignancies, such as breast cancer and head and neck squamous cell carcinoma, increase its expression. Furthermore, EGFR overexpression occurs in approximately fifty percent of patients with non-small cell lung cancer (NSCLC) (Palumbo et al., 2023; Hatil et al., 2020). Consequently, EGFR represents a compelling target for anticancer treatment, leading to the development of many EGFR tyrosine kinase inhibitors (TKIs) (Metibemu et al., 2019; Abourehab et al., 2021; Levantini et al., 2022). They function by competitive inhibition of adenosine triphosphate (ATP) binding in the tyrosine kinase domain.

Benzimidazole is well-known in pharmacology, as its derivatives are often linked to diverse biological activities. The scaffold is a structural isostere of indole and purine; hence, its derivatives are anticipated to have a favorable affinity with diverse receptor types (Nardi et al., 2023; Acar Çevik et al., 2024). Benzimidazole derivatives demonstrate anticancer action by several mechanisms, including the inhibition of topoisomerase I and II, DNA intercalation, PARP-poly inhibition, and the inhibition of dihydrofolate reductase (DHFR) and aromatase (Çevik et al., 2022; Karadayi et al., 2020; Mostafa et al., 2019). For example, derivatives are used in Veliparib and Nocodazole, two well-known cancer medications that inhibit poly (ADP ribose) polymerase (PARP) and disrupt microtubule function, respectively (Yanaihara et al., 2023). Benzimidazole interacts with Met769 of EGFR in a binding manner similar to quinazoline, with the nitrogen atoms in the nucleus acting as hydrogen bond acceptors (Abdullah et al., 2022). Because benzimidazole is structurally similar to quinazoline, which is the building block for first- and second-generation drugs, this chemical could be a good starting point for future EGFR antagonists (Peerzada et al., 2023). However, more studies are needed before the benzimidazole-based drug is clinically approved for EGFR inhibition applications. Most research that examined the structures of benzimidazole derivatives produced conflicting results on the type and placement of substituents on the primary structure, as well as how well adding electron-withdrawing and electron-donating groups increased the capacity to bind to EGFR (JALIL and ABD HAMID, 2023).

**FIGURE 1:**
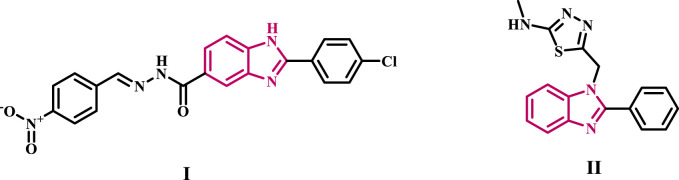
Structure of some benzimidazole-based EGFR inhibitors **I** and **II**.

We recently (Youssif et al., 2024) disclosed compound **I** (Figure 1), a benzimidazole-based anticancer agent selected by NCI for five-dose evaluation against 60 human carcinoma cell lines. Compound **I** exhibited significant selectivity towards the leukemia subpanel, with a selectivity ratio 5.96 at the GI_50_ level. Compound **I** was evaluated for its inhibitory effect on EGFR as a possible target for antiproliferative activity. The findings indicated that **I** exhibited a substantial antiproliferative effect as an EGFR inhibitor. Furthermore, compound **I** triggered apoptosis by elevating caspase-3, caspase-8, and Bax levels while reducing the anti-apoptotic protein Bcl2.

Celik et al. (2019) identified compound **II**, a benzimidazole derivative, as a strong inhibitor of EGFR with antiproliferative properties. The docking investigation revealed that compound **II** had two hydrogen bonding interactions with the residues Lys721 and Thr830 within the binding pocket of EGFR.

On the other hand, 1,2,3-triazoles are nitrogen-containing heterocycles with three nitrogen atoms per ring. 1,2,3-Triazoles are stable molecules that form hydrogen bonds with biological targets. This makes them important building blocks for finding new drugs (Mahmoud et al., 2024; Maghraby et al., 2023). 1,2,3-Triazole compounds have diverse pharmacological properties, with anticancer action being the most prominent (Mahmoud et al., 2023). Researchers have documented the anticancer properties of 1,2,3-triazoles through various mechanisms. 1,2,3-Triazoles inhibit enzymes implicated in the advancement of this lethal disease, such as carbonic anhydrases (CAs) (Fatima et al., 2024), aromatase (Mishra and Upadhyay, 2022), vascular endothelial growth factor receptor (VEGFR) (Nguyen et al., 2022), and epidermal growth factor receptor (EGFR) (Kumar et al., 2023).

**FIGURE 2:**
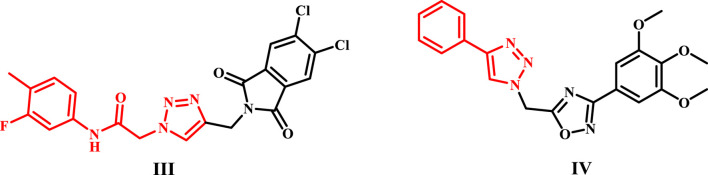
Structure of some 1,2,3-triazole-based EGFR inhibitors **III** and **IV**.

The phthalimide scaffold hybridized with the 1,2,3-triazole moiety (**III**, Figure 2), with an IC_50_ value of 0.22 μM, was particularly effective as an antiproliferative agent against MCF-7 cells. It also demonstrated strong EGFR inhibition, with an IC_50_ value of 79 nM, slightly higher than that of erlotinib. Compound **III** caused MCF-7 cells to undergo apoptosis, cell cycle arrest in the S/pre-G1 stages, and DNA fragmentation. The docking of **III** revealed hydrogen bonding interactions between the nitrogen of the 1,2,3-triazole ring and the Met769 residue, identical to the reference medication erlotinib. This demonstrates the role of the 1,2,3-triazole fragment in blocking the EGFR for anticancer therapy (Ihmaid et al., 2021).

We recently (Mahmoud et al., 2024) reported on the design and synthesis of a novel class of 1,2,3-triazole/1,2,4-oxadiazole hybrids that act as dual inhibitors of EGFR/VEGFR-2. The newly synthesized compounds were tested as antiproliferative agents using erlotinib as the reference medication. The results showed that most of the compounds tested had strong antiproliferative effects, with GI_50_ values ranging from 28 to 104 nM, whereas erlotinib’s GI_50_ value was 33 nM. The finding’s showed compound **IV** was the best derivative as an EGFR inhibitor, with an IC_50_ value of 76 nM, which is lower (more potent) than the reference drug erlotinib’s value of 80 nM. The docking analysis of **IV** within the EGFR active site demonstrated that the phenyl triazole moiety was deeply embedded in the hydrophobic pocket, corresponding with the phenylacetylene moiety of erlotinib. Furthermore, the 1,2,3-triazole molecule establishes a hydrogen connection with the Lys721 amino acid residue.

**FIGURE 3:**
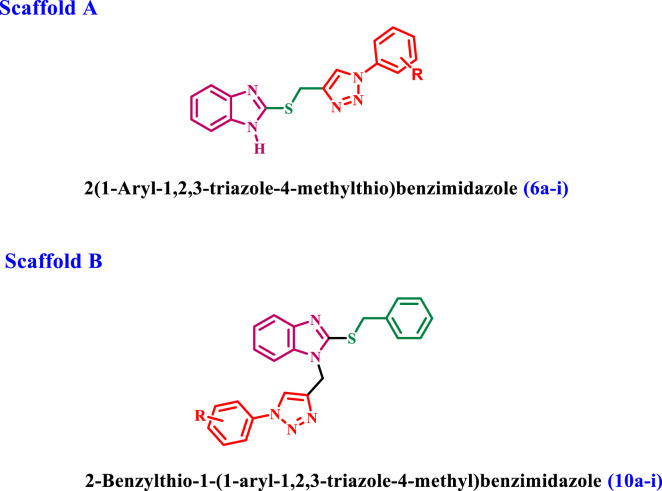
Structure of new benzimidazole/1,2,3-triazole hybrids **6a-i** and **10a-i**.

In our continuous pursuit of anticancer drugs targeting EGFR (El-Sherief et al., 2018; Abou-Zied et al., 2019; Mahmoud et al., 2022; Al-Wahaibi et al., 2023; Mostafa et al., 2024), we synthesized and evaluated a series of benzimidazole/1,2,3-triazole hybrids (**6a-i** and **10a-i**, Figure 3) for their efficacy against EGFR. The recently synthesized compounds are classified into two categories (Figure 3): compounds of scaffold A are 2 (1-aryl-1,2,3-triazole-4-methylthio)benzimidazoles **6a-i**, while scaffold B comprises 2-benzylthio-1-(1-aryl-1,2,3-triazole-4-methyl)benzimidazoles **10a-i**. The newly synthesized compounds were tested *in vitro* against a panel of four cancer cell lines as antiproliferative agents. The most effective compounds were subsequently evaluated for EGFR inhibitory activity. Furthermore, the apoptotic efficacy of the most potent derivatives was assessed. Ultimately, docking analysis and ADMET evaluations were performed to determine the most effective variants.

## 2 Results and discussion

### 2.1 Chemistry

**SCHEME 1:**
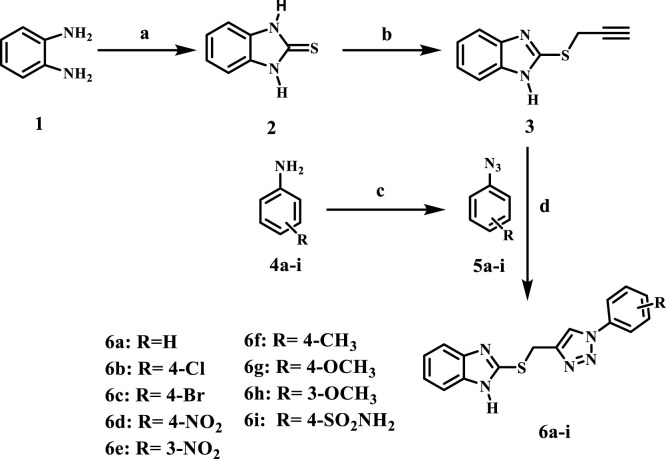
Synthesis of the new compounds **6a-i**. Reagents and reaction conditions: **(A)** CS_2_, KOH, EtOH: H_2_O (1.5:1), reflux, 30 h, yield, 90% **(B)** propargyl bromide, K_2_CO_3_, dry acetone, 7 h, yield, 70% **(C)** NaNO_2_, NaN_3_, HCl, H_2_O, stirring, 1 h, yield, 53% **(D)** CuSO_4,_ Na ascorbate, THF: H_2_O (1:1), stirring, 24 h, yield, 50%–60%.

Scheme 1 outlines the synthetic routes of the key intermediates and novel compounds **6a-i**. Benzimidazole-2-thione **(2)** was synthesized through the reaction of *o*-phenylenediamine **(1)** with carbon disulfide in the presence of potassium hydroxide in an ethanol/water mixture in 82% yield (Latif et al., 2021). Compound **3**, 2-(prop-2-yn-1-ylthio)-1*H*-benzimidazole was synthesized via the alkylation of compound **2** using propargyl bromide in the presence of anhydrous potassium carbonate in dry acetone (Dunga et al., 2022). Furthermore, we synthesized the substituted azide derivatives **5a-i** from aryl amines through arene-diazonium salts using a documented method (Kutonova et al., 2013).

The final target compounds **6a-i** was prepared through the click reaction of 2-propargayl-thiobenzimidazole **3** with the appropriate azides **5a-i** in the presence of CuSO_4_ and sodium ascorbate with a THF: H_2_O (1:1) mixture, Scheme 1. ^1^H NMR, ^13^C NMR, elemental microanalyses, and representative IR spectral analysis established the structure elucidation of the new compounds. In general, the ^1^H NMR spectra of **6a-i** verified the appearance of a broad singlet signal at 12.62–13.04 ppm corresponding to benzimidazole NH. In addition, two singlet signals appeared at 8.63–8.99 ppm and 4.69–4.81 ppm for the triazole-CH and SCH_2_ groups, respectively. Also, ^13^C NMR spectra of **6a-i** showed signals at 25.8–27.4 ppm for the SCH_2_ group, and the aromatic carbon signals at δ = 110.9–140 ppm correspond to the benzimidazole carbons. The spectra also revealed the existence of additional signals in the aromatic region for the introduced phenyl moiety. As a representative example, the IR spectrum of compound **6d** displayed a broad band at 3,400 cm^-1^ for the benzimidazole NH as well as two stretching bands at 1,340 cm^-1^ and 1,523 cm^-1^ for the NO_2_ group, along with a bending band at 854 cm^-1^ that confirms the *para*-disubstituted pattern.

**SCHEME 2:**
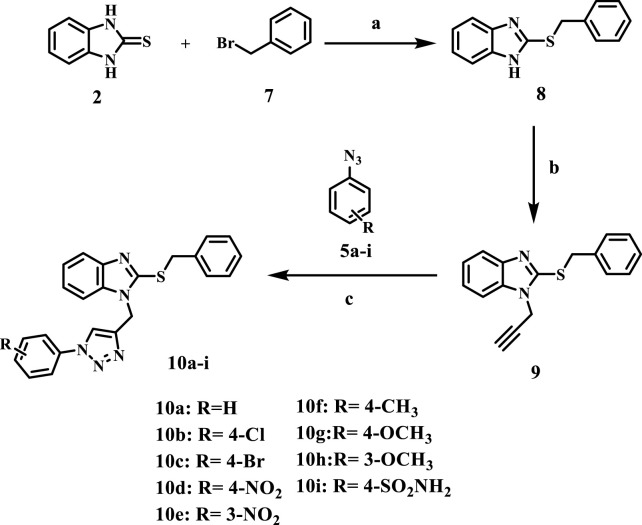
Synthesis of the new compounds (**10a-i**). Reagents and reaction conditions: **(A)** benzyl bromide, K_2_CO_3_, dry acetone, 15 h, yield, 72% **(B)** propargyl bromide, K_2_CO_3_, dry acetone, 10 h, yield, 71% **(C)** CuSO_4_, Na ascorbate, THF: H_2_O (1:1), stirring, 24 h, yield, 30%–40%.

Scheme 2 outlines the synthetic routes of the key intermediates and novel compounds 10a-**i**. Compound **8**, 2-benzylthiobenzimidazole, was synthesized via the alkylation of compound **2** using benzyl bromide in the presence of anhydrous potassium carbonate in dry acetone (Dunga et al., 2022). Subsequently, 2-benzylthiobenzimidazole **8** was alkylated with propargyl bromide at the benzimidazole NH according to the compound **3** method of synthesis using K_2_CO_3_ and acetone to afford compound 9 (Kalyani and Manikyamba, 2004), Scheme 2.

Like **6a-i**, compounds **10a-i** were synthesized by a click reaction between **9** and the appropriate azide derivatives **5a-i**. Their structures were elucidated using IR, NMR, and elemental microanalyses. The ^1^H NMR spectra of **10a-i** generally confirmed the appearance of two singlet signals at 4.72–4.79 ppm, 5.47–5.49 ppm, and 8.76–8.90 ppm for the SCH_2_, NCH_2,_ and triazole CH groups, respectively. The spectra also revealed the existence of additional signals in the aromatic region for the introduced phenyl moiety along with the aromatic protons of the benzimidazole ring and the 2-thiobenzyl ring. In addition, ^13^C NMR spectra of **10a-i** displayed signals at 36.57–36.61 ppm and 39.11–39.34 ppm for the SCH_2_ and NCH_2_ groups, respectively. Benzimidazole carbon signals appear in the δ = 110.5–137.8 ppm range. As a representative example, the IR spectrum of compound **10d** displayed two stretching bands at 1,340 cm^-1^ and 1,523 cm^-1^ for the NO_2_ group, along with a bending band at 854 cm^-1^ that confirms the *para*-disubstituted pattern.

### 2.2 Biology

#### 2.2.1 Cell viability assay

The MCF-10A normal human mammary gland epithelial cell line was used to test the effects of compounds **6a-i** and **10a-i** on cell viability. The MTT assay was used to check the cell viability effect of compounds **6a-i** and **10a-i** after 4 days of treatment with MCF-10A cells (Mekheimer et al., 2022; Hisham et al., 2022). Table 1 results indicate that none of the examined compounds exhibited cytotoxicity, as all hybrids maintained cell viability above 84% at a concentration of 50 µM.

**TABLE 1:** IC_50_ values of compounds **6a-i** and **10a-i** against four cancer cell lines.

Comp	Cell viability %	Antiproliferative activity IC50 ± SEM (nM)					
		R	A-549	MCF-7	Panc-1	HT-29	Average IC50 (GI50)
**6a**	84	H	89 ± 8	85 ± 7	89 ± 8	88 ± 8	88
**6b**	87	Cl	47 ± 4	45 ± 4	48 ± 4	48 ± 4	47
**6c**	91	Br	96 ± 9	90 ± 8	96 ± 9	94 ± 9	94
**6d**	93	4-NO2	85 ± 7	83 ± 7	86 ± 8	86 ± 8	85
**6e**	86	3-NO2	40 ± 3	36 ± 3	38 ± 3	38 ± 3	38
**6f**	90	4-Me	81 ± 7	78 ± 7	80 ± 7	82 ± 7	80
**6g**	89	4-OMe	72 ± 7	69 ± 6	72 ± 7	74 ± 7	72
**6h**	91	3-OMe	69 ± 6	66 ± 6	69 ± 6	68 ± 6	68
**6i**	90	4-SO2NH2	30 ± 2	28 ± 2	28 ± 2	30 ± 2	29
**10a**	87	H	59 ± 5	56 ± 5	60 ± 5	60 ± 5	59
**10b**	86	Cl	48 ± 4	44 ± 3	48 ± 4	48 ± 4	46
**10c**	92	Br	55 ± 5	52 ± 5	54 ± 5	54 ± 5	54
**10d**	89	4-NO2	37 ± 3	34 ± 3	36 ± 3	38 ± 3	36
**10e**	90	3-NO2	26 ± 2	24 ± 2	26 ± 2	25 ± 2	25
**10f**	89	4-Me	66 ± 6	63 ± 6	65 ± 6	65 ± 6	65
**10g**	90	4-OMe	34 ± 3	32 ± 3	34 ± 3	35 ± 3	34
**10h**	89	3-OMe	76 ± 7	72 ± 7	76 ± 7	76 ± 7	75
**10i**	91	4-SO2NH2	44 ± 4	42 ± 4	44 ± 4	45 ± 4	43
**Erlotinib**	ND	NA	30 ± 3	40 ± 3	30 ± 3	30 ± 3	33

ND, not determined.

NA, not applicable.

#### 2.2.2 Antiproliferative assay

We assessed the antiproliferative efficacy of novel compounds **6a-i** (Scaffold A) and **10a-i** (Scaffold B) against four human cancer cell lines (colon: HT-29, lung: A-549, breast: MCF-7, and pancreatic: Panc-1) (El-Sherief et al., 2019; Al-Wahaibi et al., 2022). Erlotinib served as the control in this investigation. Table 1 displays the median inhibitory concentration (IC_50_) and GI_50_ (mean IC_50_) values for the four cancer cell lines.

The tested compounds **6a-i** and **10a-i** had strong antiproliferative activity, with GI_50_ values ranging from 25 nM to 94 nM against the four cancer cell lines that were tested. This is in comparison to the standard erlotinib, which had a GI_50_ value of 33 nM. The five most potent derivatives were compounds **6e**, **6i**, **10d**, **10e**, and **10g**, with GI_50_ values ranging from 25 nM to 38 nM. Compounds **6i** and **10e**, with GI_50_ values of 25 and 29 nM, demonstrated greater potency than erlotinib, which had a GI_50_ of 33 nM.

With a GI_50_ value of 25 nM, compound **10e** (R = 3-NO_2_, Scaffold B) was the most effective of the newly synthesized derivatives **6a-i** and **10a-i**. It was 1.4 times stronger than erlotinib (GI_50_ = 33 nM). Compound **10e** demonstrated greater potency than the reference erlotinib against all tested cancer cell lines. Compound **6i** (R = 4-SO_2_NH_2_, Scaffold A) had the second-highest activity, with a GI_50_ value of 29 nM. It was slightly more effective than the standard erlotinib, whose GI_50_ value was 33 nM. Compound **6i** exhibited greater potency than erlotinib against breast (MCF-7) and pancreatic (Panc-1) cancer cell lines.

The findings show that the type and/or the position of substitutions found on the phenyl ring at position one of the 1,2,3-triazole moiety in both scaffold A and B compounds are essential for antiproliferative action. Compound **10d** (R = 4-NO_2_, Scaffold B), possessing an identical backbone to compound **10e** but featuring a nitro group at the 4-position on the phenyl ring, exhibited a GI_50_ of 36 nM (1.5-fold less potent than **10e**), indicating that the nitro group at the 3-position is more conducive to antiproliferative activity than at the 4-position. Unfortunately, this requirement does not apply to all derivatives of scaffold B compounds. Compound **10g** (R = 4-OMe, Scaffold B) demonstrated the third greatest activity, with a GI_50_ of 34 nM. Shifting the methoxy group from position 4 on the phenyl ring to position 3, as in compound **10h** (R = 3-OMe, Scaffold B), resulted in a considerable drop in antiproliferative activity. Compound **10h** had a GI_50_ of 75 nM, two times less potent than the 4-methoxy derivative, compound **10g**.

Compounds **10a** (R = H, Scaffold B), **10b** (R = 4-Cl, Scaffold B), **10c** (R = 4-Br, Scaffold B), **10f** (R = 4-Me, Scaffold B), and **10i** (R = 4-SO_2_NH_2_, Scaffold B) demonstrated GI_50_ values of 59, 46, 54, 65, and 43 nM, respectively. All these compounds exhibited lower potency than **10e** (R = 3-NO_2_, Scaffold B) and even **10d** (R = 4-NO_2_, Scaffold B). These data demonstrate that in scaffold B compounds, the nature and/or position of the substitutions significantly influences activity, with activity increasing in the following order: 3-NO_2_ > 4-OMe > 4-NO_2_ > 4-SO_2_NH_2_ > Cl > Br > H > Me. The same is true for scaffold A compounds: the most active derivatives are those with NO_2_, SO_2_NH_2_, and OMe groups, followed by those with halogen substituents, and the least active are those with methyl substituting or non-substituting. Finally, a future goal for this research is to synthesize and evaluate more triazole and/or benzimidazole moiety derivatives to achieve an accurate SAR.

#### 2.2.3 EGFR inhibitory assay

The most effective antiproliferative derivatives, **6e**, **6i**, **10d**, **10e**, and **10g**, were evaluated for their ability to inhibit EGFR using the EGFR-TK test (Mahmoud et al., 2023; Alshammari et al., 2022). The results are presented in Table 2. Erlotinib served as the reference compound. The assay results align with those of the antiproliferative assay, indicating that compounds **6i** (R = 4-SO_2_NH_2_, Scaffold A) and **10e** (R = 3-NO_2_, Scaffold B), identified as the most potent antiproliferative agents, are the most efficacious derivatives of EGFR inhibitors, exhibiting IC_50_ values of 78 ± 5 and 73 ± 4, respectively. Compounds **6i** and **10e** demonstrated more potency than erlotinib as EGFR inhibitors, with an IC_50_ value of 80 nM. Compounds **6e**, **10d**, and **10g** exhibited substantial inhibition of EGFR, with IC_50_ values of 89, 86, and 82 nM, respectively. These molecules exhibited marginally reduced potency compared to erlotinib. The data suggest that compounds **6e**, **6i**, **10d**, **10e**, and **10g** are extremely effective antiproliferative candidates that may function as EGFR inhibitors.

**TABLE 2:** IC_50_ values of compounds **6e**, **6i**, **10d**, **10e**, **10g**, and erlotinib against EGFR.

Compound	EGFR inhibition IC_50_ ± SEM (nM)
**6e**	89 ± 6
**6i**	78 ± 5
**10d**	86 ± 6
**10e**	73 ± 4
**10g**	82 ± 5
Erlotinib	80 ± 5

#### 2.2.4 Apoptotic markers assays

Apoptosis is an essential cellular process in animal growth, tissue homeostasis, and immune responses. In a healthy body, a vital equilibrium exists between apoptotic and anti-apoptotic mediators during normal physiological processes. Nonetheless, an imbalance may occur in some circumstances, potentially resulting in illnesses (Al-Mahmoudy et al., 2022). Excessive activation or suppression of apoptotic mediators frequently results in this imbalance. Pathological disorders, such as cancer, can disrupt this equilibrium. Compounds **6i** and **10e**, which demonstrated the greatest potency in all laboratory assays, were examined to assess their ability to initiate the apoptosis cascade and display proapoptotic activity.

##### 2.2.4.1 Assays for caspases 3 and 8

Cells experience apoptosis in reaction to specific signaling cues, resulting in significant modifications. Caspases are considered the primary mediators of apoptosis, initiating the process at an early stage (Sahoo et al., 2023). They decompose vital cellular components, including nuclear proteins, such as DNA repair enzymes and structural proteins within the cytoskeleton, essential for optimal cellular function. Caspases can activate DNases, enzymes that damage nuclear DNA (Larsen and Sørensen, 2017). Compounds **6i** and **10e** were assessed as activators of caspase-3/8 in the MCF-7 breast cancer cell line (Abdelbaset et al., 2019). The results of this experiment are presented in Table 3.

**TABLE 3:** Apoptotic capabilities of compounds **6i** and **10e**.

Compd. No.	Caspase-3		Caspase-8		Bax		Bcl-2	
	Conc (Pg/mL)	Fold change	Conc (ng/mL)	Fold change	Conc (Pg/mL)	Fold change	Conc (ng/mL)	Fold reduction
**6i**	590 ± 5	9	1.60 ± 0.20	18	310 ± 3	34	0.85	6
**10e**	778 ± 6	12	1.75 ± 0.15	19	325 ± 3	36	0.70	7
Staurosporine	465 ± 4	7	1.50 ± 0.10	17	288 ± 2	32	1.20	4
Control	65	1	0.09	1	9	1	5.00	1

When MCF-7 cells were treated with compound **10e** at its IC_50_ concentration, it greatly increased the levels of activated caspases 3 and 8. Table 3 shows a 12-fold increase in active caspase-3 expression and a 19-fold increase in active caspase-8 expression. Upon treatment with compound **6i**, the levels of caspase-3 and caspase-8 increase significantly—by 9 and 18 times, respectively, compared to untreated cells. In every instance, compounds **6i** and **10e** demonstrated superior efficacy as activators of caspase-3 and caspase-8 compared to the reference staurosporine.

##### 2.2.4.2 Assays for the proapoptotic Bax and anti-apoptotic Bcl-2

The current study supplied compounds **6i** and **10e** to breast (MCF-7) cancer cell lines at their respective IC_50_ values. This led to a notable elevation of pro-apoptotic Bax expression, with a fold increase of 34 for compound **6i** and 36 for compound **10e**. Also, the treatment significantly decreased anti-apoptotic Bcl-2 expression levels, with a reduction of approximately 6-fold for compound **6i** and 7-fold for compound **10e**. The results are given in Table 3. Compounds **6i** and **10e** significantly increased the Bax/Bcl-2 ratio relative to the control untreated cells. These data suggest that apoptosis may be one of the factors contributing to the antiproliferative activity of these compounds.

### 2.3 Molecular modelling

**FIGURE 4:**
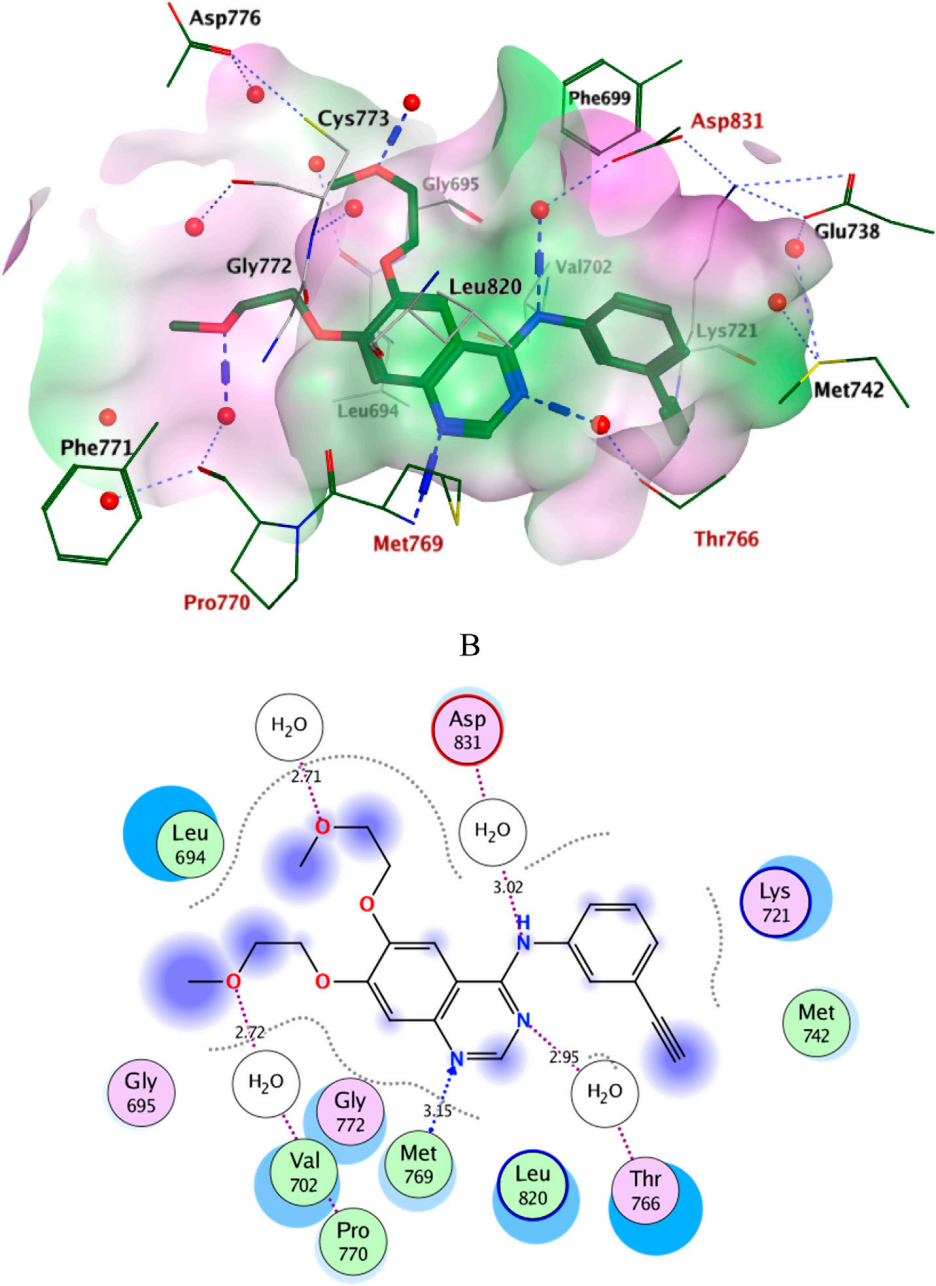
Ligplots at ATP-binding site of EGFR; **(A)** 3D-docked model of erlotinib (dark green) showing the protein lipophilicity surface (purple: hydrophilic, white: neutral, Green: lipophilic), **(B)** 2D-docked model of erlotinib. A.

**FIGURE 5:**
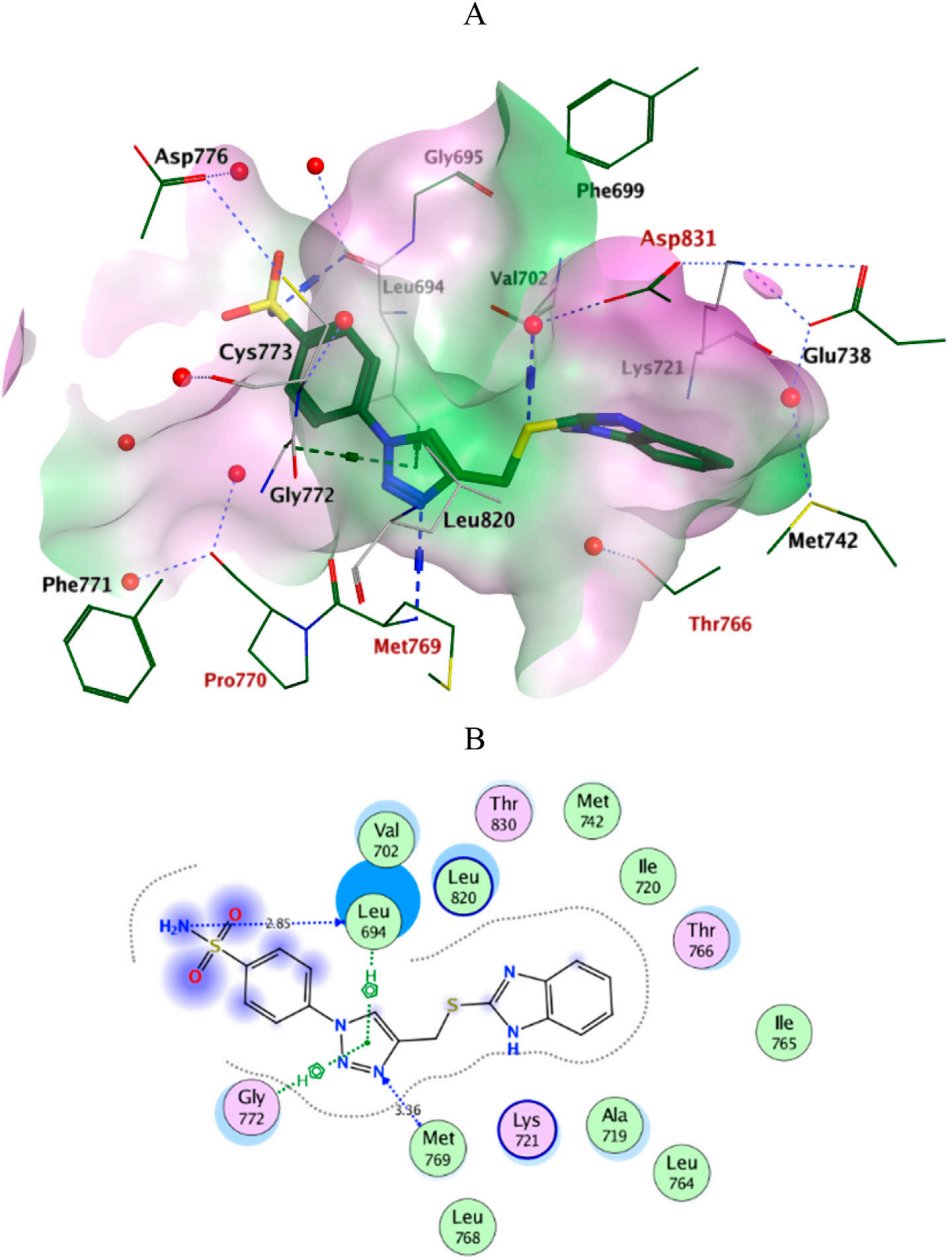
Ligplots at ATP-binding site of EGFR; **(A)** 3D-docked model of Scaffold A **6i** (dark green) showing the protein lipophilicity surface (purple: hydrophilic, white: neutral, Green: lipophilic), **(B)** 2D-docked model of **6i**. A.

**FIGURE 6:**
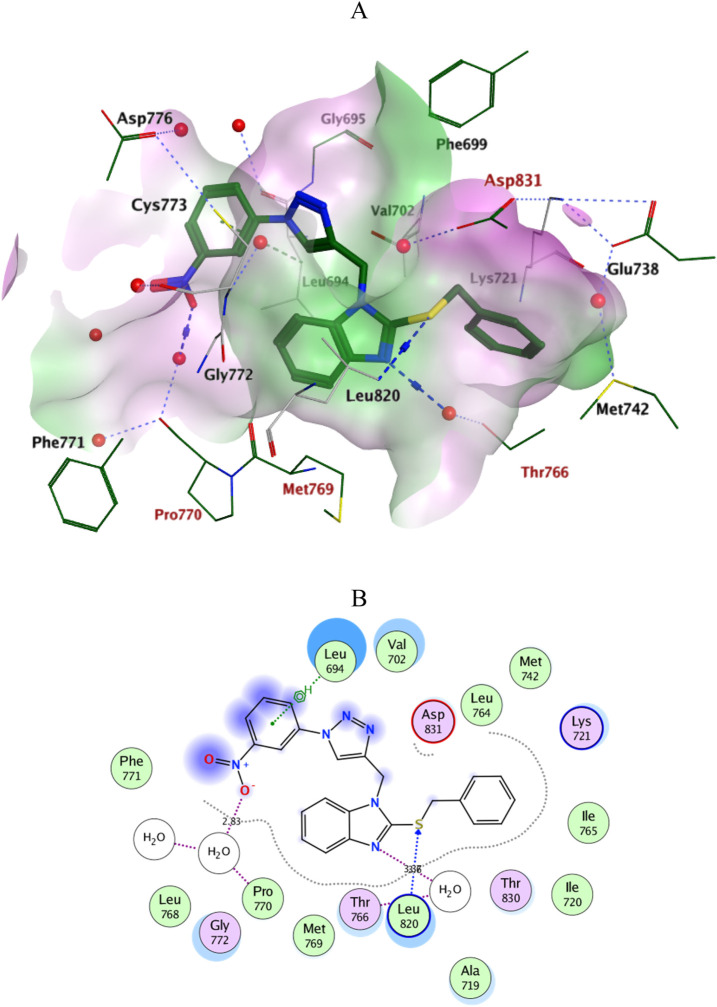
Ligplots at ATP-binding site of EGFR; **(A)** 3D-docked model of Scaffold B **10e** (dark green) showing the protein lipophilicity surface (purple: hydrophilic, white: neutral, Green: lipophilic), **(B)** 2D-docked model of **10e**.

Docking simulations of the new compounds **6a-i** and **10a-i** were performed at the ATP-binding site of EGFR to explore their potential binding modes and rationalize the biological results. The crystal structure of the EGFR in complex with the aniline-quinazoline inhibitor erlotinib (PDB: 1M17) (Stamos et al., 2002) was used in the present investigation. All minimizations were performed using the MOE force field (OPLS-AA) and the Born solvation model (Belal et al., 2022). Accuracy of the docking protocol was achieved by redocking the co-crystallized ligand into the EGFR binding site from where the docked ligand displayed an *RMSD* value of 0.96 Å (Figure 4). The new compounds exhibited good docking scores (−5.70 to −8.82 kcal/mol) relative to the erlotinib docking score of −11.80 kcal/mol. The docking results of the compounds were compared with erlotinib, and the results are shown in Table 4. Regarding Scaffold A (**6a-i**), the ligand 2-thiobenzimidazole inserts into the hydrophobic pocket in alignment with the erlotinib phenylacetylene moiety, forming stacking between Lys721 and Thr766. Also, the sulfur atom forms a similar water-bridged H-bond with Asp831 at the DFG motif as the NH spacer of the reference erlotinib. In addition, the ligand triazole methylene moiety occupies the location of erlotinib quinazoline, forming pi-H contacts with Leu694 and Gly772. The triazole nitrogen of the most active derivatives (**6e** and **6i**) accepts an additional H bond from the backbone NH of the key amino acid Met769, similar to the quinazoline N-1 of erlotinib. The substituted-phenyl tail projects past an ether chain of the erlotinib, forming pi-H interactions with Leu694 at the gate of the binding site. Interestingly, the amino sulfonyl group of the utmost active derivative **6i** donates unique H-bond contact to Leu694 (Figure 5). Meanwhile, compound **6e** probes an opposite orientation within the binding site, where the ligand 3-NO_2_-phenyl moiety is inserted into the hydrophobic pocket and accepts an H-bond contact from Thr766. The 2-thiobenzimidazole alternatively forms an H-bond with the amide carbonyl of Met769 and a pi-H contact between the aryl moiety and the Leu694 residue. On the other hand, scaffold B derivatives (**10a-i**) are better at probing the space of the binding site than scaffold A derivatives. The ligand benzyl group was inserted into the hydrophobic pocket in alignment with the erlotinib phenylacetylene moiety. Furthermore, the ligand 2-thiobenzimidazole ring is lying in the location of the erlotinib quinazoline moiety close to the amino acid residue Met769. In the complexes of the unsubstituted compound **10a** and the 4-methoxy compound **10g**, the benzimidazole nitrogen accepts an H-bond from Met769 amide nitrogen compared to the H‐bond with the quinazoline N-1 of erlotinib. At the same time, the nitro-containing derivatives (**10d** and **10e**) form a water-bridged H-bond with Thr766, similar to the erlotinib quinazoline N-3. Also, the sulfur atom of these derivatives forms an additional H-bond interaction with Leu820. The substituted-phenyl triazole tail projects past the erlotinib ether chains, forming pi-H interactions with Leu694 at the gate of the binding site. Besides, the 3-NO_2_ moiety of the utmost active derivative **10e** forms a water-bridged H-bond with Pro770, similar to the ether chain of erlotinib at the gate of the binding site (Figure 6). By shifting the methoxy group to position 4 at the phenyl ring in the **10d** protein complex, the interaction with the amino acid residue Pro770 at the binding gate is lost. Moreover, the triazole ring in the **10c**, **10g**, and **10i** complexes forms a water-bridged pi-H contact with Cys773 at the binding gate. According to the docking simulation results, EGFR might be a plausible target for the antiproliferative action of novel scaffolds.

**TABLE 4:** Ligand-protein complex interactions of the tested compounds **6a-i** and **10a-i** within the ATP-binding site of EGFR.

Compd	S Score	H-bond interactions (Å)	Hydrophobic interactions	Other interactions (Å)
^a^ **AQ4**	−11.80	Asp831: HOH (3.02) HOH (2.71) Pro770: HOH (2.72) Met769 (3.15) Thr766: HOH (2.95)	Ala719, Met769, Thr766, Lys721, Leu820, Leu694, Val702, Gly772, Thr830	—
**6a**	−7.95	Asp831: HOH (3.43)	Leu694, Val702, Gly772, Ala719, Met769, Thr766, Lys721, Leu820, Thr830	Leu694: pi-H (4.10, 3.67) Gly772: pi-H (3.86)
**6b**	−7.99	Asp831: HOH (3.33)	Leu694, Val702, Gly772, Ala719, Met769, Thr766, Lys721, Leu820, Thr830	Leu694: pi-H (4.14, 3.72) Gly772: pi-H (3.81)
**6c**	−7.98	Asp831: HOH (3.33)	Leu694, Val702, Gly772, Ala719, Met769, Thr766, Lys721, Leu820, Thr830	Leu694: pi-H (4.16, 3.72) Gly772: pi-H (3.81)
**6d**	−8.02	Asp831: HOH (3.25)	Leu694, Val702, Gly772, Ala719, Met769, Thr766, Lys721, Leu820, Thr830	Leu694: pi-H (4.11, 3.75) Gly772: pi-H (3.74)
**6e**	−8.82	Met769 (3.43, 3.33) Thr766 (3.04)	Leu694, Val702, Gly772, Ala719, Met769, Thr766, Lys721, Leu820, Thr830	Leu694: pi-H (3.79)
**6f**	−7.89	Asp831: HOH (3.32)	Leu694, Val702, Gly772, Ala719, Met769, Thr766, Lys721, Leu820, Thr830	Leu694: pi-H (4.10, 3.72) Gly772: pi-H (3.82)
**6g**	−8.13	Asp831: HOH (3.36)	Leu694, Val702, Gly772, Ala719, Met769, Thr766, Lys721, Leu820, Thr830	Leu694: pi-H (4.23, 3.76, 4.31) Gly772: pi-H (3.77)
**6h**	−8.27	Asp831: HOH (3.21)	Leu694, Val702, Gly772, Ala719, Met769, Thr766, Lys721, Leu820, Thr830	Leu694: pi-H (3.73) Cys773: HOH: pi-H (3.84)
**6i**	−7.41	Asp831: HOH (3.37) Leu694 (2.85) Met769 (3.36)	Leu694, Val702, Gly772, Ala719, Met769, Thr766, Lys721, Leu820, Thr830	Leu694: pi-H (4.10) Gly772: pi-H (4.36)
**10a**	−6.61	Thr766 (3.93) Met769 (3.09)	Leu694, Val702, Gly772, Ala719, Met769, Thr766, Lys721, Leu820, Thr830	Leu694: pi-H (3.61)
**10b**	−5.70	—	Leu694, Val702, Gly772, Ala719, Met769, Thr766, Lys721, Leu820, Thr830	Leu694: pi-H (3.78)
**10c**	−7.29	—	Leu694, Val702, Gly772, Ala719, Met769, Thr766, Lys721, Leu820, Thr830	Leu694: pi-H (3.92) Val702: pi-H (4.04) Cys773: HOH pi-H (3.21)
**10d**	−8.05	Leu820 (3.67) Thr766: HOH (3.07)	Leu694, Val702, Gly772, Ala719, Met769, Thr766, Lys721, Leu820, Thr830	Val702 (4.25)
**10e**	−8.17	Leu820 (3.60) Thr766: HOH (3.37) Pro770: HOH (2.83)	Leu694, Val702, Gly772, Ala719, Met769, Thr766, Lys721, Leu820, Thr830	Leu694 pi-H (4.46)
**10f**	−7.54	—	Leu694, Val702, Gly772, Ala719, Met769, Thr766, Lys721, Leu820, Thr830	Leu694 pi-H (3.78)
**10g**	−8.54	Met769 (3.07)	Leu694, Val702, Gly772, Ala719, Met769, Thr766, Lys721, Leu820, Thr830	Leu694 pi-H (3.63) Cys773: HOH pi-H (3.91)
**10h**	−7.87	Leu820 (3.59)	Leu694, Val702, Gly772, Ala719, Met769, Thr766, Lys721, Leu820, Thr830	Leu694 pi-H (3.83) Val702 pi-H (4.37)
**10i**	−6.16	Pro770: HOH (2.91) Pro770: HOH (2.66)	Leu694, Val702, Gly772, Ala719, Met769, Thr766, Lys721, Leu820, Thr830	Leu694 pi-H (3.79) Cys773: HOH pi-H (3.58)

^a^ AQ4, erlotinib.

### 2.4 *In silico* ADMET/pharmacokinetics studies

The ADMET properties of compounds **6i** and **10e** were predicted using the pkCSM-pharmacokinetics server (http://biosig.unimelb.edu.au/pkcsm/)by generating SMILES (Simplified Molecule Input Line Entry Specification) of the compounds using ChemDraw software (Pires et al., 2015). The compound’s efficacy as an orally active drug is determined using Caco2 permeability and intestinal absorption models. Both compounds obey Lipinski’s rules of five with zero violation, Table 5. The scaffold B derivative **10e** displays a higher Caco2 permeability value and demonstrates superior intestinal absorption ability with 100% than the scaffold A compound **6i**. The second variable in absorption is skin permeability, and both compounds have permeability values of less than −2.5 log Kp, suggesting poor permeability. P-glycoprotein is a factor of the ATP-binding cassette (ABC) transporter, essential for the active molecular transport across cell membranes. Both compounds are predicted to be P-glycoprotein substrates, suggesting they can move through the cell membrane via the ABC transporter. Besides this, both compounds were effective as inhibitors for P-glycoprotein II transporters. In contrast, only **10e** was effective as an inhibitor for the P-glycoprotein I transporters, implying that **6i** cannot inhibit type II drug efflux pumps. The VDss assay estimates the total amount of drug needed for uniform drug distribution in the blood. However, both compounds show low VDss values, indicating slower diffusion in blood. The compound’s ability to move to the brain can be determined via the permeability of the Blood-Brain Barrier (BBB). They will be poorly distributed to the brain if the logBB values are less than −1. Thus, both compounds might be unable to cross BBB. The blood-brain permeability surface area product (logPS) has a more direct and accurate estimation. Both compounds have logPS > −3 and can penetrate the CNS. The metabolism of the compounds in the body was predicted using seven different cytochrome models. Both Compounds are likely to be metabolized by CYP3A4 and also going to be CYP1A2 and CYP2C9 inhibitors. The predicted total clearance rates for the compounds are shown in Table 6, and only **10e** appeared as a substrate for the organic cation transporter 2 (OCT2). Furthermore, only **10e** predicted AMES toxicity, suggesting this compound might have carcinogenicity or mutagenicity. Also, both compounds will likely be hERG II inhibitors without effect on hERG I. Skin sensitization and hepatotoxicity were not seen in compound **10e,** while **6i** might show hepatotoxicity. The toxic effects of compounds are shown in Table 6, along with other ADMET properties.

**TABLE 5:** Physicochemical properties of compounds **6i** and **10e**.

Descriptor	6i	10e
Molecular weight	386.46	442.50
LogP	2.0833	4.8658
#Rotatable bonds	5	7
#Acceptors	7	8
#Donors	2	0
Surface area	153.04	187.22

**TABLE 6:** ADMET properties of compounds **6i** and **10e**.

Property	Model name	Predicted value		Unit
		6i	10e	
Absorption	Water solubility	−2.941	−2.903	Numeric (log mol/L)
	Caco2 permeability	0.068	0.739	Numeric (log Papp in 10 cm/s)
	Intestinal absorption (human)	78.249	100	Numeric (% Absorbed)
	Skin permeability	−2.736	−2.735	Numeric (log Kp)
	P-glycoprotein substrate	yes	Yes	Categorical (Yes/No)
	P-glycoprotein I inhibitor	No	Yes	Categorical (Yes/No)
	P-glycoprotein II inhibitor	yes	Yes	Categorical (Yes/No)
Distribution	VDss (human)	−0.234	−0.223	Numeric (log L/kg)
	Fraction unbound (human)	0.101	0.278	Numeric (Fu)
	BBB permeability	−1.162	−1.089	Numeric (log BB)
	CNS permeability	−2.785	−2.056	Numeric (log PS)
Metabolism	CYP2D6 substrate	No	No	Categorical (Yes/No)
	CYP3A4 substrate	Yes	Yes	Categorical (Yes/No)
	CYP1A2 inhibitor	Yes	Yes	Categorical (Yes/No)
	CYP2C19 inhibitor	No	Yes	Categorical (Yes/No)
	CYP2C9 inhibitor	Yes	Yes	Categorical (Yes/No)
	CYP2D6 inhibitor	No	No	Categorical (Yes/No)
	CYP3A4 inhibitor	No	Yes	Categorical (Yes/No)
Excretion	Total clearance	0.559	0.366	Numeric (log mL/min/kg)
	Renal OCT2 substrate	No	Yes	Categorical (Yes/No)
Toxicity	AMES toxicity	No	Yes	Categorical (Yes/No)
	Max. tolerated dose (human)	−0.124	0.262	Numeric (log mg/kg/day)
	hERG I inhibitor	No	No	Categorical (Yes/No)
	hERG II inhibitor	Yes	Yes	Categorical (Yes/No)
	Oral Rat Acute Toxicity (LD50)	2.738	2.478	Numeric (mol/kg)
	Oral Rat Chronic Toxicity (LOAEL)	0.73	0.418	Numeric (log mg/kg. bw/day)
	Hepatotoxicity	Yes	No	Categorical (Yes/No)
	Skin sensitization	No	No	Categorical (Yes/No)
	T. Pyriformis toxicity	0.285	0.285	Numeric (log ug/L)
	Minnow toxicity	1.329	−4.772	Numeric (log mM)

## 3 Conclusion

This study presents the design and synthesis of novel benzimidazole/1,2,3-triazole hybrids that can block the EGFR enzyme. Compounds **6i** and **10e** were identified as the most compelling due to their potent EGFR inhibition, with IC_50_ values of 78 and 73 nM, respectively. Furthermore, they exhibited possible antiproliferative properties against the MCF-7 breast cancer cell line (IC_50_ = 28 and 24 nM, respectively). At a dose of 50 μM, compounds **6i** and **10e** exhibited no impact on non-tumor cells MCF-10A, suggesting the potential tumor-cell selectivity of these derivatives. Molecular docking experiments have effectively illustrated the unique binding interactions of compounds **6i** and **10e** with the EGFR active site. This comprehensive examination is essential for comprehending their mode of action as EGFR inhibitors. The thorough evaluation of these hybrids’ absorption, distribution, metabolism, and excretion (ADME) characteristics highlights their potential as therapeutic agents. The findings indicate that **6i** and **10e** are attractive candidates for the development of novel medicines for cancer therapy. Furthermore, further exploration of the mechanism of action, *in vivo* carcinogenic animal models, and lead optimization is underway in our laboratory.

## 4 Experimental

### 4.1 Chemistry

#### 4.1.1 Materials and methods

See Supplementary Appendix A Compounds **2**, **3**, **5a-i**, **8**, and **9** were prepared according to reported procedures (Latif et al., 2021; Dunga et al., 2022; Kutonova et al., 2013; Kalyani and Manikyamba, 2004).

#### 4.1.2 General method for the synthesis of compounds 6a-i and 10a-i

To a stirred solution of the compound **3** or **9** (1 eq.) in a mixture of 10 mL THF and 10 mL H_2_O, the appropriate azide derivatives **5a-i** (1.5 eq.) were added. The mixture was stirred for 45 min. Then sodium ascorbate (0.05 g, 0.2 eq.) was added initially, followed by the addition of CuSO4 (0.08 g, 0.1 eq.) to the reaction mixture and continued stirring till the completion of the reaction (monitored by TLC). Compounds **6a-i** were purified by column chromatography using silica gel eluted gradually with hexane: EtOAc (100:0 to 30:70, v/v). Compounds **10a-i** were purified by recrystallization using DMF: H_2_O (1:2).

##### 4.1.2.1 2-(((1-Phenyl-1*H*-1,2,3-triazol-4-yl)methyl)thio)-1*H*-benzimidazole (6a)

Yield: 0.48 g (56%), Yellow powder, m. p: 137°C–138°C, *R*_*f*_: 0.3 (hexane: ethyl acetate, 1:1, v/v); ^1^H NMR (400 MHz, DMSO-*d*_6_): δ = 12.95 (br s, 1H, NH), 8.79 (s, 1H; triazole CH), 7.82 (t, *J* = 8.6 Hz, 2H, Ar-H), 7.68 (d, *J* = 7.7 Hz, 1H, Ar-H), 7.61–7.52 (m, 2H, Ar-H), 7.48 (d, *J* = 7.3 Hz, 2H, Ar-H), 7.28–7.05 (m, 2H, ArH-5.6), 4.79 (s, 2H, SCH_2_). ^13^C NMR (101 MHz, DMSO-*d*_6_) δ 143.0, 136.3, 129.8, 128.7, 122.0, 121.8, 120.0, 117.8, 109.2, 26.8. Anal. Calc. (%) for C_16_H_13_N_5_S: C, 62.52; H, 4.26; N, 22.78; S, 10.43. Found: C, 62.35; H, 4.47; N, 23.05; S, 10.59.

##### 4.1.2.2 2-(((1-(4-Chlorophenyl)-1*H*-1,2,3-triazol-4-yl)methyl)thio)-1*H*-benzimidazole (6b)

Yield: 0.24 g (89%), Yellow powder, m. p: 145°C–147°C, *R*_*f*_: 0.35 (hexane: ethyl acetate, 1:1, v/v); ^1^H NMR (400 MHz, DMSO-*d*_6_): δ = 12.71 (br s, 1H, NH), 8.78 (s, 1H; triazole CH), 7.91 (d, *J* = 8.8 Hz, 2H, ArH-3′,5′), 7.65 (d, *J* = 8.8 Hz, 2H, Ar-H-2′,6′), 7.51–7.44 (m, 2H, ArH-4.7), 7.24–7.10 (m, 2H, ArH-5.6), 4.71 (s, 2H; SCH_2_). ^13^C NMR (101 MHz, DMSO-*d*_6_) δ 144.6, 135.3, 132.9, 129.8, 127.1, 121.9, 121.7, 121.5, 117.9, 25.8. Anal Calc. (%) for: C_16_H_12_ClN_5_S C,56.22; H, 3.54; N, 20.49; S,9.38 Found: C,56.49; H,3.66; N,20.73; S,9.41.

##### 4.1.2.3 2-(((1-(4-Bromophenyl)-1*H*-1,2,3-triazol-4-yl)methyl)thio)-1*H*-benzimidazole (6c)

Yield: 0.52 g (51%), Yellow powder, m. p: 168°C–170°C, *R*_*f*_: 0.41 (hexane: ethyl acetate, 1:1, v/v); ^1^H NMR (400 MHz, DMSO-*d*_6_): δ = 12.64 (br s, 1H, NH), 8.78 (s, 1H, triazole CH), 7.85 (d, *J* = 9.0 Hz, 2H, Ar-H -3′,5′), 7.78 (d, *J* = 9.0 Hz, 2H, Ar-H-2′,6′), 7.54–7.43 (m, 2H, Ar-H-4.7), 7.14 (dd, *J* = 6.0, 3.2 Hz, 2H, Ar-H-5.6), 4.72 (s, 2H, SCH_2_). ^13^C NMR (101 MHz, DMSO-*d*_6_) δ 149.2, 144.6, 143.3, 142.9, 135.7, 127.3, 121.9, 121.8, 121.3, 110.1, 25.8. Anal. Calc. (%) for: C_16_H_12_BrN_5_S C, 49.75; H, 3.13 N, 18.13; S, 8.30, Found: C,49.93; H,3.25; N, 18.40; S, 8.25.

##### 4.1.2.4 2-(((1-(4-Nitrophenyl)-1*H*-1,2,3-triazol-4-yl)methyl)thio)-1*H*-benzimidazole (6d)

Yield: 0.52 (59%), Yellow powder, m. p:162°C–164°C, *R*_*f*_: 0.28 (hexane: ethyl acetate, 1:1, v/v); IR (KBr, ύ cm^-1^): 3,400 (NH), 3,084 (=CH), 2,962 (CH_2_), 1,640, 1,598 (C=N, C=C), 1,523, 1,340 (NO_2_), 854 (*p*-bending). ^1^H NMR (400 MHz, DMSO-*d*_*6*_): δ = 12.64 (br s, 1H, NH), 8.97 (s, 1H, triazole CH), 8.43 (d, *J* = 9.2 Hz, 2H, Ar-H-3′,5′), 8.20 (d, *J* = 9.2 Hz, 2H, Ar-H-2′,6′), 7.61–7.52 (m, 1H, Ar-H), 7.44–7.35 (m, 1H, Ar-H), 7.21–7.08 (m, 2H, Ar-H-5.6), 4.74 (s, 2H, SCH_2_). ^13^C NMR (101 MHz, DMSO-*d*_6_) δ 149, 146.6, 145.2, 140.7, 125.5, 122.2, 121.2, 120.6, 117.5, 110.4, 25.7. Anal Calc. (%) for C_16_H_12_N_6_O_2_S: C, 54.54; H, 3.43; N, 23.85; S, 9.10 Found: C,54.71; H,3.54; N,24.01; S,9.23.

##### 4.1.2.5 2-(((1-(3-Nitrophenyl)-1*H*-1,2,3-triazol-4-yl)methyl)thio)-1*H*-benzimidazole (6e)

Yield: 0.1 g (56%) Yellow powder, m. p: 123°C–125°C, *R*_*f*_: 0.32 (hexane: ethyl acetate, 1:1, v/v). ^1^H NMR (400 MHz, DMSO-*d*_6_): δ = 12.64 (br s, 1H, NH), 8.99 (s, 1H, triazole CH), 8.68 (s, 1H, Ar-H), 8.35 (dd, *J* = 8.2, 2.1 Hz, 1H, Ar-H), 8.29 (dd, *J* = 8.2, 2.2 Hz, 1H, Ar-H), 7.86 (t, *J* = 8.1 Hz, 1H, Ar-H), 7.66–7.50 (m, 1H, Ar-H), 7.43–7.32 (m, 1H, Ar-H), 7.12 (dd, *J* = 6.2, 3.0 Hz, 2H, Ar-H), 4.74 (s, 2H, SCH_2_). ^13^C NMR (101 MHz, DMSO-*d*_6_): δ 148.9, 145.2, 144.0, 137.5, 131.9, 126.5, 123.6, 122.7, 122.3, 118.4, 115.2, 110.6, 27.3. Anal. Calc. (%) for C_16_H_12_N_6_O_2_S: C,54.54; H, 3.43; N, 23.85; S,9.10, Found: C,54.70; H,3.52; N,24.09; S,9.17.

##### 4.1.2.6 2-(((1-(*p*-Tolyl)-1*H*-1,2,3-triazol-4-yl)methyl)thio)-1*H*-benzimidazole (6f)

Yield: 0.8 g (94%), Yellow powder, m. p: 134–136̊C, *R*_*f*_: 0.21 (hexane: ethyl acetate, 1:1, v/v). ^1^H NMR (400 MHz, DMSO-*d*_6_): δ = 12.64 (br s, 1H, NH), 8.69 (s, 1H, triazole CH), 7.73 (d, *J* = 8.3 Hz, 2H, Ar-H-3′,5′), 7.48 (brs, 2H, Ar-H), 7.37 (d, *J* = 8.1 Hz, 2H, Ar-H), 7.18–7.08 (m, 2H, Ar-H-5.6), 4.71 (s, 2H, SCH_2_), 2.37 (s, 3H, CH_3_). ^13^C NMR (101 MHz, DMSO-*d*_6_) δ 149.2, 144.2, 138.5, 134.3, 130.3, 121.7, 121.3, 120.0, 117.5, 110.5, 26.9, 21.1. Anal. Calc. (%) for C_17_H_15_N_5_S: C, 63.53; H, 4.70 N, 21.79; S, 9.98, Found: C, 63.42; H, 4.88; N, 22.06; S, 10.05.

##### 4.1.2.7 2-(((1-(4-Methoxyphenyl)-1*H*-1,2,3-triazol-4-yl)methyl)thio)-1*H*-benzimidazole (6g)

Yield: 0.5 g (56%), Yellow powder, m. p: 131–135̊C, *R*_*f*_: 0.2 (hexane: ethyl acetate, 1:1, v/v). ^1^H NMR (400 MHz, DMSO-*d*_6_): δ = 12.62 (brs, 1H, NH), 8.63 (s, 1H, triazole CH), 7.75 (d, *J* = 9.0 Hz, 2H, Ar-H-3′,5′), 7.72–7.65 (m, 1H,Ar-H), 7.55 (brs, 1H, Ar-H), 7.37 (bs, 1H,Ar-H), 7.18–7.01 (m, 3H, Ar-H), 4.69 (s, 2H, SCH_2_), 2.51 (s, 3H, OCH_3_). ^13^C NMR (101 MHz, DMSO-*d*_6_) δ 162, 159.2, 158.9, 155.0, 147.1, 144.0, 128.8, 125.6, 121.6, 114.6, 55.2, 26.7. Anal. Calc. (%) for C_17_H_15_N_5_OS: C, 60.52; H, 4.48 N, 20.76; S, 9.50, Found: C,60.41; H,4.30; N, 21.03; S, 9.61.

##### 4.1.2.8 2-(((1-(3-Methoxyphenyl)-1*H*-1,2,3-triazol-4-yl)methyl)thio)-1*H*-benzimidazole (6h)

Yield: 0.3 g (60%), Yellow powder, m. p:131-132̊C, *R*_*f*_: 0.37 (hexane: ethyl acetate, 1:1, v/v); ^1^H NMR (400 MHz, DMSO-*d*_6_): δ = 13.09 (br s,1H, NH), 8.82 (s, 1H, triazole CH), 7.68 (d, *J* = 7.4 Hz, 1H, Ar-H), 7.49–7.27 (m, 4H, Ar-H), 7.23–7.13 (m, 1H, Ar-H), 7.10–7.00 (m, 2H, Ar-H), 4.81 (s, 2H, SCH_2_), 3.82 (s, 3H, OCH_3_). ^13^C NMR (101 MHz, DMSO-*d*_6_) δ 160.0, 142.9, 137.3, 130.7, 122.0, 121.9, 117.8, 114.4, 111.9, 110.2, 105.6, 103.6, 55.5, 26.8. Anal. Calc. (%) for C_17_H_15_N_5_OS: C, 60.52; H, 4.48 N, 20.76; S,9.50 Found: C,60.34; H,4.59; N,20.94; S, 9.61.

##### 4.1.2.9 4-(4-(((1*H*-benzimidazol-2-yl)thio)methyl)-1*H*-1,2,3-triazol-1-yl)benzene-sulfonamide (6i)

Yield: 0.1 g (32%), Yellow powder, m. p: 233°C–234°C, *R*_*f*_: 0.075 (hexane: ethyl acetate, 1:1, v/v); ^1^H NMR (400 MHz, DMSO-*d*_6_): δ = 12.64 (brs, 1H, NH), 8.85 (s, 1H, triazole CH),8.09 (d, *J* = 8.4 Hz, 2H, Ar-H-3′,5′), 7.99 (d, *J* = 8.4 Hz, 2H; Ar-H-2′,6′), 7.62–7.53 (m, 1H, Ar-H), 7.51 (s, 2H, NH_2_), 7.42–7.34 (m, 1H, Ar-H), 7.18–7.09 (m, 2H, Ar-H-5.6), 4.73 (s, 2H, SCH_2_). ^13^C NMR (101 MHz, DMSO-*d*_6_) δ 145.3, 144.3, 138.9, 127.9, 122.5, 122.2, 121.6, 120.8, 118.0, 110.9, 26.3. Anal. Calc. (%) for C_16_H_14_N_6_O_2_S_2_: C, 49.73; H, 3.65; N, 21.75; S,16.59 Found: C,50.02; H,3.74; N,21.97; S,16.45.

##### 4.1.2.10 2-(Benzylthio)-1-((1-phenyl-1*H*-1,2,3-triazol-5-yl)methyl)-1*H*-benzimidazole (10a)

Yield: 0.06 g (43%), Yellow powder, m. p: 139°C–140°C, *R*_*f*_: 0.575 (hexane: ethyl acetate, 1:1, v/v); ^1^H NMR (400 MHz, DMSO-*d*_6_): δ = 8.78 (s, 1H, triazole CH), 7.83 (d, *J* = 7.7 Hz, 2H, Ar-H), 7.64 (d, *J* = 7.0 Hz, 1H, Ar-H), 7.62–7.54 (m, 3H, Ar-H), 7.52–7.41 (m, 3H, Ar-H)), 7.36–7.13 (m, 5H, Ar-H), 5.49 (s, 2H, N-CH_2_), 4.62 (s, 2H, S-CH_2_). ^13^C NMR (101 MHz, DMSO-*d*_6_) δ 143.5, 143.3, 137.7, 136.8, 136.3, 130.3, 129.4, 129.2, 128.9, 127.9, 122.2, 120.6, 118.2, 110.5, 39.1, 36.5. Anal. Calc. (%) for: C_23_H_19_N_5_S: C, 69.50; H, 4.82; N,17.62; S,8.07, Found: C,69.67; H,5.01; N,17.54; S,7.98.

##### 4.1.2.11 2-(Benzylthio)-1-((1-(4-chlorophenyl)-1*H*-1,2,3-triazol-5-yl)methyl)-1*H*-benzimidazole (10b)

Yield 0.25 g (33%), Yellow powder, m. p: 149°C–150°C, *R*_*f*_: 0.78 (hexane: ethyl acetate, 1:1, v/v); ^1^H NMR (400 MHz, DMSO-*d*_*6*_): δ = 8.80 (s, 1H, triazole CH), 7.88 (d, *J* = 8.4 Hz, 2H, Ar-H-4.7), 7.64 (d, *J* = 8.5 Hz, 4H, Ar-H), 7.45 (d, *J* = 7.3 Hz, 2H, Ar-H), 7.33–7.18 (m, 5H, Ar-H), 5.49 (s, 2H, N-CH_2_ (, 4.62 (s, 2H, S-CH_2_). ^13^C NMR (101 MHz, DMSO-*d*_6_) δ 143.7, 143.3, 137.6, 135.6, 133.5, 130.3, 129.4, 128.9, 127.9, 122.4, 122.2, 118.2, 110.5, 39.1, 36.5. Anal. Calc. (%) for: C_23_H_18_ClN_5_S: C,63.96; H, 4.20 N,16.21; S,7.42 Found: C,64.15; H,4.37; N,16.49; S,7.55.

##### 4.1.2.12 2-(Benzylthio)-1-((1-(4-bromophenyl)-1*H*-1,2,3-triazol-5-yl)methyl)-1*H*-benzimidazole (10c)

Yield 0.25 g (30%), Yellow powder, m. p:158°C–159°C, *R*_*f*_: 0.81 (hexane: ethyl acetate, 1:1, v/v); ^1^H NMR (400 MHz, DMSO-*d*_*6*_): δ = 8.80 (s, 1H, triazole CH), 7.87 (d, *J* = 8.4 Hz, 2H, Ar-H-3 ‶,5 ‶), 7.68–7.57 (m, 4H, Ar-H), 7.45 (d, *J* = 7.3 Hz, 2H, Ar-H), 7.40–7.14 (m, 5H, Ar-H), 5.49 (s, 2H, N-CH_2_(, 4.62 (s, 2H, S-CH_2_). ^13^C NMR (101 MHz, DMSO-*d*_6_) δ 143.7, 137.7, 135.6, 133.5, 131, 130.2, 129.4, 128.9, 127.9, 122.3, 122.2, 118.2, 110.5, 39.3, 31.1. Anal. Calc. (%) for: C_23_H_18_BrN_5_S: C, 57.99; H, 3.81; N, 14.70; S, 6.73 Found: C,58.21; H,3.92; N,14.93; S,6.80.

##### 4.1.2.13 2-(Benzylthio)-1-((1-(4-nitrophenyl)-1*H*-1,2,3-triazol-5-yl)methyl)-1*H*-benzimidazole (10d)

Yield 0.25 g (32%), Yellow powder, m. p: 160°C–161°C, *R*_*f*_: 0.71 (hexane: ethyl acetate, 1:1, v/v); IR (KBr, ύ cm^-1^): IR (KBr, ύ cm^-1^): 3,085 (=CH), 2,925 (CH_2_), 1,597, 1,507 (C=N, C=C), 1,523, 1,340 (NO_2_), 854 (*p*-bending), ^1^H NMR (400 MHz, DMSO-*d*_6_): δ = 9 (s, 1H, triazole CH), 8.42 (d, *J* = 9.0 Hz, 2H, Ar-H-3 ‶,5 ‶), 8.17 (d, *J* = 8.9 Hz, 2H, Ar-H 2 ‶,6 ‶), 7.75–7.58 (m, 2H, Ar-H-4.7), 7.44 (d, *J* = 7.4 Hz, 2H, Ar-H), 7.33–7.18 (m, 5H, Ar-H), 5.53 (s, 2H, NCH_2_(,4.63 (s, 2H, S-CH_2_). ^13^C NMR (101 MHz, DMSO-*d*_6_) δ 147.2, 144.2, 143.4, 141.1, 137.7, 129.4, 128.9, 127.9, 126, 122.7, 122.4, 121.1, 118.2, 110.5, 39.1, 36.6. Anal. Calc. (%) for: C_23_H_18_N_6_O_2_S: C, 62.43; H, 4.10 N, 18.99; S,7.25 Found: C,62.31; H,4.28; N,19.05; S,7.32.

##### 4.1.2.14 2-(Benzylthio)-1-((1-(3-nitrophenyl)-1*H*-1,2,3-triazol-5-yl)methyl)-1*H*-benzimidazole (10e)

Yield 0.06 g (20%), Yellow powder, m. p:123°C–124°C, *R*_*f*_: 0.68 (hexane: ethyl acetate, 1:1, v/v); ^1^H NMR (400 MHz, DMSO-*d*_*6*_): δ = 8.93 (s, 1H, triazole CH), 8.62 (d, *J* = 7.9 Hz, 1H, Ar-H)**,** 8.29 (d, *J* = 7.9 Hz, 2H, Ar-H), 7.86 (t, *J* = 7.9 Hz, 2H, Ar-H), 7.62 (bs, 2H, Ar-H), 7.41 (d, *J* = 7.4 Hz, 2H, Ar-H), 7.25 (m, 4H, Ar-H), 5.49 (s, 2H, NCH_2_), 4.58 (s, 2H, SCH_2_). ^13^C NMR (101 MHz, DMSO-*d*_6_) δ 148.8, 143.8, 137.4, 137.2, 131.9, 129.2, 128.8, 127.8, 126.5, 123.6, 122.6, 122.6, 122.4, 118.1, 115.2, 110.5, 36.6, 29.2. Anal. Calc. (%) for: C_23_H_18_N_6_O_2_S: C, 62.43; H, 4.10; N,18.99; S,7.25, Found: C,62.60; H, 4.24; N, 19.17; S, 7.39.

##### 4.1.2.15 2-(Benzylthio)-1-((1-(*p*-tolyl)-1*H*-1,2,3-triazol-5-yl)methyl)-1*H*-benzimidazole (10f)

Yield 0.3 g (42%), Yellow powder, m. p:138°C–140°C, *R*_*f*_: 0.62 (hexane: ethyl acetate, 1:1, v/v), ^1^H NMR (400 MHz, DMSO-*d*_*6*_): δ = 8.71 (s, 1H, triazole CH), 7.70 (d, *J* = 8.4 Hz, 2H, Ar-H), 7.67–7.56 (m, 2H, Ar-H), 7.45 (d, *J* = 6.8 Hz, 2H, Ar-H), 7.37 (d, *J* = 8.2 Hz, 2H, Ar-H), 7.31–7.19 (m, 5H, Ar-H), 5.47 (s, 2H, NCH_2_(,4.62 (s, 2H, SCH_2_), 2.36 (s, 3H, CH_3_), ^13^C NMR (101 MHz, DMSO-*d*_*6*_) δ 143.4, 138.9, 137.7, 134.6, 130.6, 129.4, 128.9, 127.9, 122.3, 122.2, 122.1, 120.5, 118.2, 110.5, 36.5, 31.1, 21.0; Anal. Calc. (%) for: C_24_H_21_N_5_S: C, 70.05; H, 5.14; N,17.02; S,7.79 Found: C, 70.24; H, 5.22; N, 17.29; S, 8.07.

##### 4.1.2.16 2-(Benzylthio)-1-((1-(4-methoxyphenyl)-1*H*-1,2,3-triazol-5-yl)methyl)-1*H*-benzimidazole (10g)

Yield 0.1 g (34%), Yellow powder, 139°C–140°C *R*_*f*_: 0.525 (hexane: ethyl acetate, 1:1, v/v), ^1^H NMR (400 MHz, DMSO-d_6_): δ = 8.66 (s, 1H, triazole CH), 7.73 (d, *J* = 9.0 Hz, 2H, Ar-H), 7.65–7.56 (m, 2H, Ar-H-4.7), 7.49–7.42 (m, 2H, Ar-H), 7.34–7.15 (m, 5H, Ar-H), 7.10 (d, *J* = 9.0 Hz, 2H, Ar-H), 5.47 (s, 2H, NCH_2_), 4.62 (s, 2H, SCH_2_), 3.81 (s, 3H, OCH_3_). ^13^C NMR (101 MHz, DMSO-*d*_*6*_) δ 159.8, 143.3, 137.7, 131, 130.2, 129.4, 128.9, 127.9, 122.3, 122.3, 122.2, 118.2, 115.3, 110.5, 56.0, 39.3, 36.5. Anal. Calc. (%) for: C_24_H_21_N_5_OS: C, 67.43; H, 4.95; N,16.38; S,7.50 Found: C,67.61; H, 5.12; N,16.65; S,7.62.

##### 4.1.2.17 2-(Benzylthio)-1-((1-(3-methoxyphenyl)-1*H*-1,2,3-triazol-5-yl)methyl)-1*H*-benzimidazole (10h)

Yield 0.09 g (30%), Yellow powder, 118°C–119°C, *R*_*f*_: 0.24 (hexane: ethyl acetate, 1:1, v/v), ^1^H NMR (400 MHz, DMSO-d_6_): δ = 8.81 (s, 1H, triazole CH), 7.67–7.58 (m, 2H,Ar-H), 7.51–7.38 (m, 5H, Ar-H), 7.33–7.13 (m, 5H, Ar-H), 7.04 (d, *J* = 8.2 Hz, 1H, Ar-H), 5.49 (s, 2H, NCH_2_(, 4.63 (s, 2H, SCH_2_), 3.82 (s, 3H, OCH_3_). ^13^C NMR (101 MHz, DMSO-*d*_*6*_) δ 160.6, 143.5, 137.8, 137.6, 131.3, 129.4, 128.9, 127.9, 126.5, 122.4, 122.3, 118.2, 112.6, 110.6, 56.1, 39.3, 36.6. Anal. Calc. (%) for: C_24_H_21_N_5_OS: C, 67.43; H, 4.95; N, 16.38; S, 7.50, Found: C, 67.29; H, 5.06; N, 16.60; S, 7.61.

##### 4.1.2.18 4-(5-((2-(Benzylthio)-1*H*-benzo [d]imidazole-1-yl)methyl)-1*H*-1,2,3-triazol-1-yl) benzene-sulfonamide (10i)

Yield 0.3 g (38%), Yellow powder, m. p: 139°C–140°C, *R*_*f*_: 0.075 (hexane: ethyl acetate, 1:1, v/v); ^1^H NMR (400 MHz, DMSO-*d*_*6*_): δ = 8.88 (s, 1H, triazole CH), 8.07 (d, *J* = 8.7 Hz, 2H, Ar-H-2 ‶, 6 ‶), 7.99 (d, 2H, Ar-H-3 ‶,5 ‶), 7.69–7.58 (m, 2H, Ar-H), 7.52 (s, 2H, NH_2_), 7.45 (d, *J* = 6.9 Hz, 2H, Ar-H), 7.33–7.16 (m, 5H, Ar-H), 5.51 (s, 2H, NCH_2_(, 4.63 (s, 2H, S-CH_2_). ^13^C NMR (101 MHz, DMSO-*d*_6_) δ 144.4, 143.9, 138.8, 137.7, 129.4, 128.9, 127.9, 122.4, 122.4, 122.2, 120.8, 118.2, 110.5, 36.2, 31.1. Anal. Calc. (%) for: C_23_H_20_N_6_O_2_S_2_: C, 57.97; H, 4.23; N, 17.63; S, 13.45. Found: C, 58.24; H, 4.51; N, 17.89; S, 13.34.

### 4.2 Biology

#### 4.2.1 Cell viability assay

The normal human mammary gland epithelial (MCF-10A) cell line was employed to assess the viability of the evaluated substances. Cell viability was assessed using the MTT test following 4 days of incubation of MCF-10A cells with 50 µM of each examined compound (Mekheimer et al., 2022; Hisham et al., 2022). See Supplementary Appendix A for more details.

#### 4.2.2 Antiproliferative assay

The MTT assay was employed to examine the antiproliferative efficacy of **6a-i** and **10a-i** against four human cancer cell lines, utilizing erlotinib as a control (El-Sherief et al., 2019; Al-Wahaibi et al., 2022). Refer to Supplementary Appendix A for more information.

#### 4.2.3 EGFR inhibitory assay

The EGFR-TK assay evaluated the inhibitory efficacy of the most potent antiproliferative derivatives **6e**, **6i**, **10d**, **10e**, and **10g** against EGFR (Alshammari et al., 2022). Refer to Supplementary Appendix A for additional information.

#### 4.2.4 Apoptotic marker assays

Compounds **6i** and **10e** were assessed for their ability to activate caspase-3, caspase-8, and Bax, as well as to downregulate the anti-apoptotic protein Bcl2 in the MCF-7 breast cancer cell line (Abdelbaset et al., 2019). Supplementary Appendix A provides more details.

#### 4.2.5 Docking study

All the molecular modeling calculations and docking simulation studies were performed on a Processor Intel(R) Pentium(R) CPU N3510@ 1.99GHz and 4 GB Memory with Microsoft Windows 8.1 pro (64 Bit) operating system using Molecular Operating Environment (MOE 2019.0102, 2020; Chemical Computing Group, Canada) as the computational software (Stamos et al., 2002). Refer to Supplementary Appendix A for additional information.

#### 4.2.6 Calculations of *ADMET*

Pharmacokinetics and drug-likeness predictions for all newly synthesized compounds were conducted using the pkCSM-pharmacokinetics server (http://biosig.unimelb.edu.au/pkcsm/) developed by the Bio21 Institute University of Melbourne (Pires et al., 2015).

## Data Availability

The original contributions presented in the study are included in the article/Supplementary Material, further inquiries can be directed to the corresponding authors.
