# Supplementary material for: Design, synthesis, and apoptotic antiproliferative action of new benzimidazole/1,2,3-triazole hybrids as EGFR inhibitors
Source: Front Chem. 2025 Jan 13;12:1541846. doi: 10.3389/fchem.2024.1541846 (PMC11783063; doi:10.3389/fchem.2024.1541846)
Supplement: Supplementary file 1 [file Table1.docx]

**Supplementary Data**

**Design, synthesis, and apoptotic antiproliferative action of new benzimidazole/1,2,3-triazole hybrids as EGFR inhibitors**

Alshimaa A. Y. Ahmed**^1^,** Anber F. Mohammed^1^, Zainab M. Almarhoon^2^, Stefan Bräse^3^*, Bahaa G. M. youssif^1^*

^1^Pharmaceutical Organic Chemistry Department, Faculty of Pharmacy, Assiut University, Assiut 71526, Egypt; ^2^Department of Chemistry, College of Science, King Saud University, Riyadh 11451, Saudi Arabia; ^3^Institute of Biological and Chemical Systems, IBCS-FMS, Karlsruhe Institute of Technology, 76131 Karlsruhe, Germany.

**To whom correspondence should be addressed:*

**Bahaa G. M. Youssif**, Ph.D. Pharmaceutical Organic Chemistry Department, Faculty of Pharmacy, Assiut University, Assiut 71526, Egypt.

**Tel**.: (002)-01098294419

**E-mail address**: [bgyoussif2@gmail.com](mailto:bgyoussif2@gmail.com)

**S. Bräse**

Institute of Biological and Chemical Systems, IBCS-FMS, Karlsruhe Institute of Technology, 76131 Karlsruhe, Germany. E-mail: [braese@kit.edu](mailto:braese@kit.edu)

**^1^H NMR spectrum of (6a) :**

**
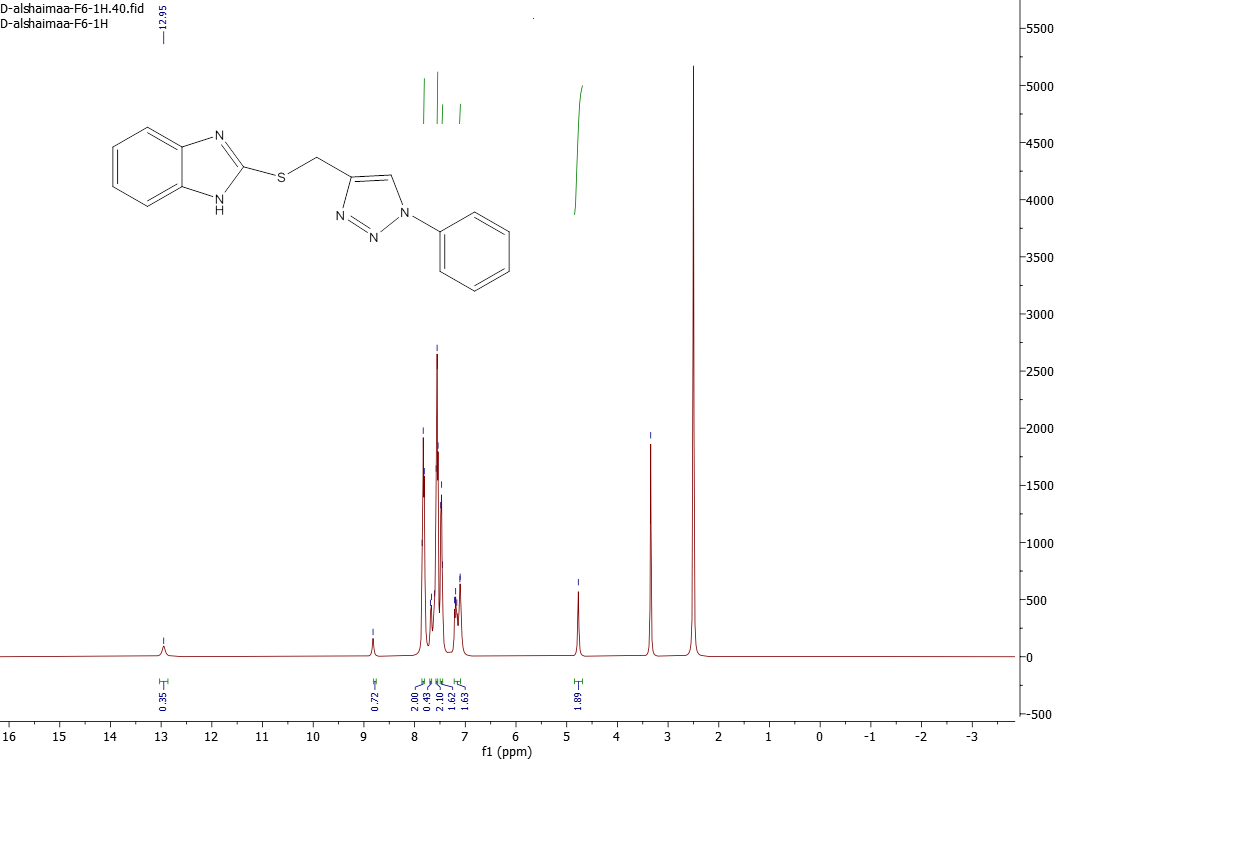
**

^1^H NMR (400 MHz, DMSO-*d*_6_): δ = 12.95 (br s, 1H, NH), 8.79 (s, 1H; triazole CH), 7.82 (t, *J* = 8.6 Hz, 2H, ArH), 7.68 (d, *J* = 7.7 Hz, 1H, Ar-H), 7.61 – 7.52 (m, 2H, Ar-H) , 7.48 (d, *J* = 7.3 Hz, 2H, Ar-H), 7.28 – 7.05 (m, 2H, ArH-5,6), 4.79 (s, 2H, SCH_2_)

**^13^C NMR spectrum of (6a)**


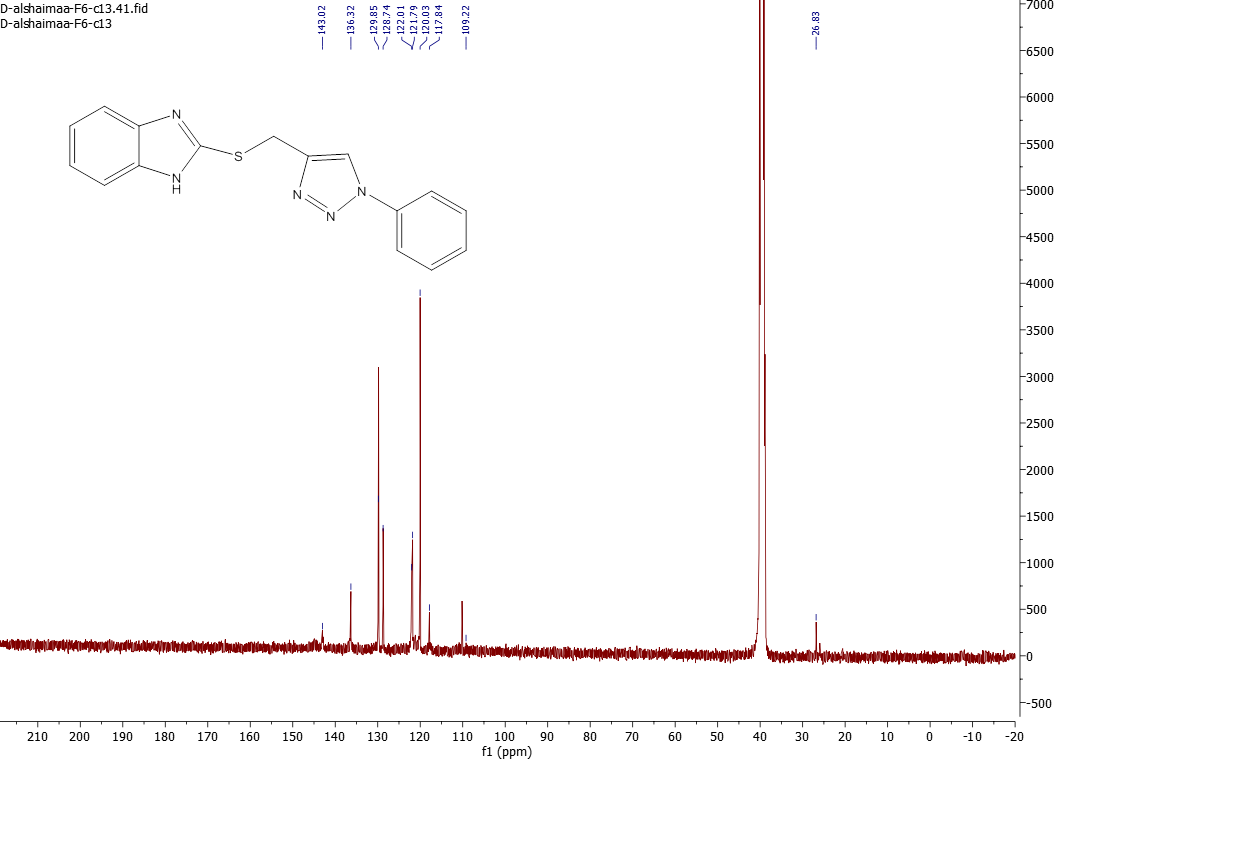
^13^C NMR (101 MHz, DMSO-*d*_6_) δ 143.0, 136.3 , 129.8, 128.7, 122.0, 121.8, 120.0, 117.8, 109.2, 26.8

**^1^H NMR spectrum of (6b):**


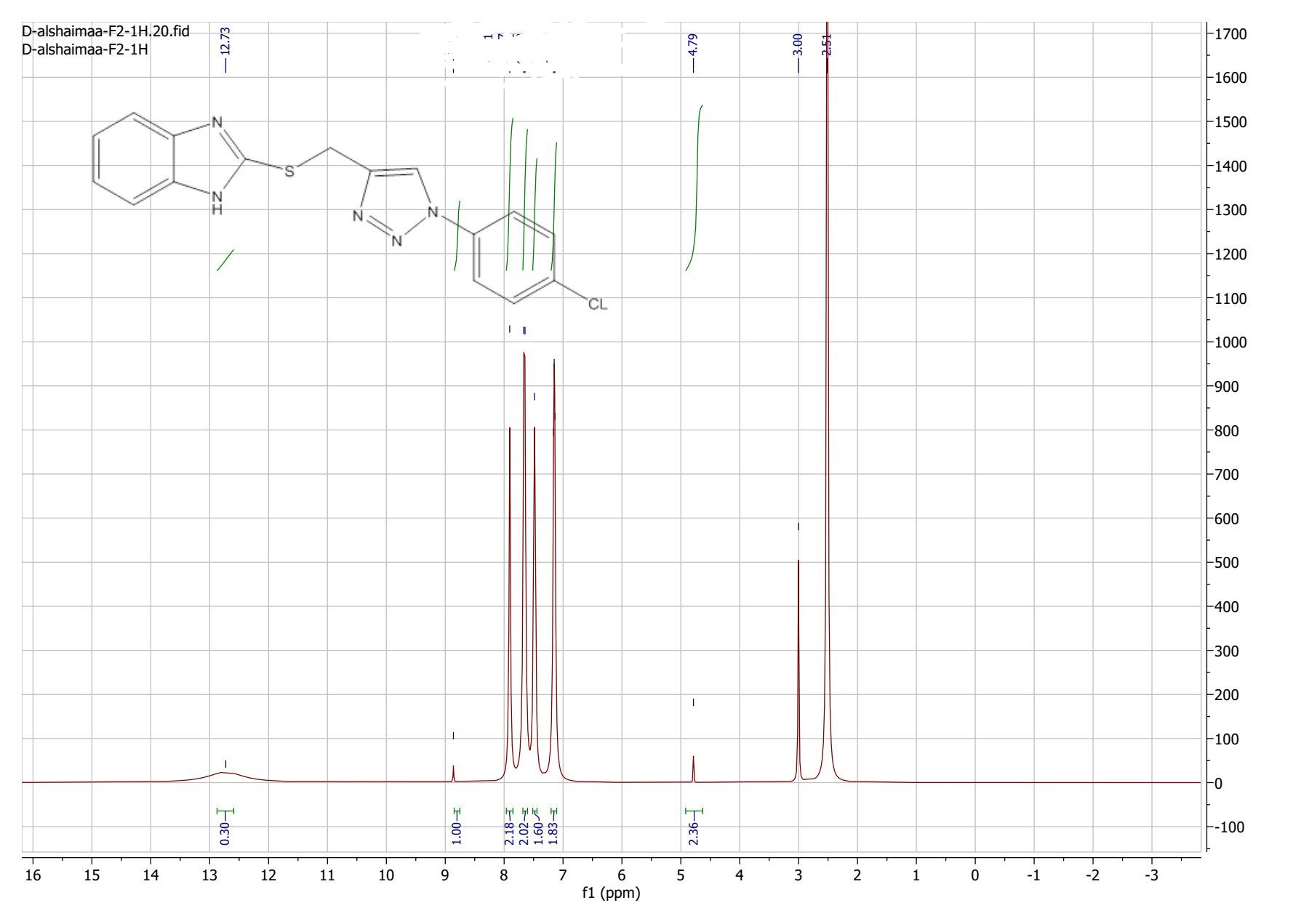


^1^H NMR (400 MHz, DMSO-*d*_6_): δ= 12.71 (br s, 1H, NH), 8.78 (s, 1H; triazole CH), 7.91 (d, *J* = 8.8 Hz, 2H, ArH-3',5'), 7.65 (d, *J* = 8.8 Hz, 2H, Ar-H-2',6'), 7.51 – 7.44 (m, 2H, ArH-4,7), 7.24 – 7.10 (m, 2H, ArH-5,6), 4.71 (s, 2H; SCH_2_)

**^13^C NMR spectrum of (6b):**


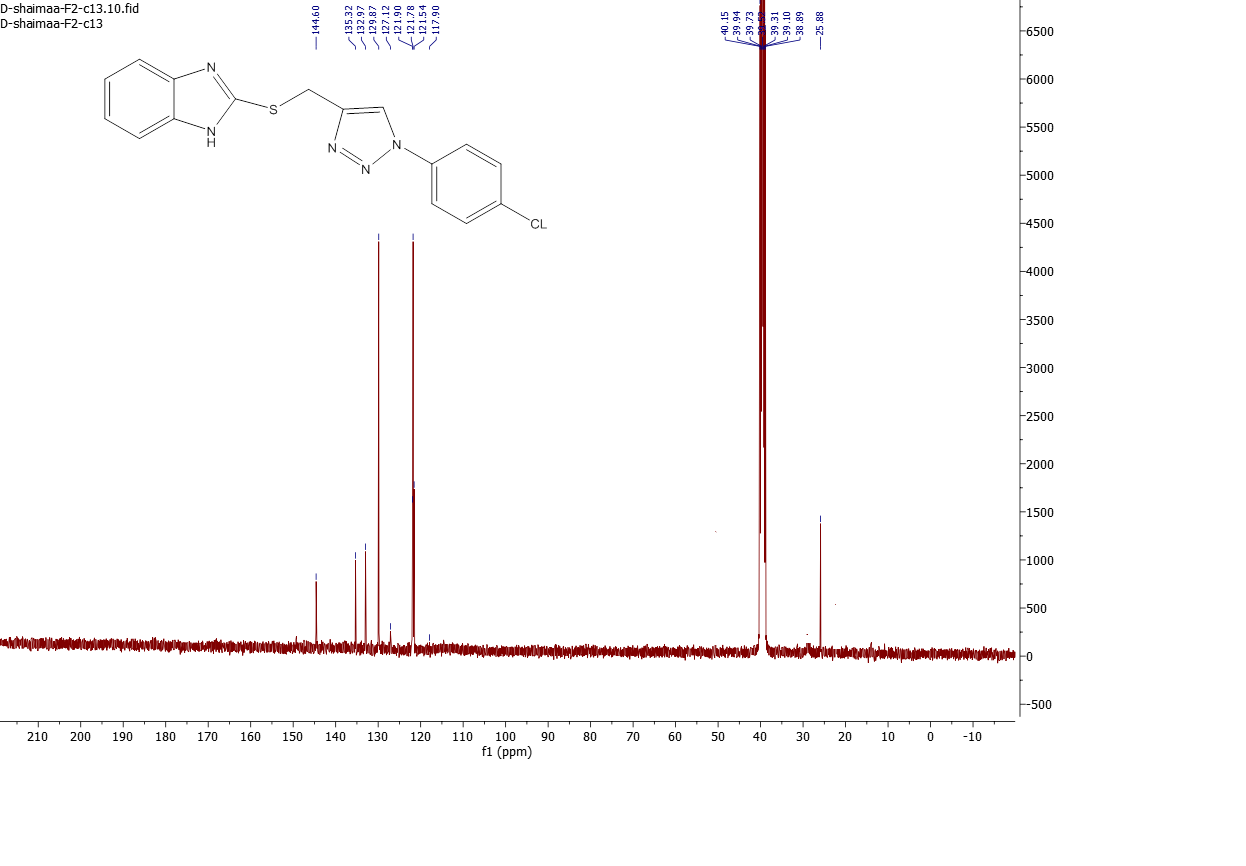


^13^C NMR (101 MHz, DMSO-*d*_6_) δ 144.6, 135.3, 132.9, 129.8, 127.1, 121.9, 121.7, 121.5, 117.9, 25.8

**^1^H NMR spectrum of (6c) :**


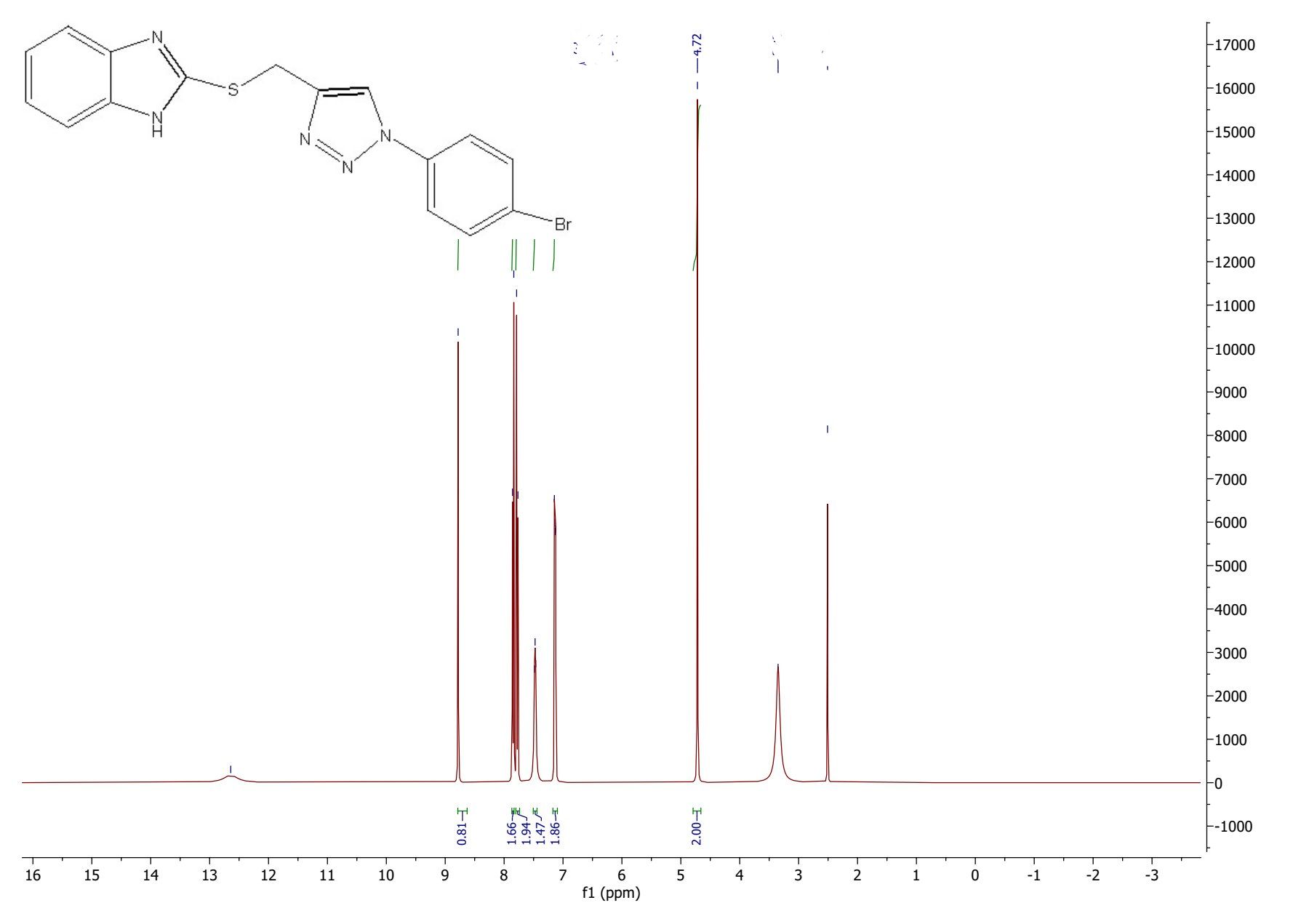


^1^H NMR (400 MHz, DMSO-*d*_6_): δ = 12.64 (br s, 1H, NH), 8.78 (s, 1H, triazole CH), 7.85 (d, *J* = 9.0 Hz, 2H, Ar-H -3',5'), 7.78 (d, *J* = 9.0 Hz, 2H, Ar-H-2',6'), 7.54 – 7.43 (m, 2H, Ar-H-4,7), 7.14 (dd , *J* = 6.0, 3.2 Hz, 2H, Ar-H-5,6), 4,72 (s, 2H, SCH_2_)

**^13^C NMR spectrum of (6c):**

**
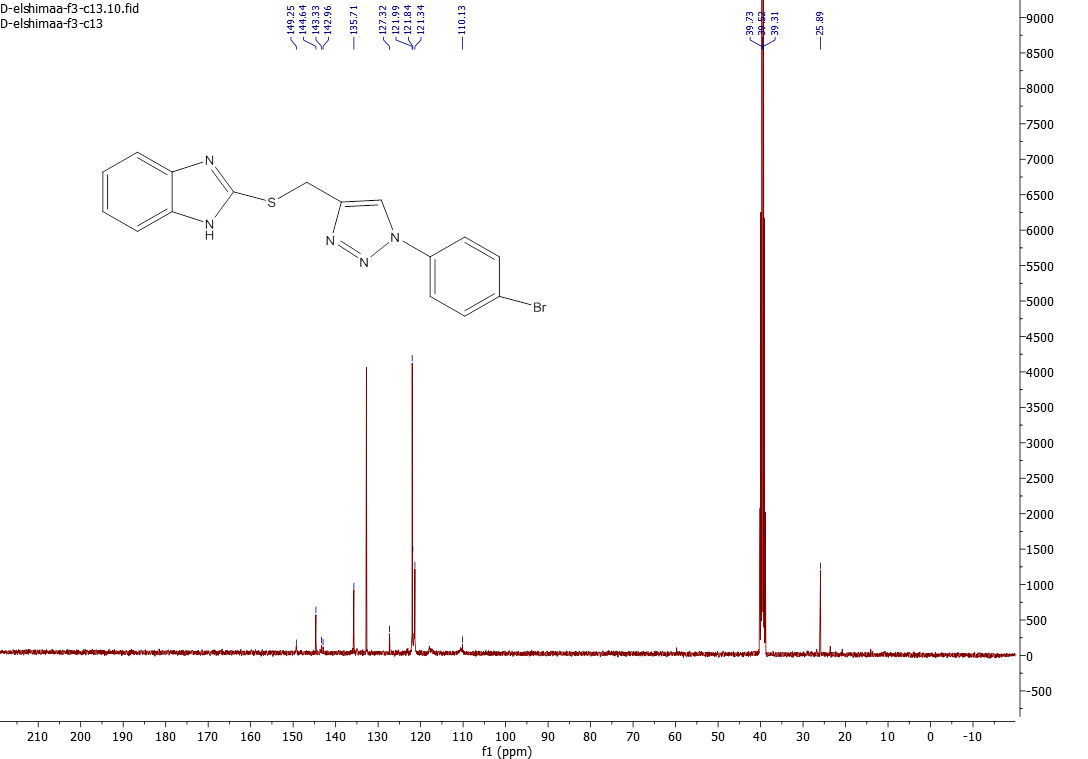
**

^13^C NMR (101 MHz, DMSO-*d*_6_) δ 149.2, 144.6, 143.3, 142.9, 135.7 ,127.3 ,121.9, 121.8 ,121.3 ,110.1 ,25.8

**^1^H NMR spectrum of (6d) :**

**
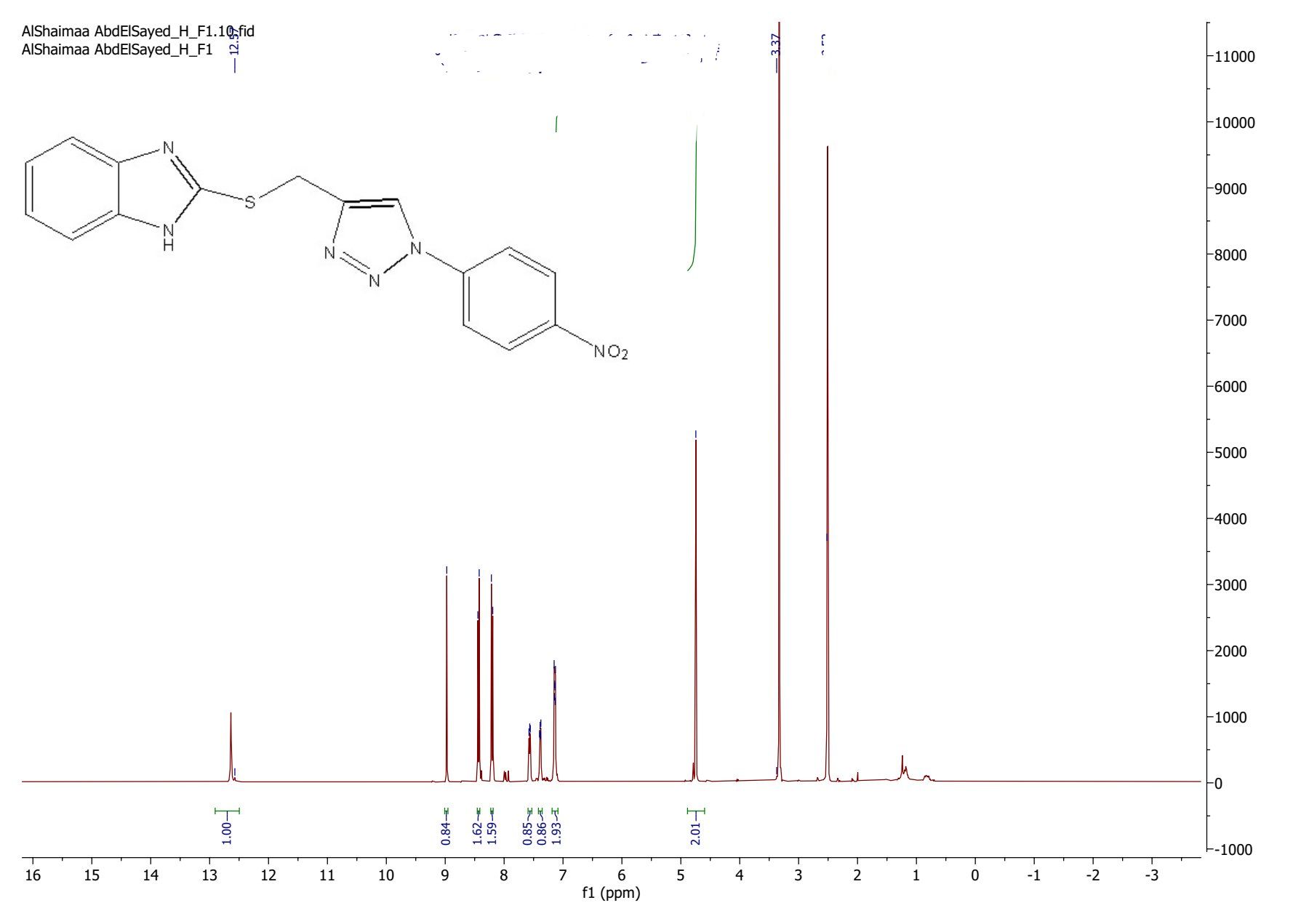
**

^1^H NMR (400 MHz, DMSO-*d_6_*): δ = 12.64 (br s, 1H, NH), 8.97 (s, 1H, triazole CH), 8.43 (d, *J* = 9.2 Hz, 2H, Ar-H-3',5'), 8.20 (d, *J* = 9.2 Hz, 2H, Ar-H-2',6'), 7.61 – 7.52 (m, 1H, Ar-H), 7.44 – 7.35 (m, 1H, Ar-H) , 7.21 – 7.08 (m, 2H, Ar-H-5,6), 4.74 (s, 2H, SCH_2_)

**^13^C NMR spectrum of (6d):**

**
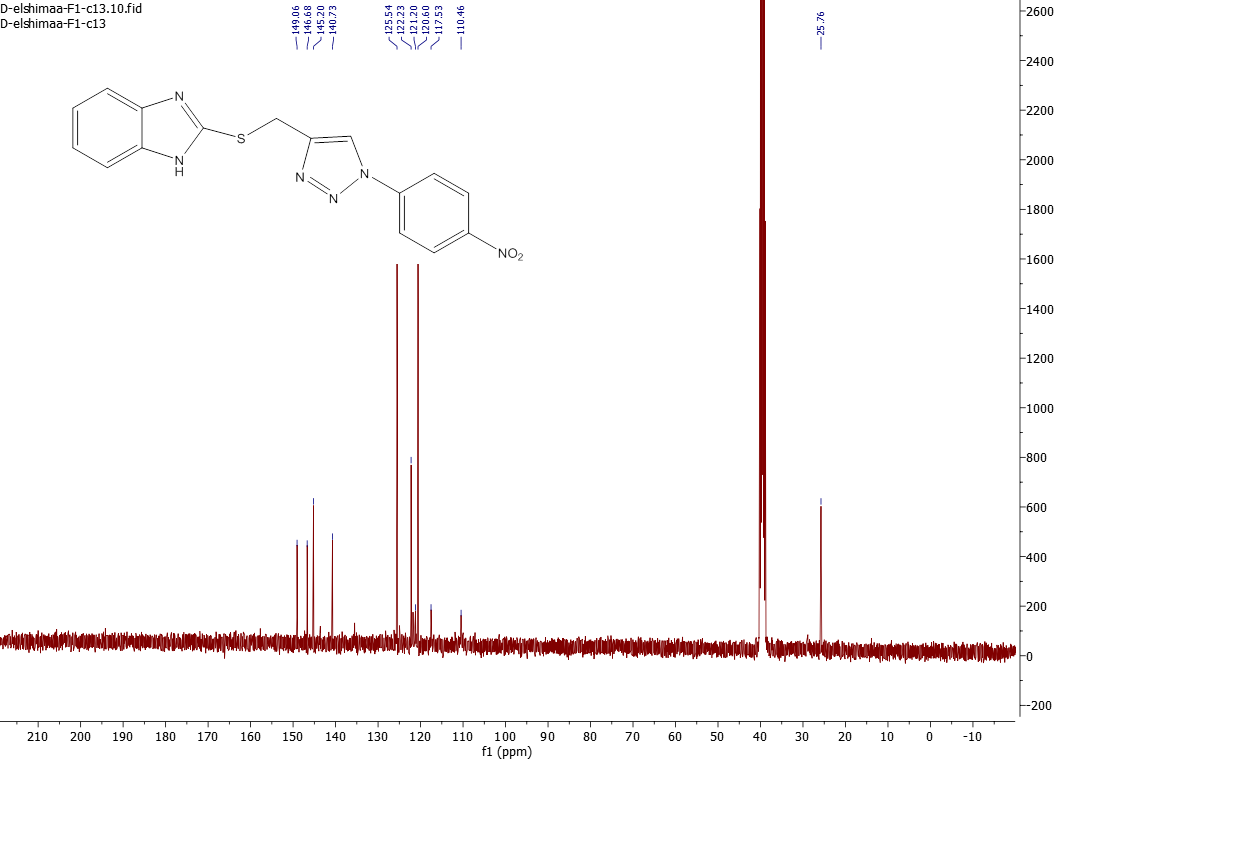
**

^13^C NMR (101 MHz, DMSO-*d*_6_) δ 149, 146.6, 145.2, 140.7, 125.5, 122.2, 121.2, 120.6, 117.5, 110.4, 25.7

IR spectrum of (6d):

**
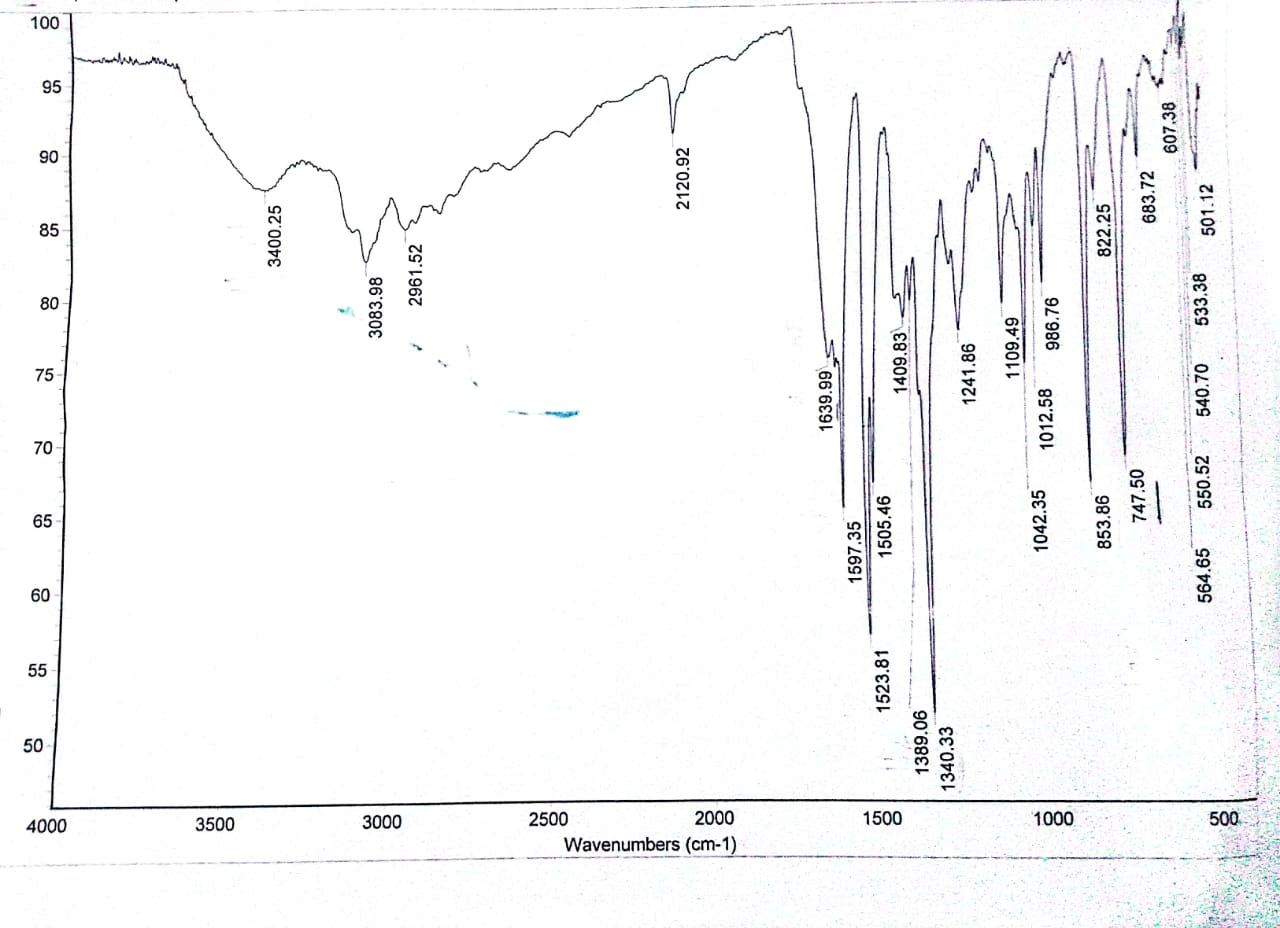
**

IR (KBr, ύ cm^-1^): 3400 (NH), 3084 (=CH), 2962 (CH_2_), 1640, 1598 (C=N, C=C), 1523, 1340 (NO_2_), 854 (*p*-bending).

**^1^H NMR spectrum of (6e) :**

**
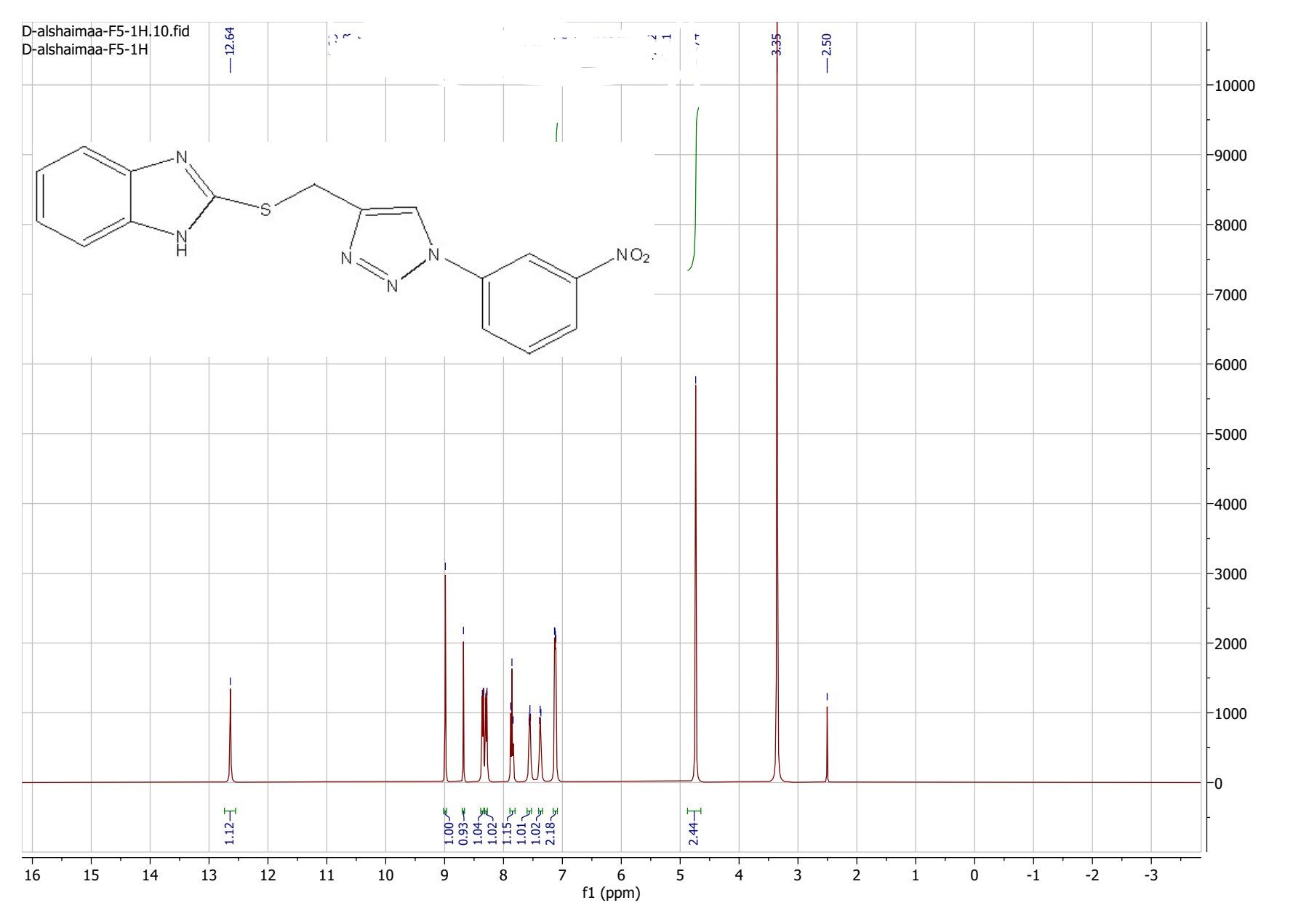
**

^1^H NMR (400 MHz, DMSO-*d*_6_): δ = 12.64 (br s, 1H, NH), 8.99 (s, 1H, triazole CH), 8.68 (s, 1H, Ar-H), 8.35 (dd , *J* = 8.2, 2.1 Hz, 1H, Ar-H), 8.29 (dd, *J* = 8.2, 2.2 Hz, 1H, Ar-H), 7.86 (t, *J* = 8.1 Hz, 1H, Ar-H), 7.66 – 7.50 (m, 1H, Ar-H), 7.43 – 7.32 (m, 1H, Ar-H), 7.12 (dd, *J* = 6.2, 3.0 Hz, 2H, Ar-H), 4.74 (s, 2H, SCH_2_)

**^13^C NMR spectrum of (6e):**

**
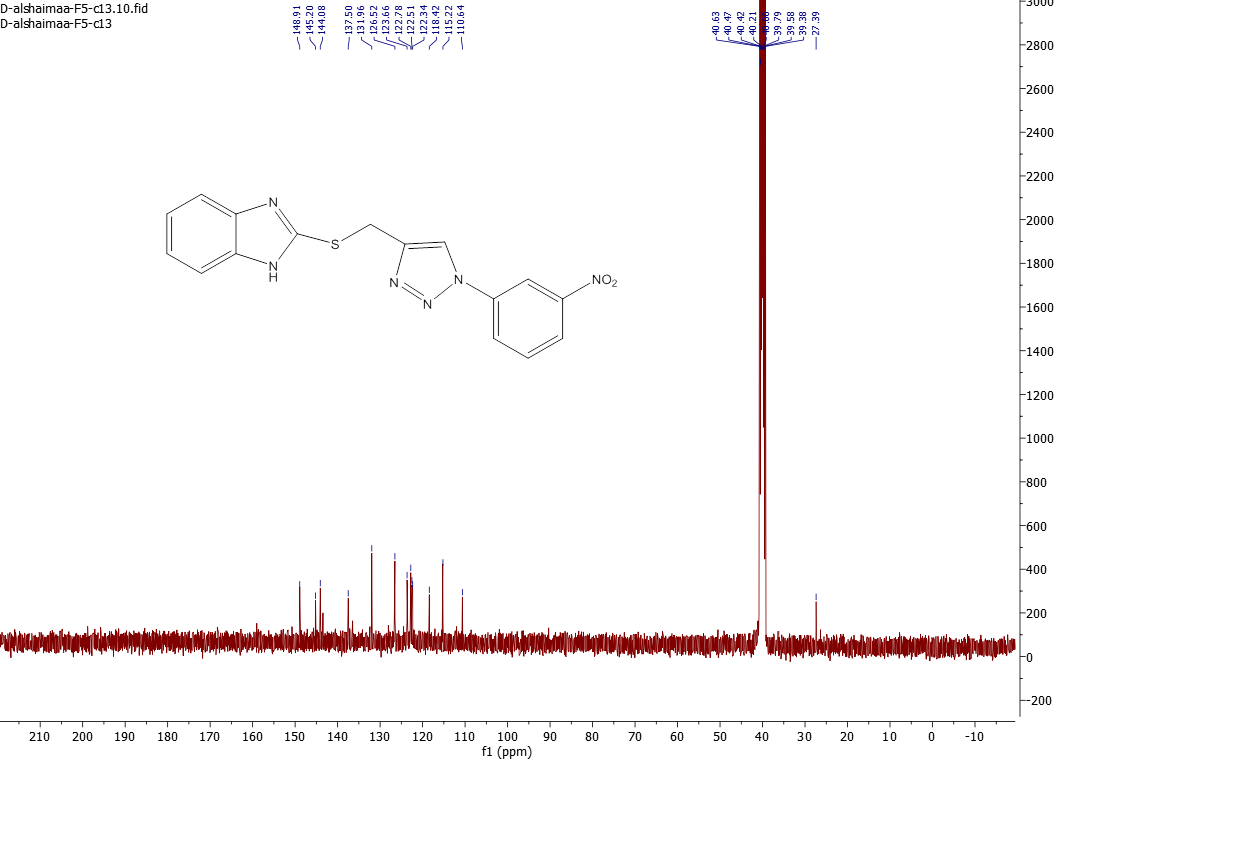
**

^13^C NMR (101 MHz, DMSO-*d*_6_): δ 148.9, 145.2, 144.0, 137.5, 131.9, 126.5, 123.6, 122.7, 122.3, 118.4, 115.2, 110.6, 27.3

**^1^H NMR spectrum of (6f) :**

**
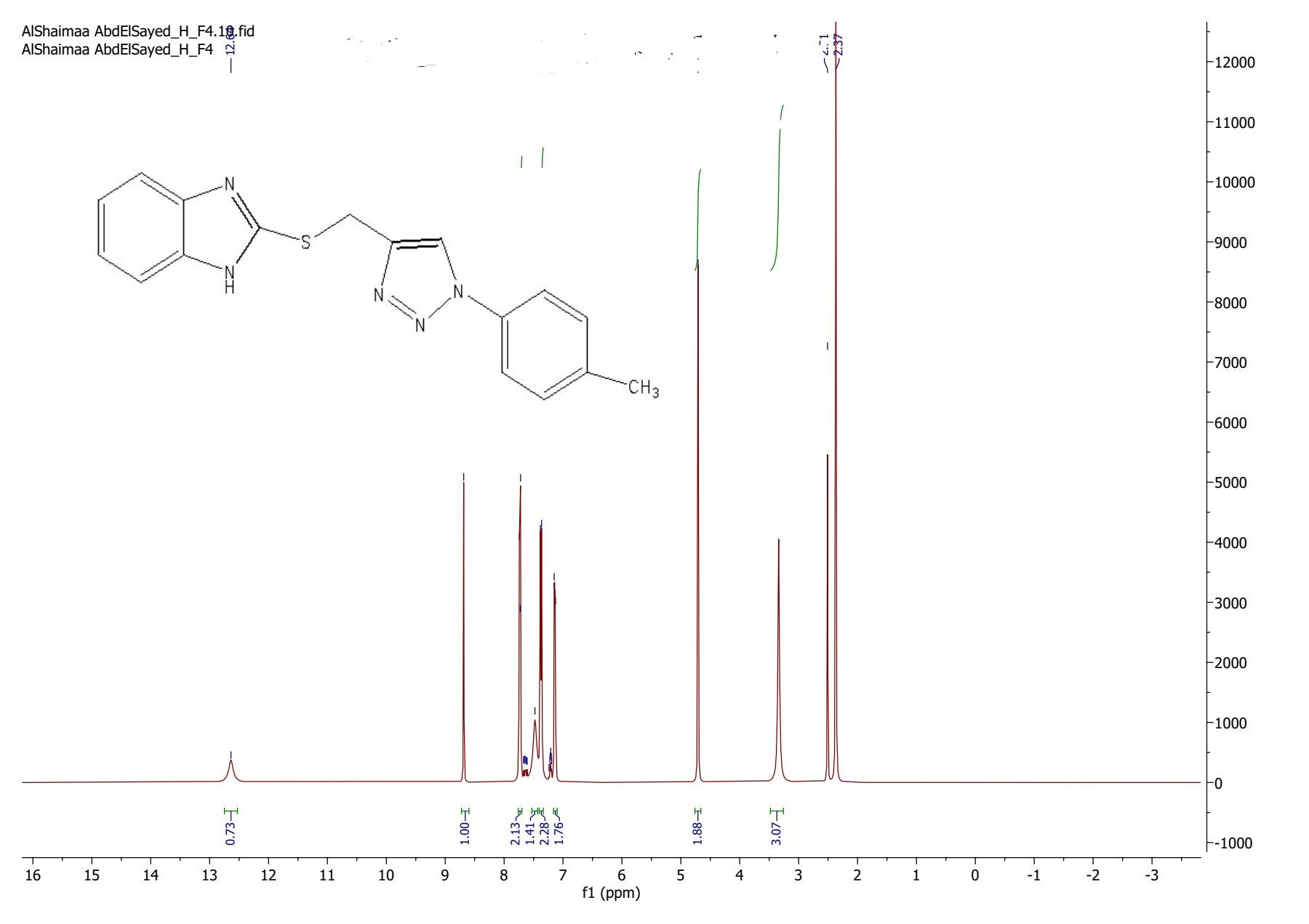
**

^1^H NMR (400 MHz, DMSO-*d*_6_): δ=12.64 (br s, 1H, NH), 8.69 (s, 1H, triazole CH), 7.73 (d, *J* = 8.3 Hz, 2H, Ar-H-3',5'), 7.48 (brs, 2H, Ar-H), 7.37 (d, *J* = 8.1 Hz, 2H, Ar-H), 7.18 – 7.08 (m, 2H, Ar-H-5,6), 4.71 (s, 2H, SCH_2_), 2.37 (s, 3H, CH_3_)

**^13^C NMR spectrum of (6f):**

**
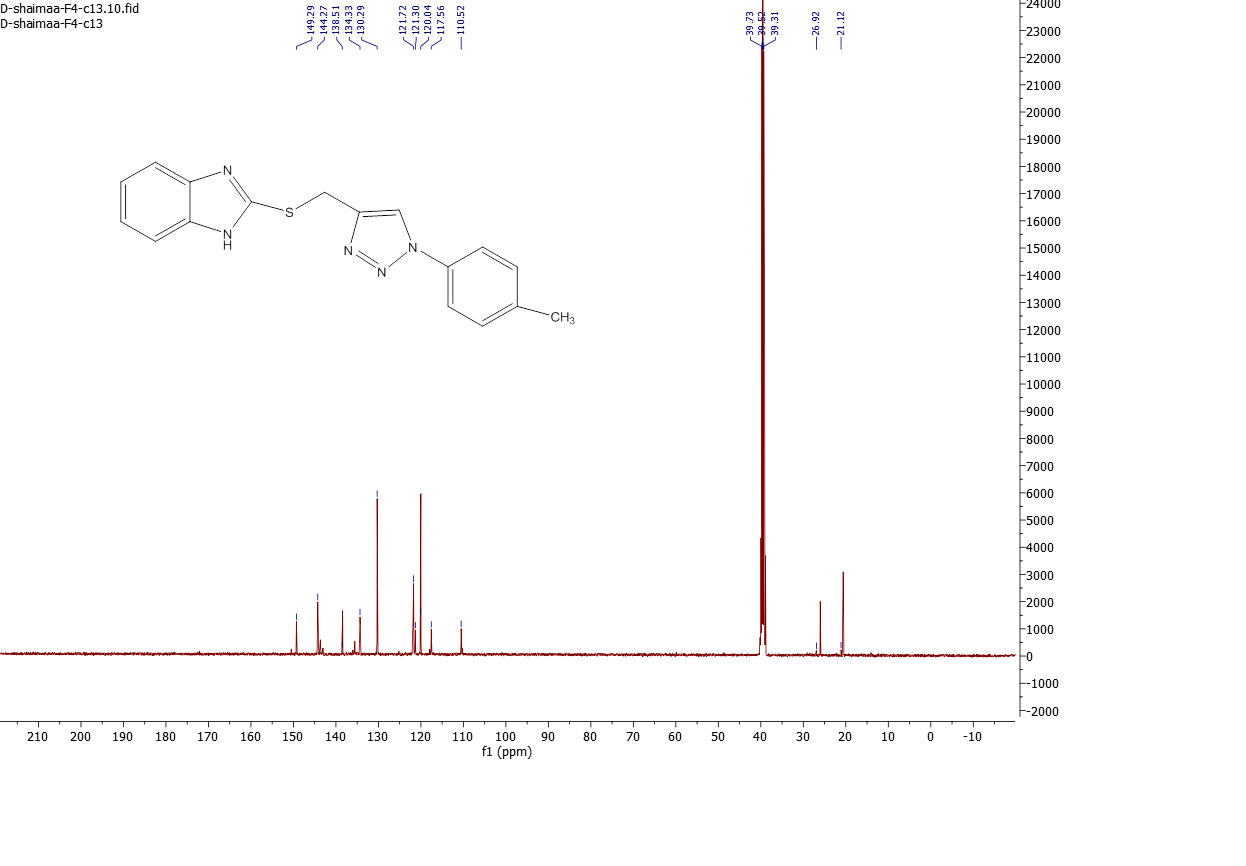
**

^13^C NMR (101 MHz, DMSO-*d*_6_) δ 149.2, 144.2, 138.5, 134.3, 130.3, 121.7, 121.3, 120.0, 117.5, 110.5, 26.9, 21.1

**^1^H NMR spectrum of (6g):**

**
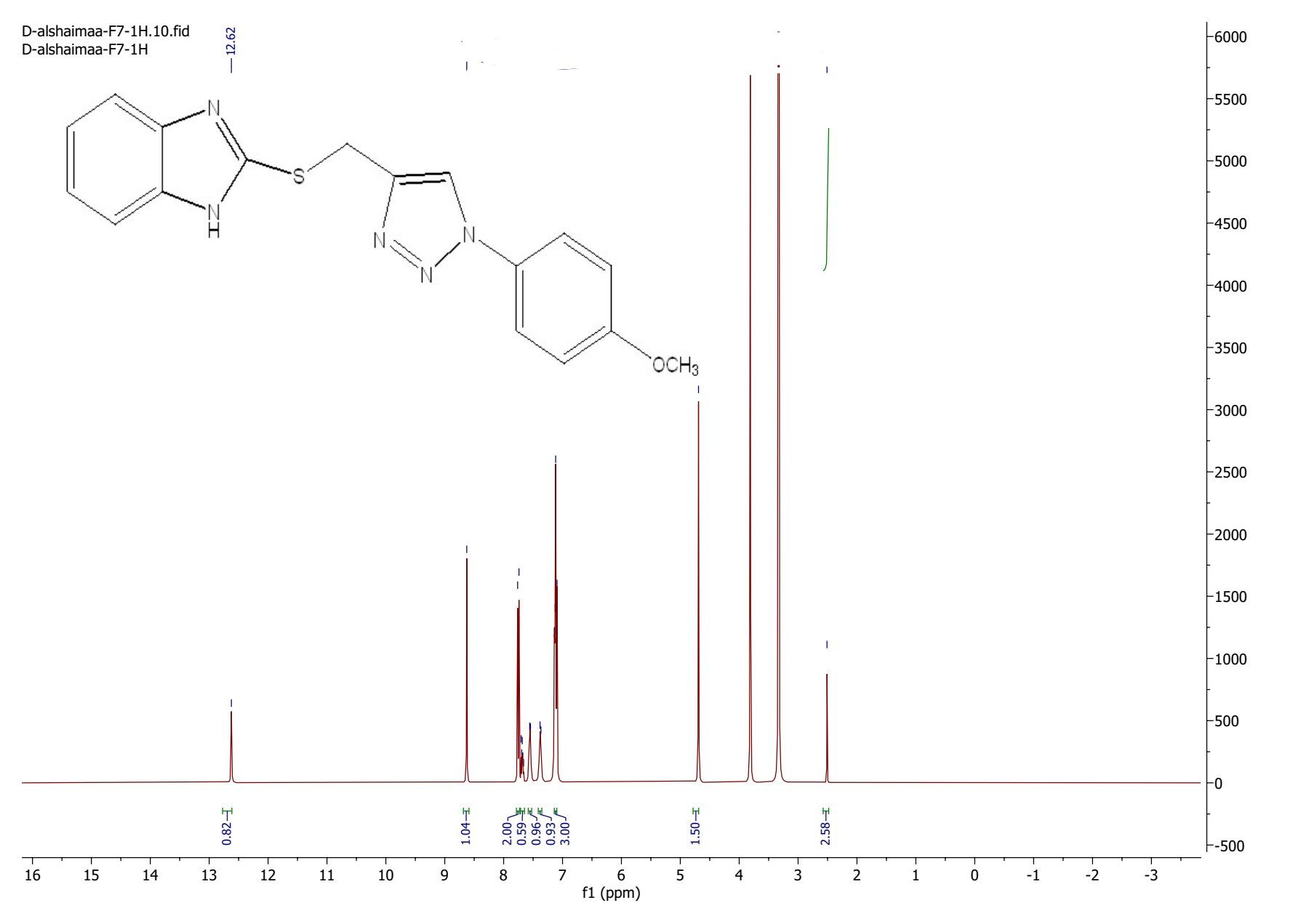
**

^1^H NMR (400 MHz, DMSO-*d*_6_): δ= 12.62 (brs, 1H, NH), 8.63 (s, 1H, triazole CH), 7.75 (d, *J* = 9.0 Hz, 2H, Ar-H-3',5'), 7.72 – 7.65 (m, 1H,Ar-H), 7.55 ( brs , 1H, Ar-H), 7.37 (bs, 1H,Ar-H), 7.18 – 7.01 (m, 3H, Ar-H) , 4.69 (s, 2H, SCH_2_), 2.51 (s, 3H, OCH_3_)

**^13^C NMR spectrum of (6g):**

**
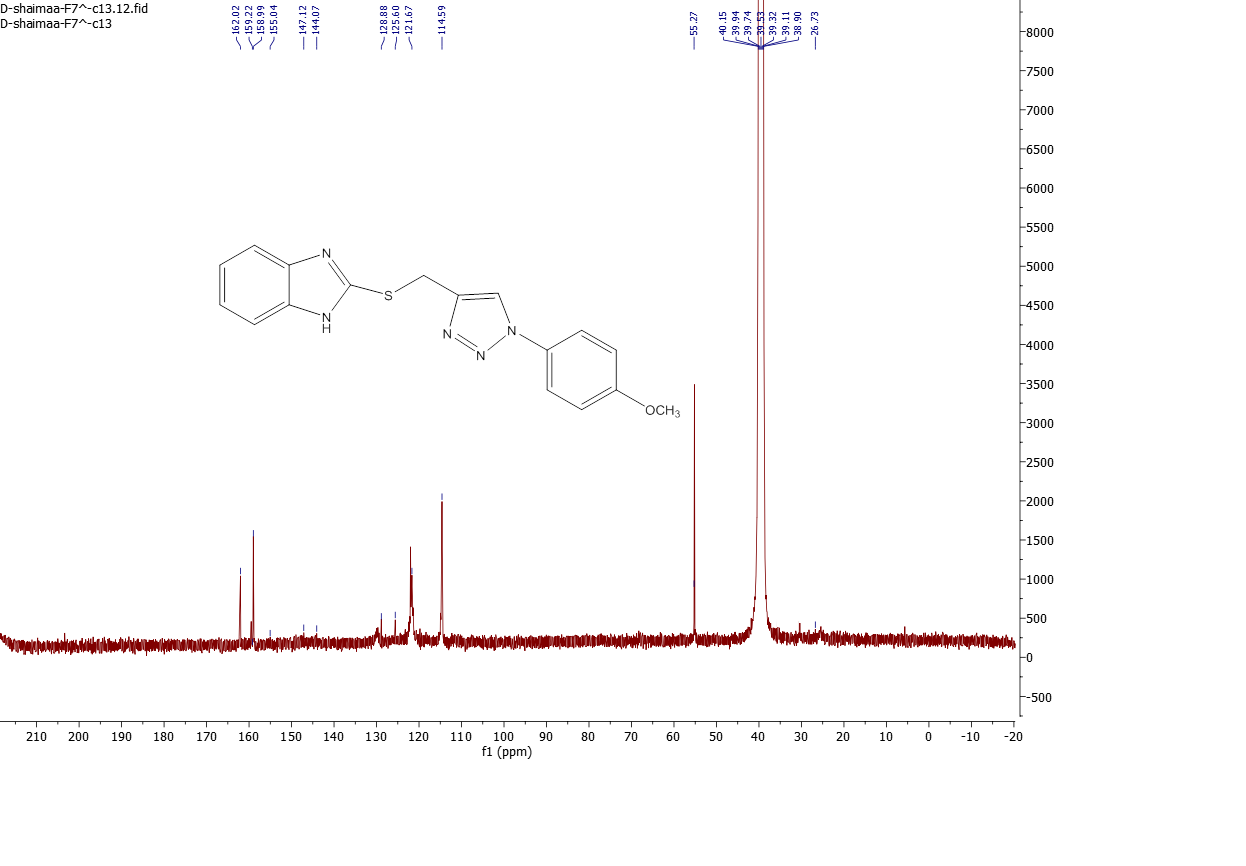
**

^13^C NMR (101 MHz, DMSO-*d*_6_) δ 162, 159.2, 158.9, 155.0, 147.1, 144.0, 128.8, 125.6, 121.6, 114.6, 55.2, 26.7.

**^1^H NMR spectrum of (6h):**

**
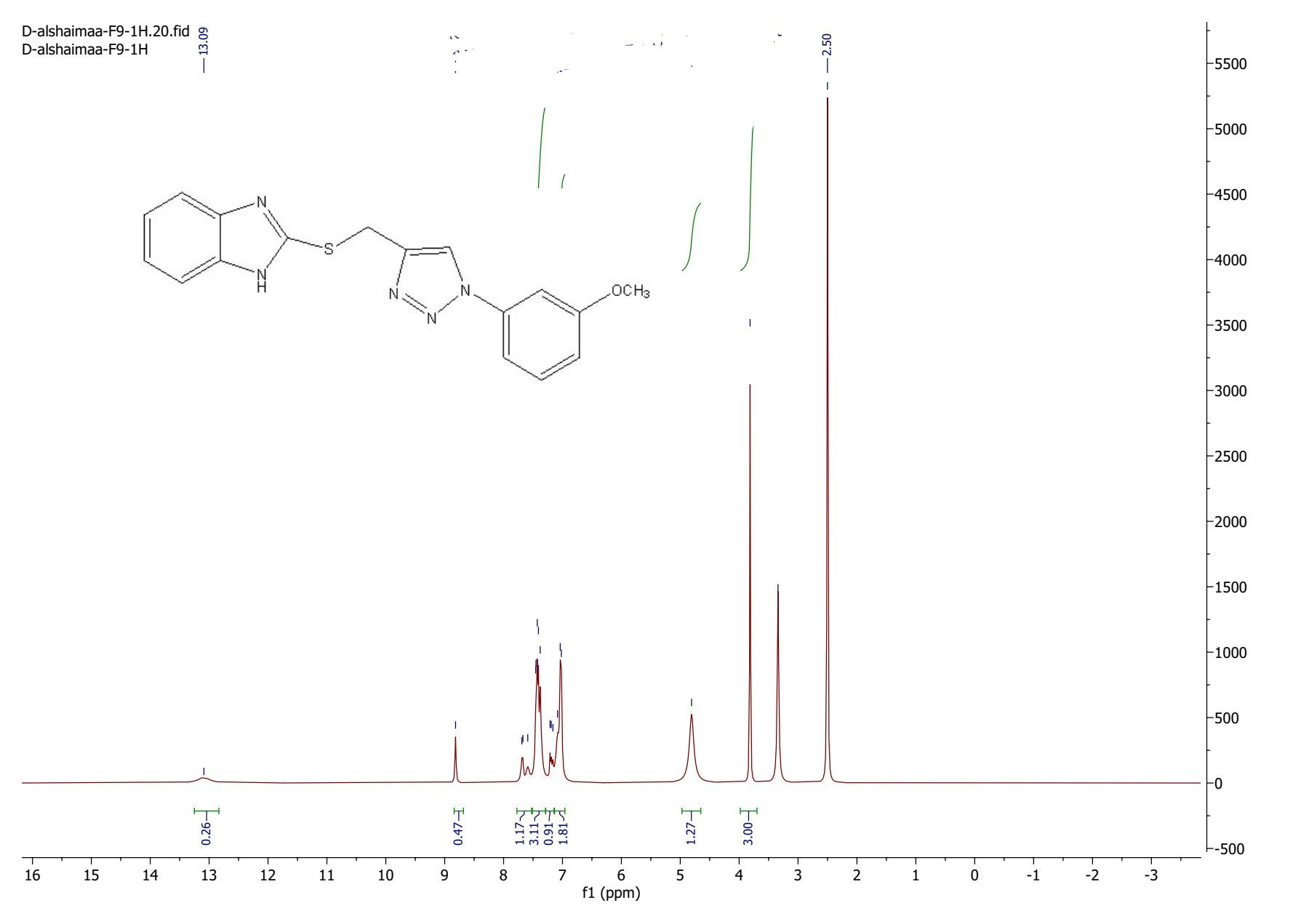
**

^1^H NMR (400 MHz, DMSO-*d*_6_): δ = 13.09 (br s,1H, NH), 8.82 (s, 1H, triazole CH), 7.68 (d, *J* = 7.4 Hz, 1H,Ar-H), 7.49 – 7.27 (m, 4H,Ar-H), 7.23 – 7.13 (m, 1H,Ar-H), 7.10 – 7.00 (m, 2H,Ar-H), 4.81 (s, 2H, SCH_2_), 3.82 (s, 3H, OCH_3_)

**^13^C NMR spectrum of (6h):**

**
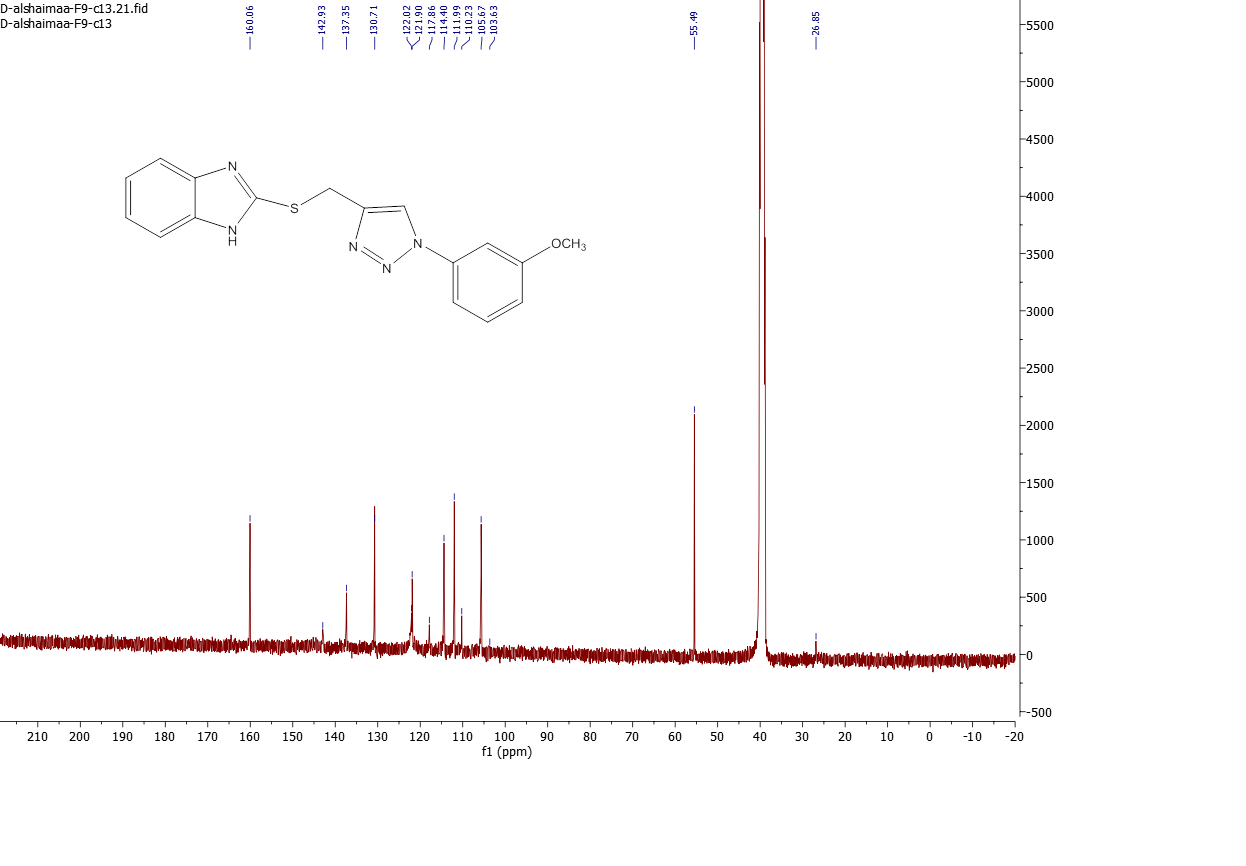
**

^13^C NMR (101 MHz, DMSO-*d*_6_) δ 160.0, 142.9, 137.3, 130.7, 122.0, 121.9, 117.8, 114.4, 111.9, 110.2, 105.6, 103.6, 55.5, 26.8

**^1^H NMR spectrum of (6i) :**

**
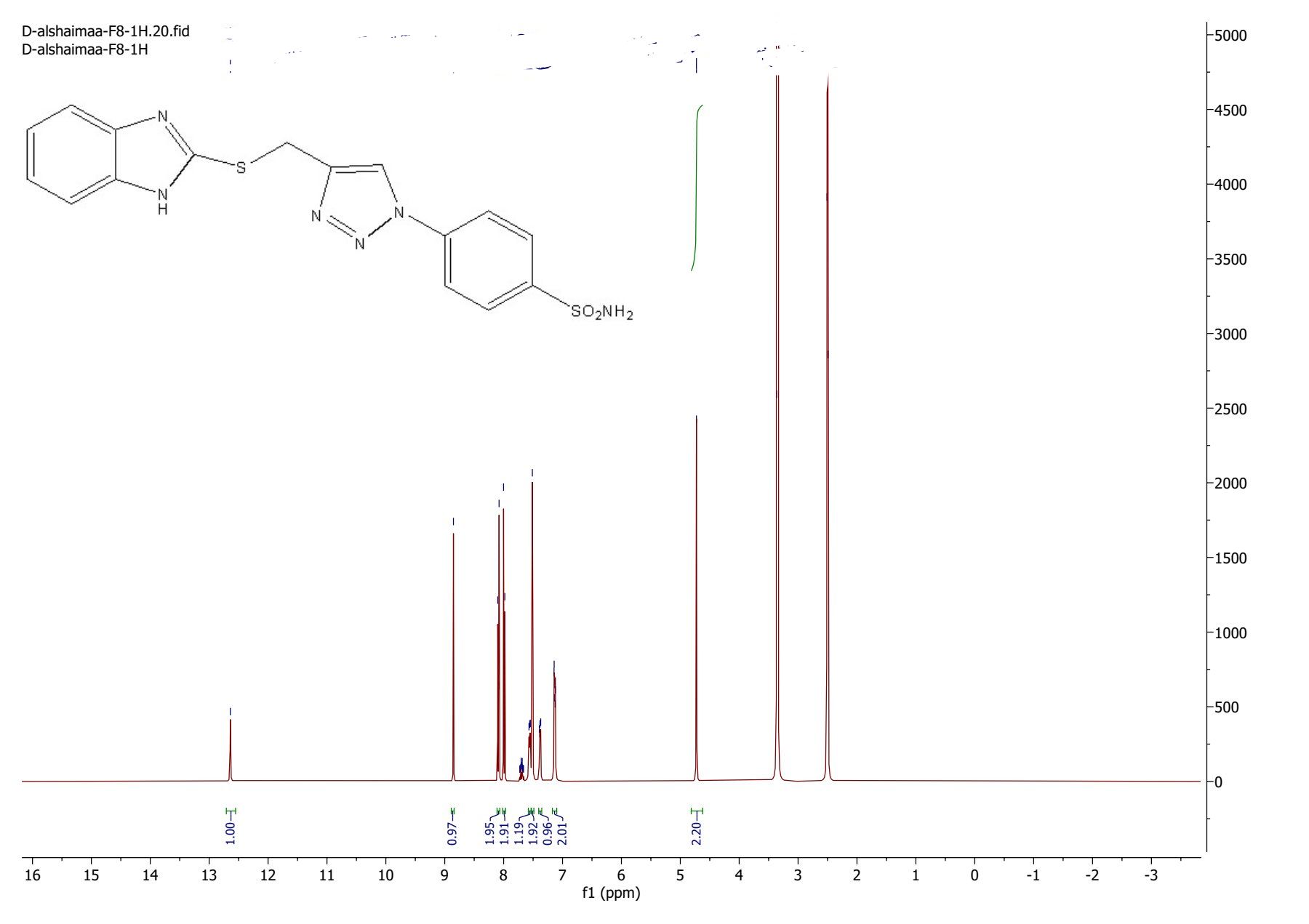
**

^1^H NMR (400 MHz, DMSO-*d*_6_): δ=12.64 (brs, 1H, NH), 8.85 (s, 1H, triazole CH) ,8.09 (d, *J* = 8.4 Hz, 2H, Ar-H-3',5'), 7.99 (d, *J* = 8.4 Hz, 2H; Ar-H-2',6'), 7.62 – 7.53 (m, 1H, Ar-H), 7.51 (s, 2H, NH_2_), 7.42 – 7.34 (m, 1H, Ar-H), 7.18 – 7.09 (m, 2H, Ar-H-5,6), 4.73 (s, 2H, SCH_2_)

**^13^C NMR spectrum of (6i):**

**
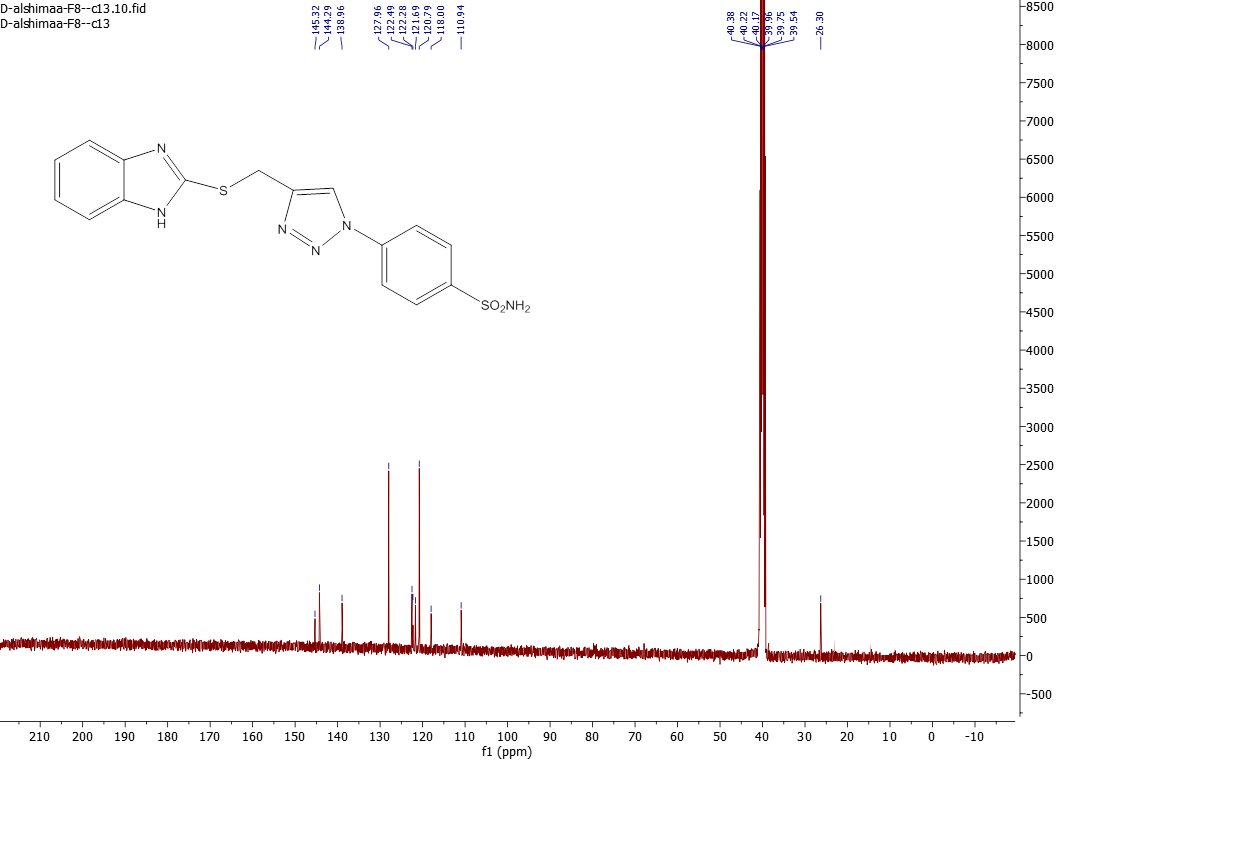
**

^13^C NMR (101 MHz, DMSO) δ 145.3, 144.3, 138.9, 127.9, 122.5, 122.2, 121.6, 120.8, 118, 110.9, 26.3

**^1^H NMR spectrum of (10a):**

**
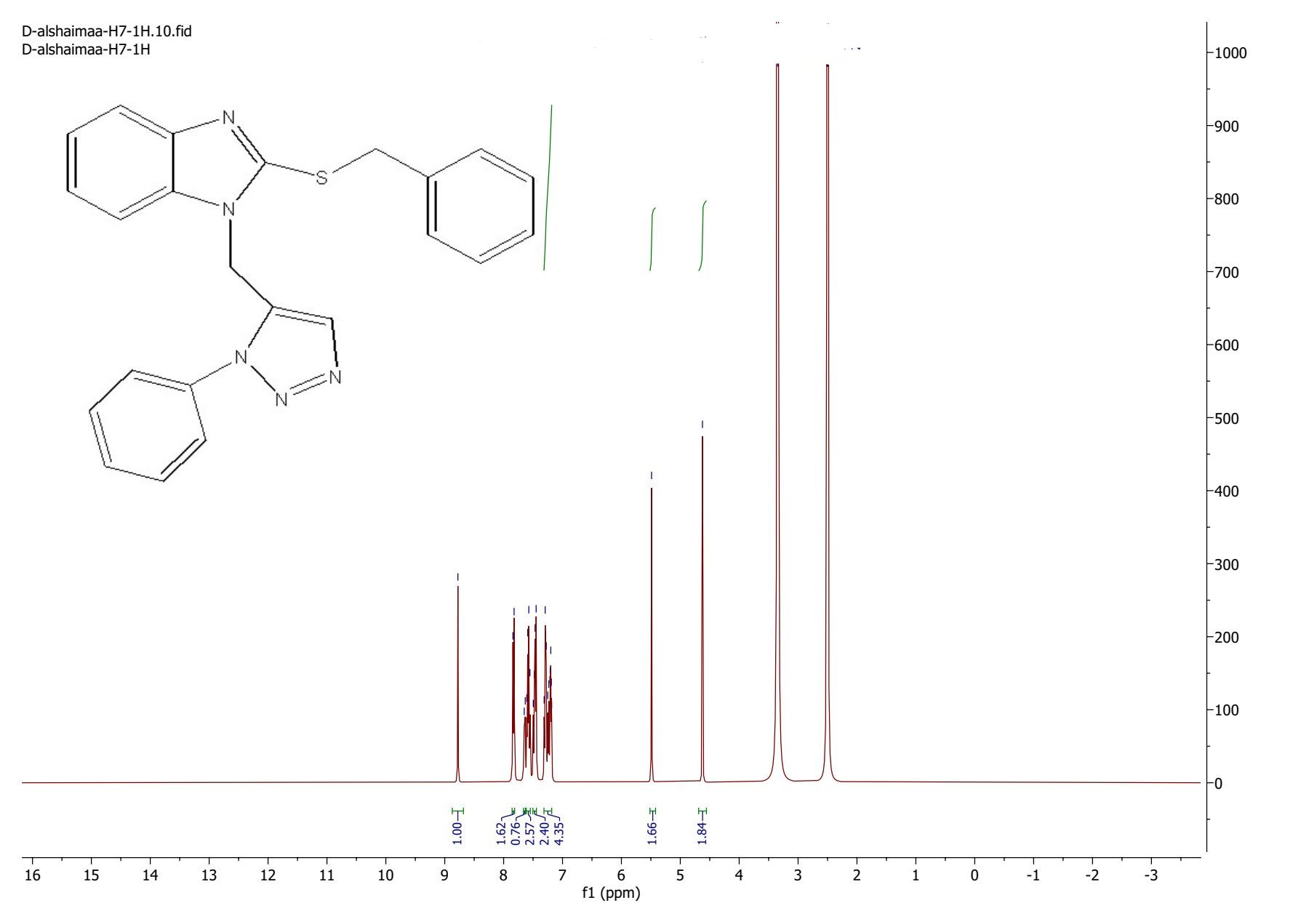
**

^1^H NMR (400 MHz, DMSO-*d*_6_): δ = 8.78 (s, 1H, triazole CH), 7.83 (d, *J* = 7.7 Hz, 2H, ArH), 7.64 (d, *J* = 7.0 Hz, 1H, Ar-H), 7.62 – 7.54 (m, 3H, Ar-H), 7.52 – 7.41 (m, 3H, Ar-H), 7.36 – 7.13 (m, 5H, Ar-H), 5.49 (s, 2H, N-CH_2_(, 4.62 (s, 2H, S-CH_2_)

**^13^C NMR spectrum of (10a):**

**
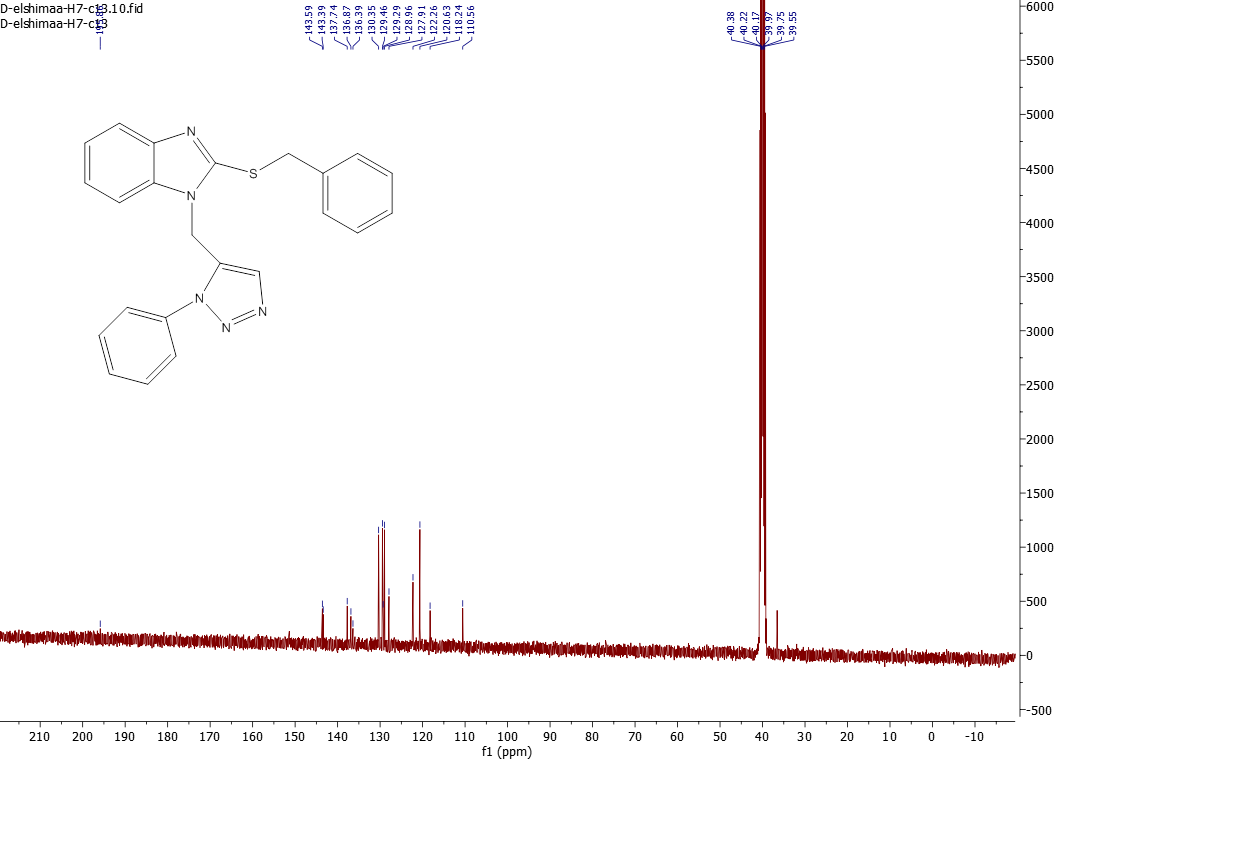
**

^13^C NMR (101 MHz, DMSO-*d*_6_) δ 143.5, 143.3, 137.7, 136.8, 136.3, 130.3 , 129.4, 129.2, 128.9, 127.9, 122.2, 120.6, 118.2, 110.5, 39.1, 36.5

**^1^H NMR spectrum of (10b):**

**
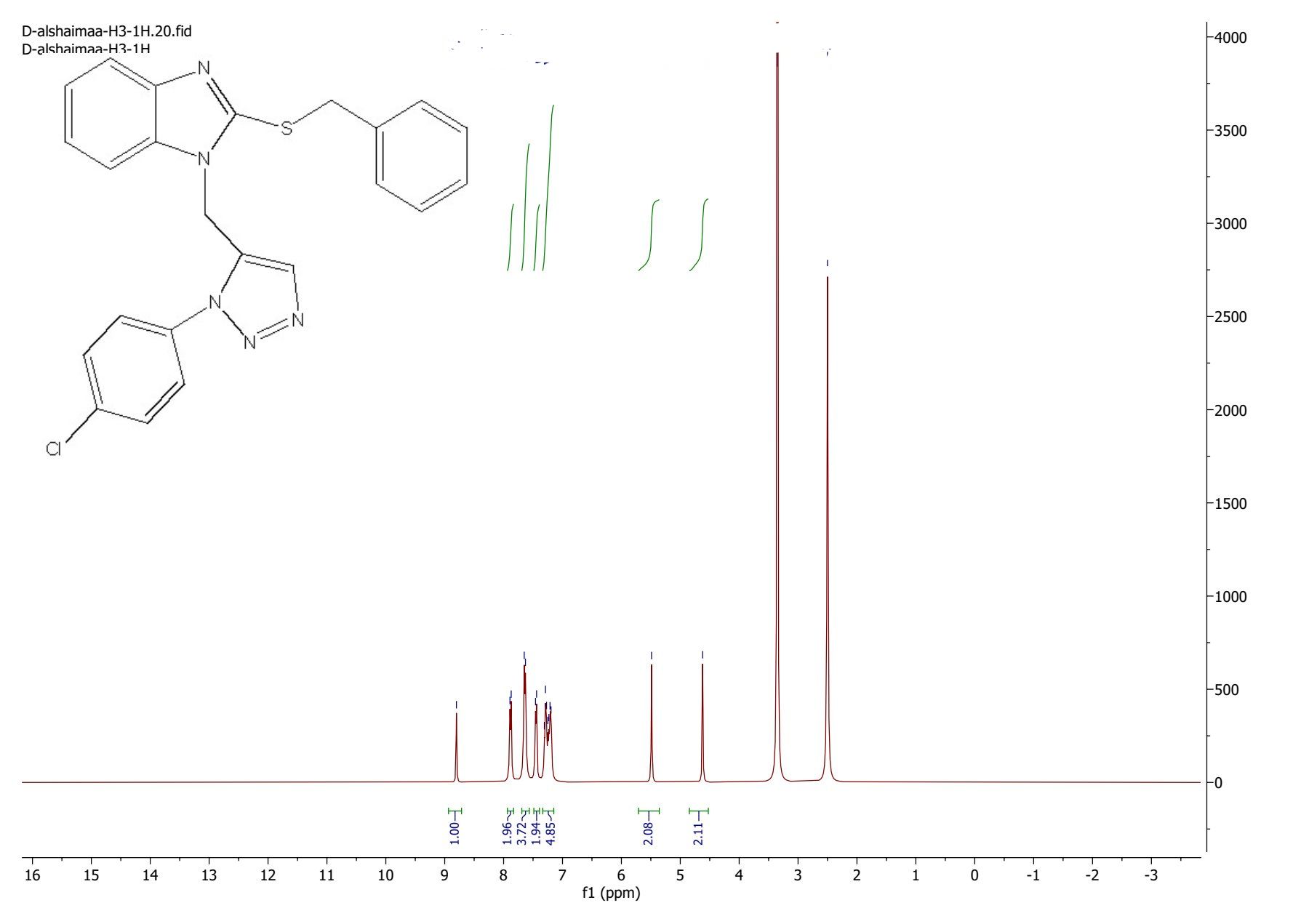
**

^1^H NMR (400 MHz, DMSO-*d_6_*): δ= 8.80 (s, 1H, triazole CH), 7.88 (d, *J* = 8.4 Hz, 2H, Ar-H-4,7 ), 7.64 (d, *J* = 8.5 Hz, 4H, Ar-H), 7.45 (d, *J* = 7.3 Hz, 2H, Ar-H) , 7.33 – 7.18 (m, 5H, Ar-H), 5.49 (s, 2H, N-CH_2_(, 4.62 (s, 2H, S-CH_2_)

**^13^C NMR spectrum of (10b):**

**
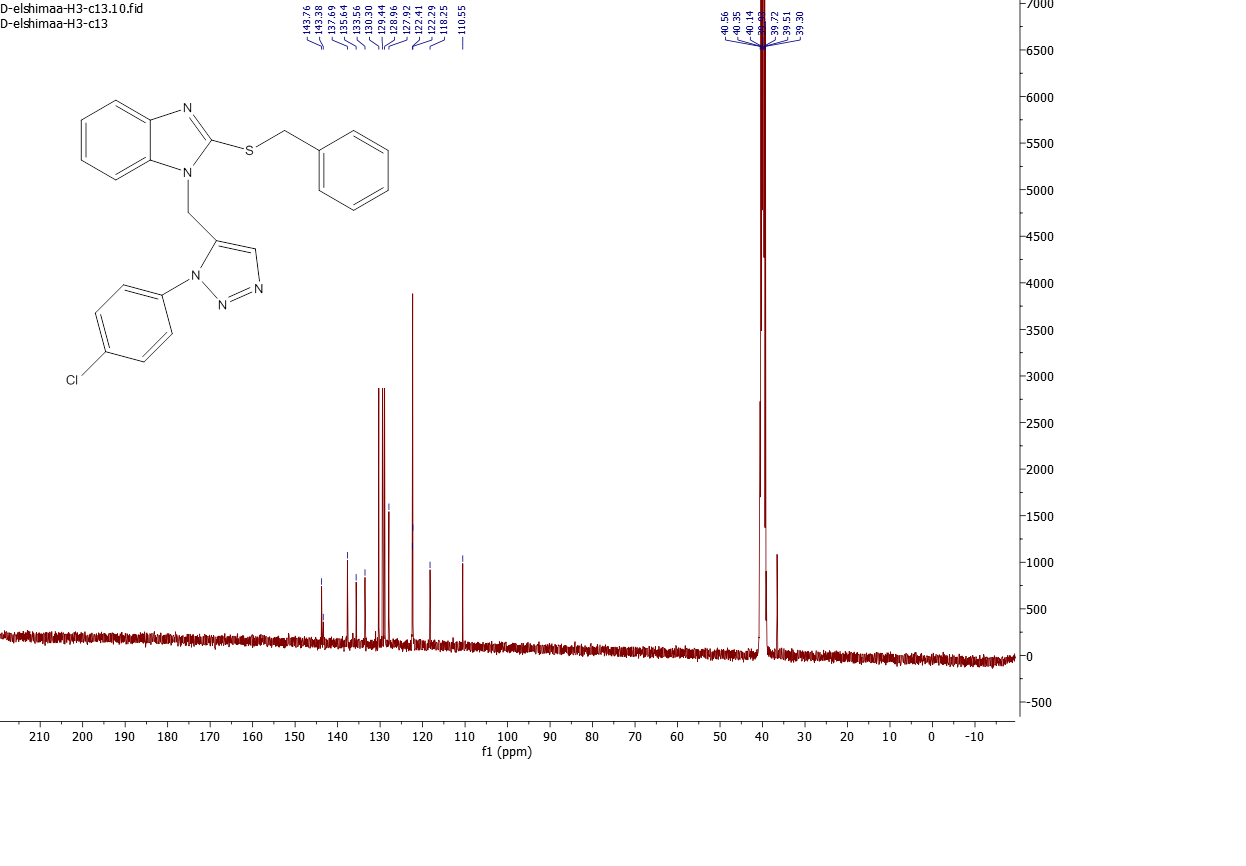
**

^13^C NMR (101 MHz, DMSO-*d*_6_) δ 143.7, 143.3, 137.6, 135.6, 133.5, 130.3, 129.4, 128.9, 127.9, 122.4, 122.2, 118.2, 110.5, 39.1, 36.5

**^1^H NMR spectrum of (10c):**

**
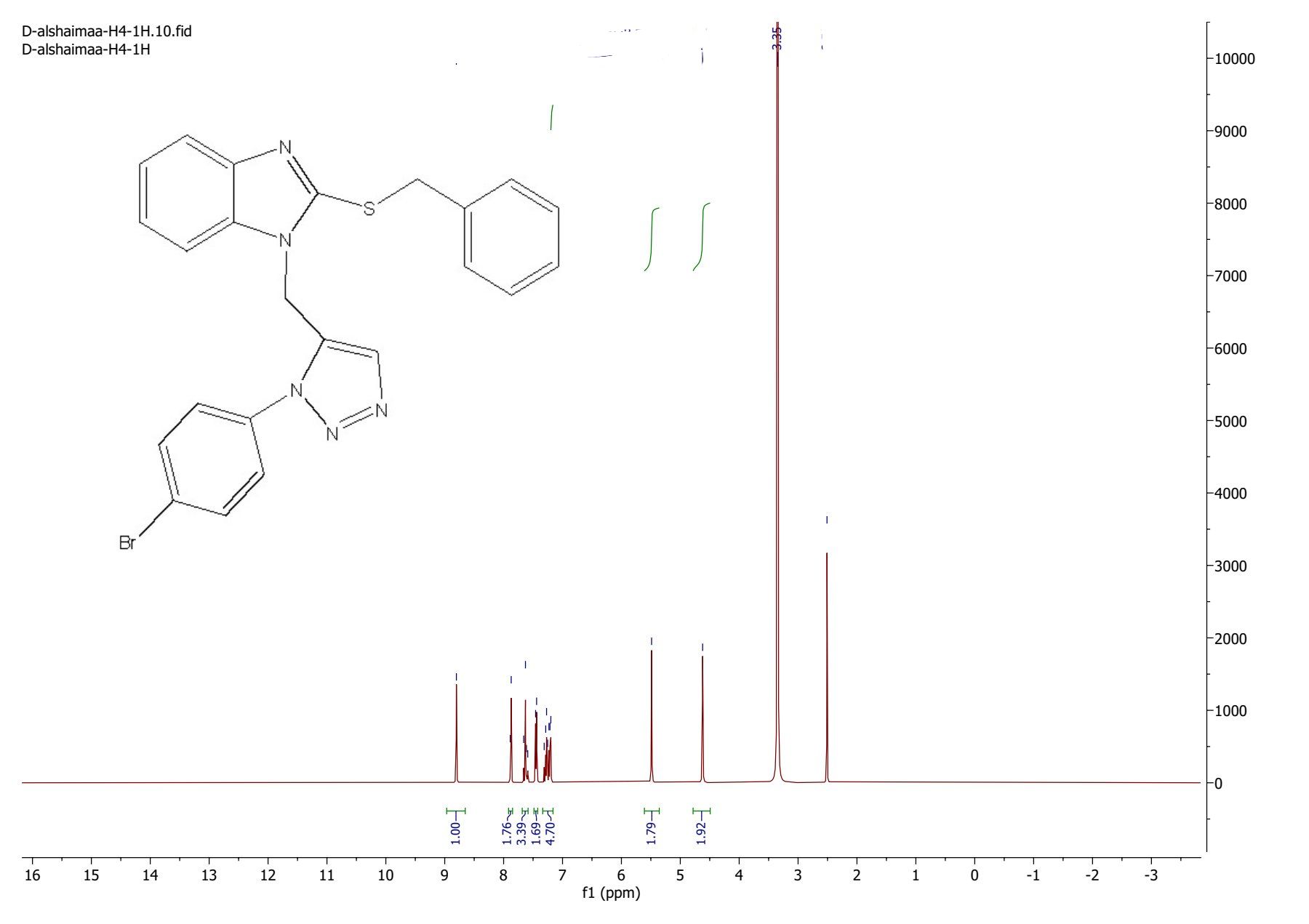
**

^1^H NMR (400 MHz, DMSO-*d_6_*): δ = 8.80 (s, 1H, triazole CH), 7.87 (d, *J* = 8.4 Hz, 2H, Ar-H-3 ̏,5 ̏ ), 7.68 – 7.57 (m, 4H, Ar-H), 7.45 (d, *J* = 7.3 Hz, 2H, Ar-H ), 7.40 – 7.14 (m, 5H, Ar-H), 5.49 (s, 2H, N-CH_2_(, 4.62 (s, 2H, S-CH_2_).

**^13^C NMR spectrum of (10c):**

**
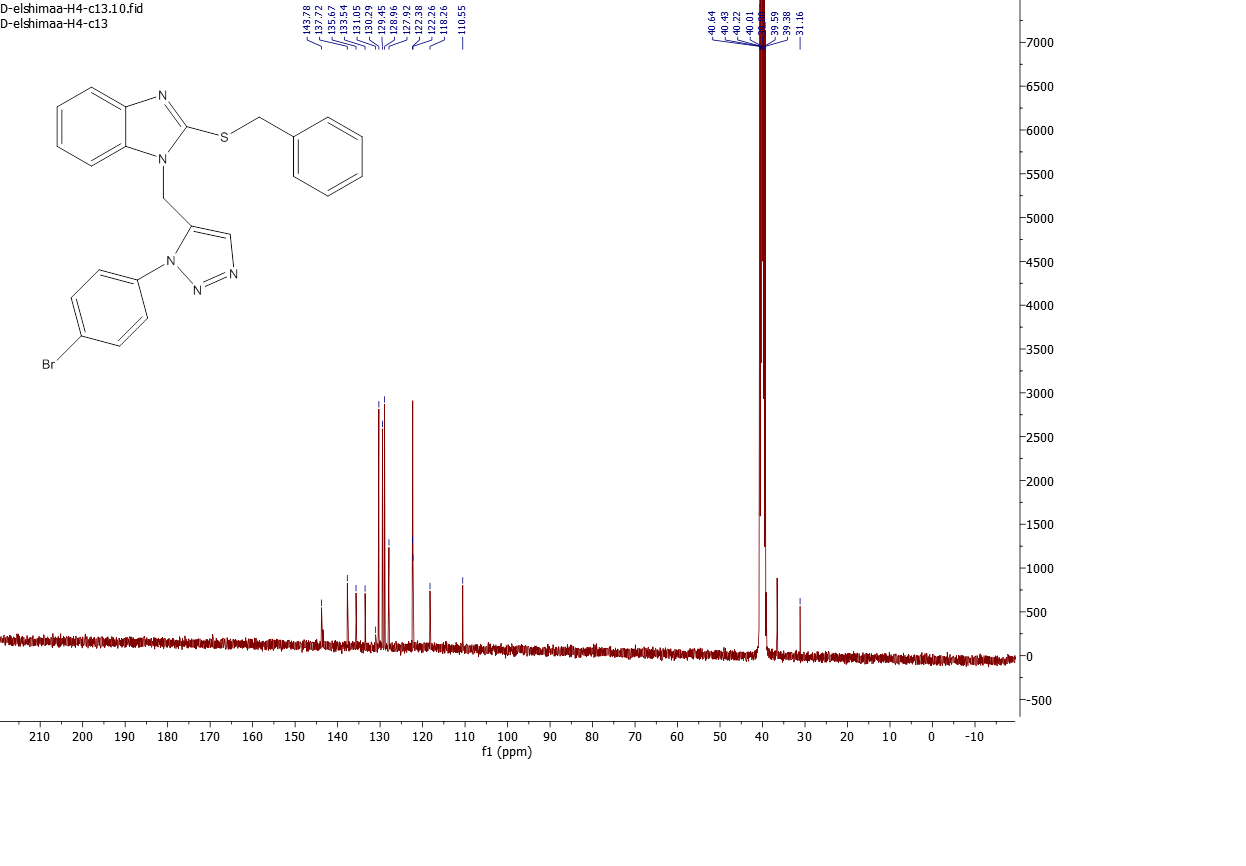
**

^13^C NMR (101 MHz, DMSO-*d*_6_) δ 143.7, 137.7, 135.6, 133.5, 131, 130.2, 129.4, 128.9, 127.9, 122.3, 122.2, 118.2, 110.5, 39.3, 31.1

**^1^H NMR spectrum of (10d):**

**
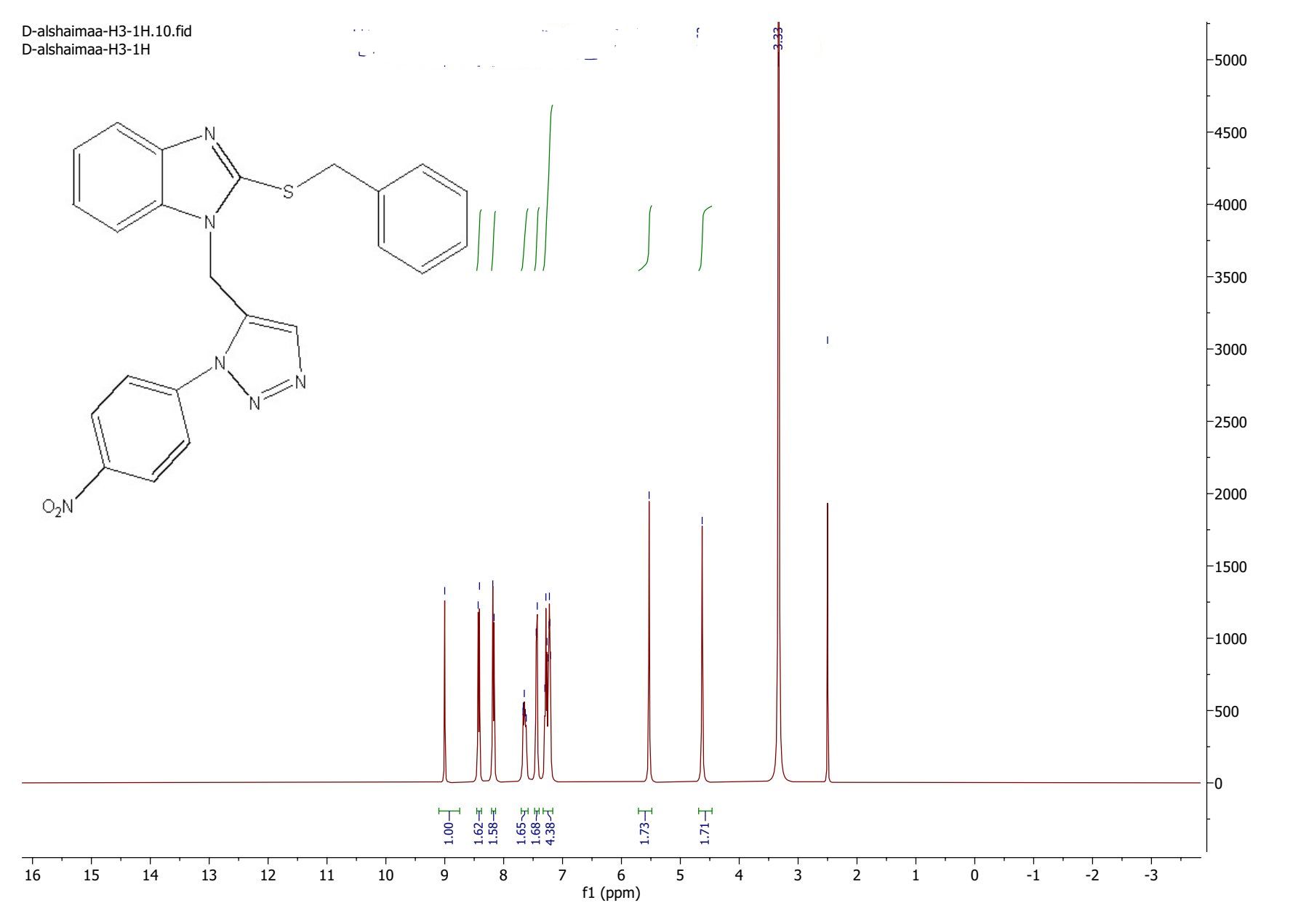
**

^1^H NMR (400 MHz, DMSO-*d*_6_): δ = 9 (s, 1H, triazole CH), 8.42 (d, *J* = 9.0 Hz, 2H, Ar-H-3 ̏,5 ̏ ), 8.17 (d, *J* = 8.9 Hz, 2H, Ar-H 2 ̏,6 ̏ ), 7.75 – 7.58 (m, 2H, Ar-H-4,7), 7.44 (d, *J* = 7.4 Hz, 2H, Ar-H) , 7.33 – 7.18 (m, 5H, Ar-H), 5.53 (s, 2H, NCH_2_(,4.63 (s, 2H, S-CH_2_)

**^13^C NMR spectrum of (10d):**

**
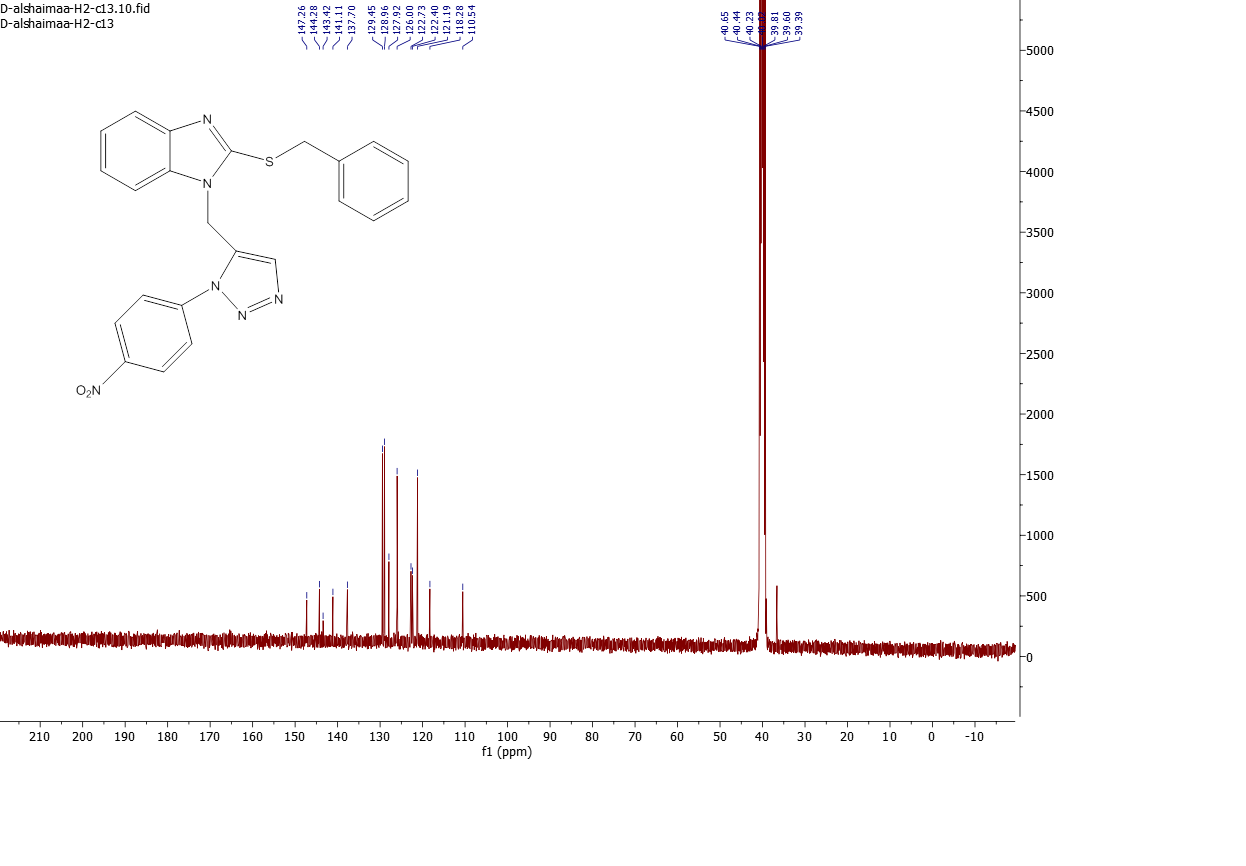
**

^13^C NMR (101 MHz, DMSO-*d*_6_) δ 147.2, 144.2, 143.4, 141.1, 137.7, 129.4, 128.9, 127.9, 126, 122.7, 122.4, 121.1, 118.2, 110.5, 39.1, 36.6

IR spectrum of (10d) :


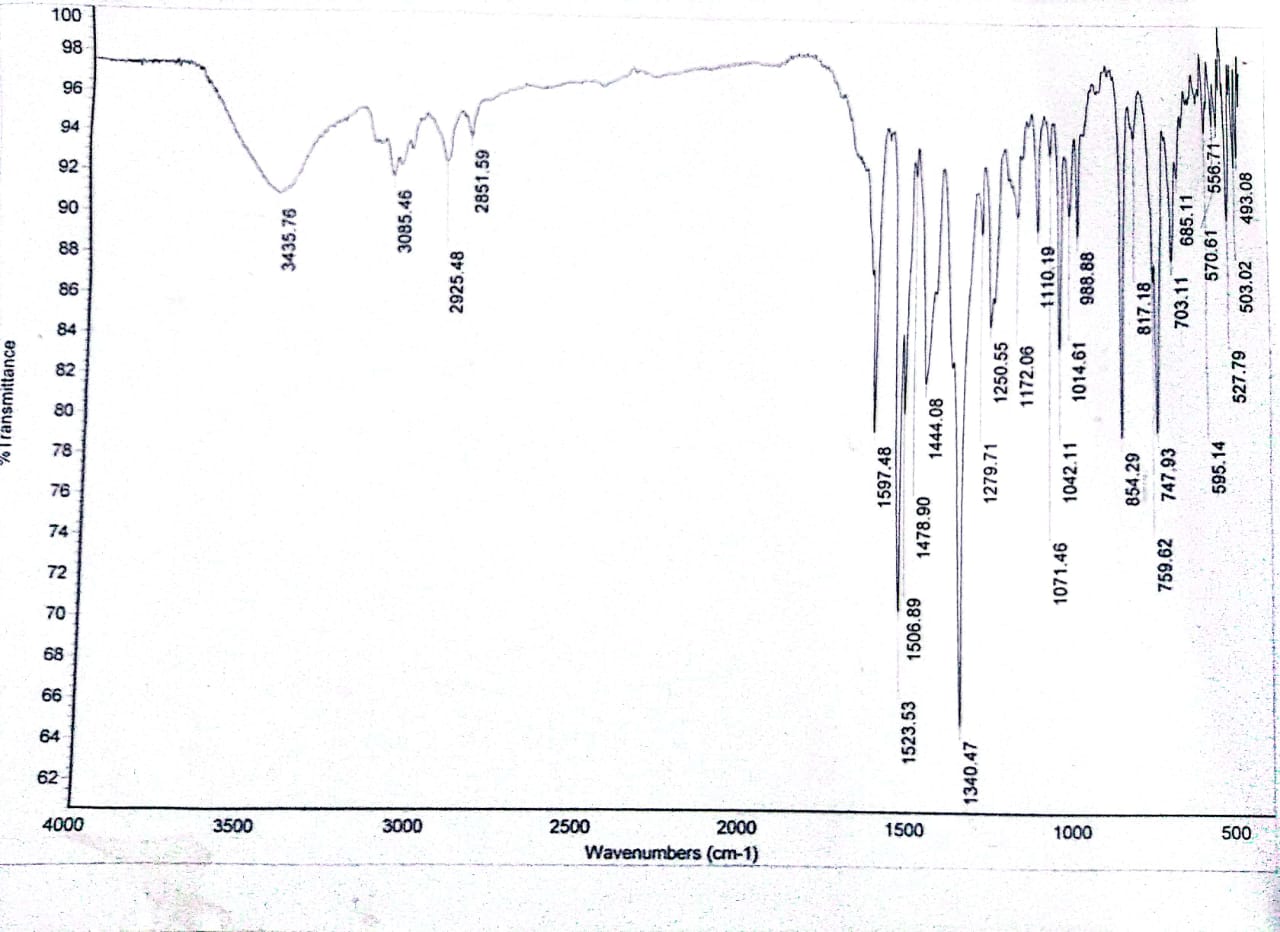


IR (KBr, ύ cm^-1^): 3085 (=CH), 2925 (CH_2_), 1597, 1507 (C=N, C=C), 1523, 1340 (NO_2_), 854 (*p*-bending)

**^1^H NMR spectrum of (10e):**

**
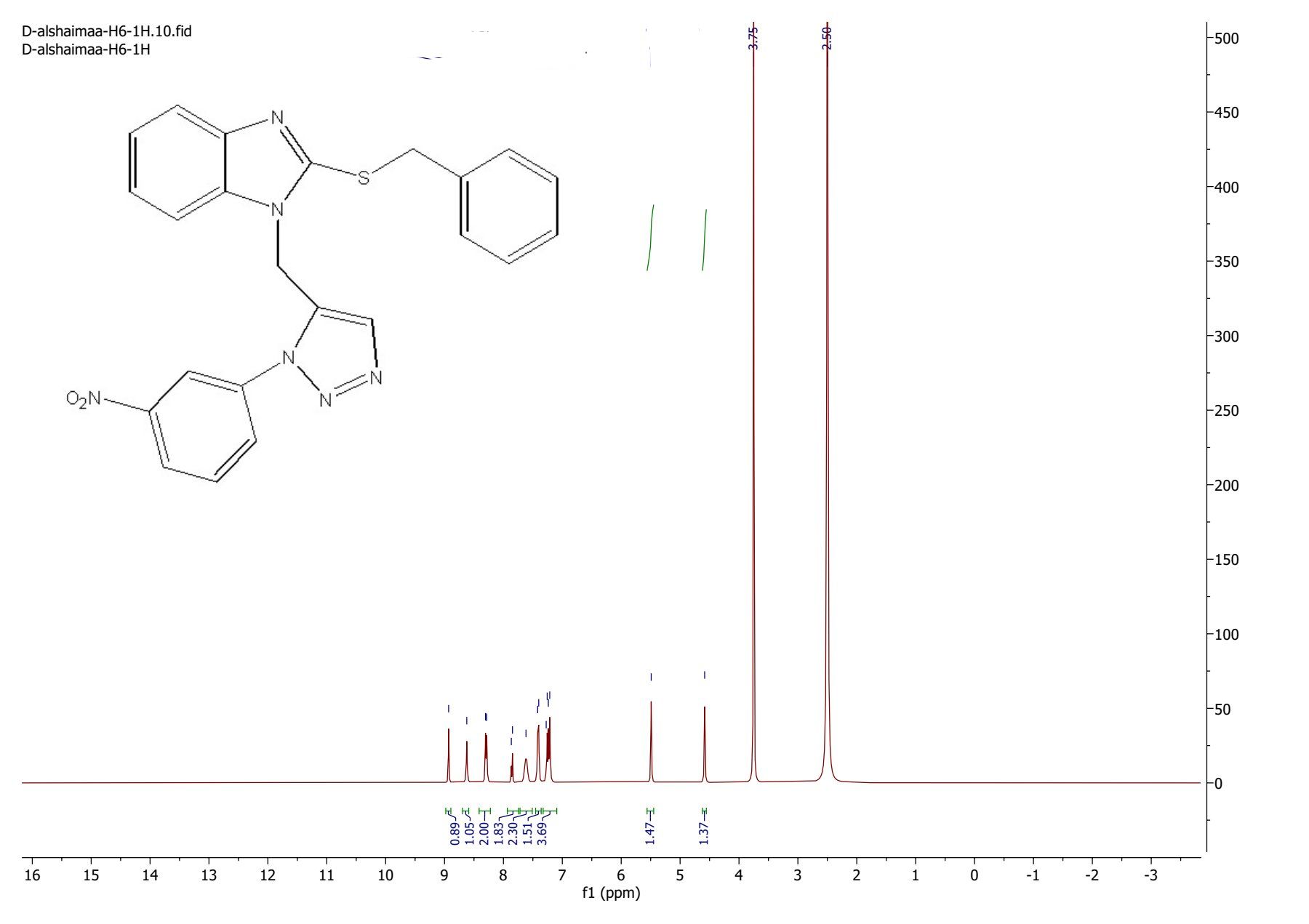
**

^1^H NMR (400 MHz, DMSO-*d_6_*): δ = 8.93 (s, 1H, triazole CH), 8.62 (d, *J* = 7.9 Hz, 1H, Ar-H) **,** 8.29 (d, *J* = 7.9 Hz, 2H, Ar-H), 7.86 (t, *J* = 7.9 Hz, 2H, Ar-H), 7.62 (bs, 2H, Ar-H), 7.41 (d, *J* = 7.4 Hz, 2H, Ar-H), 7.25 (m, 4H, Ar-H), 5.49 (s, 2H, NCH_2_( 4.58 (s, 2H,SCH_2_);

**^13^C NMR spectrum of (10e):**

**
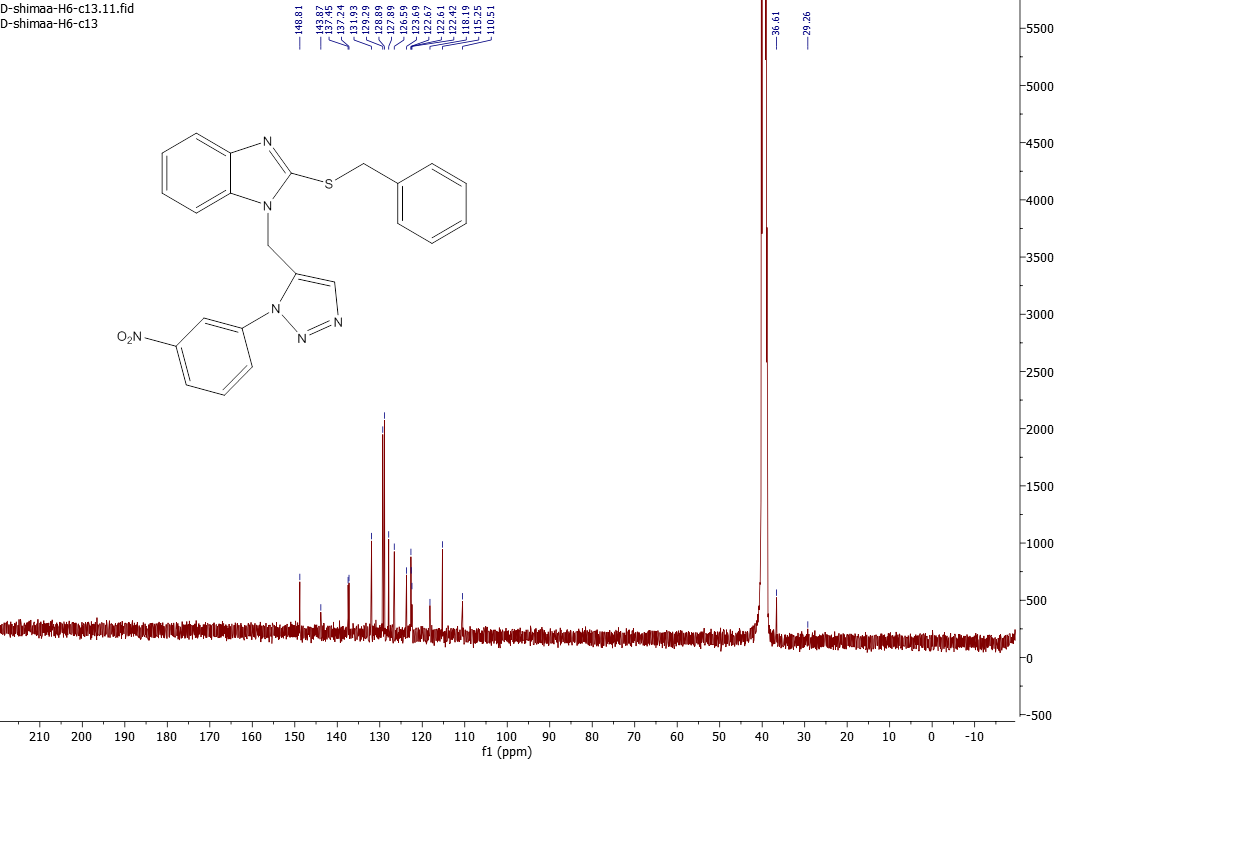
**

^13^C NMR (101 MHz, DMSO-*d*_6_) δ 148.8, 143.8, 137.4, 137.2, 131.9, 129.2, 128.8, 127.8, 126.5, 123.6, 122.6, 122.6, 122.4, 118.1, 115.2, 110.5, 36.6, 29.2

**^1^H NMR spectrum of (10f):**

**
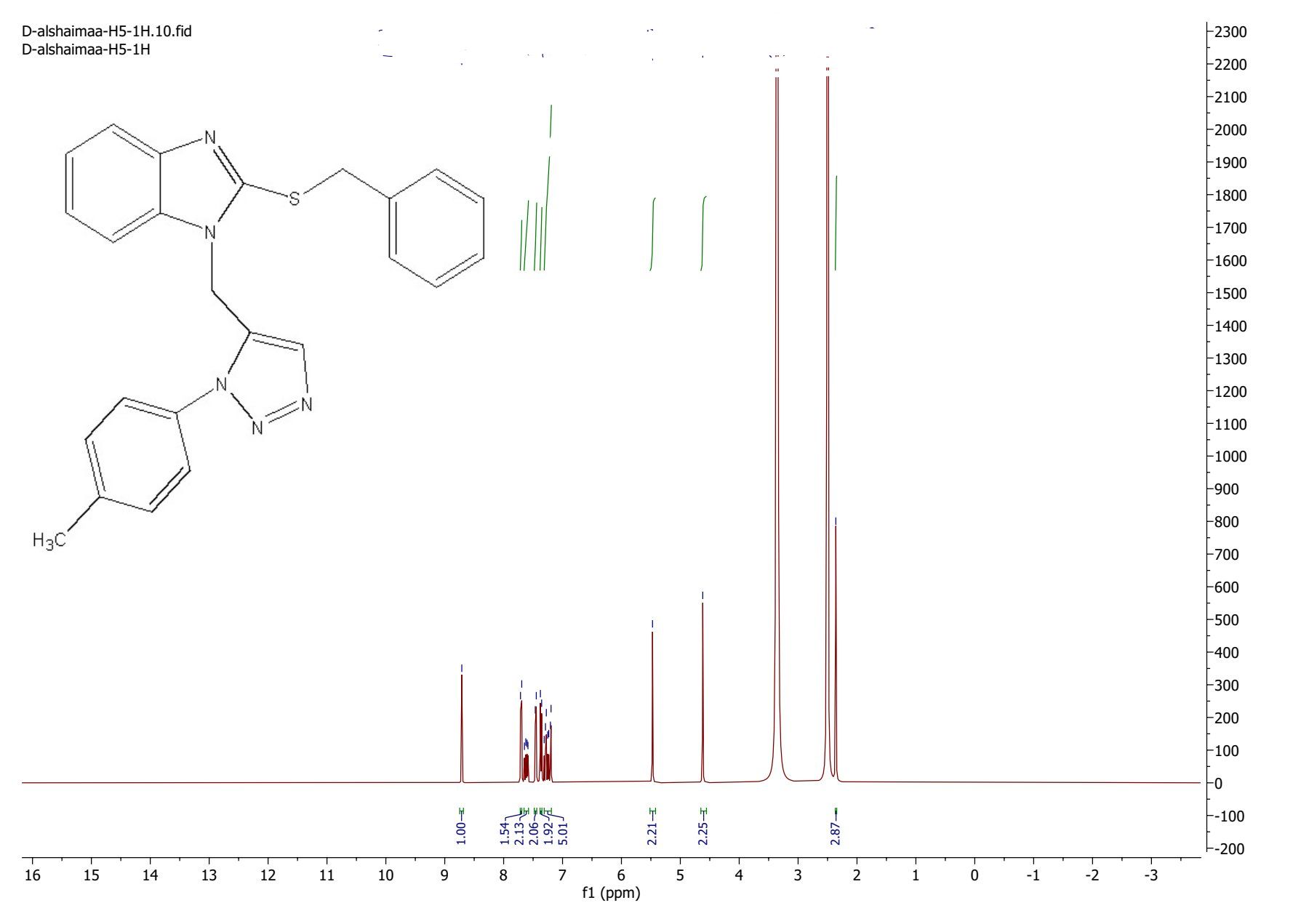
**

^1^H NMR (400 MHz, DMSO-*d_6_*): δ = 8.71 (s, 1H, triazole CH), 7.70 (d, *J* = 8.4 Hz, 2H, Ar-H), 7.67 – 7.56 (m, 2H, Ar-H), 7.45 (d, *J* = 6.8 Hz, 2H, Ar-H), 7.37 (d, *J* = 8.2 Hz, 2H, Ar-H), 7.31 – 7.19 (m, 5H, Ar-H), 5.47(s, 2H, NCH_2_(,4.62 (s, 2H, SCH_2_), 2.36 (s, 3H, CH_3_)

**^13^C NMR spectrum of (10f):**

**
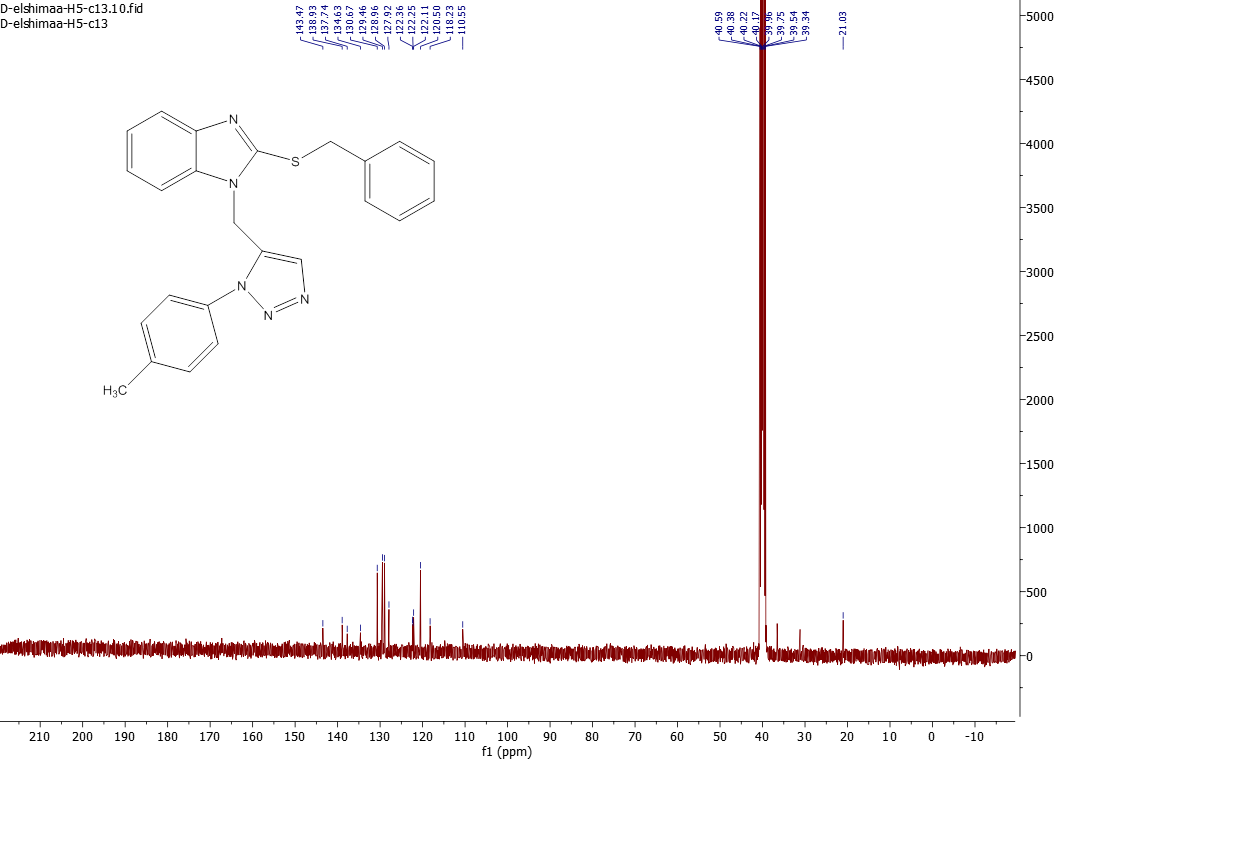
**

^13^C NMR (101 MHz, DMSO-*d_6_*) δ 143.4, 138.9, 137.7, 134.6, 130.6, 129.4, 128.9, 127.9, 122.3, 122.2, 122.1, 120.5, 118.2, 110.5, 36.5, 31.1, 21.0

**^1^H NMR spectrum of (10g):**

**
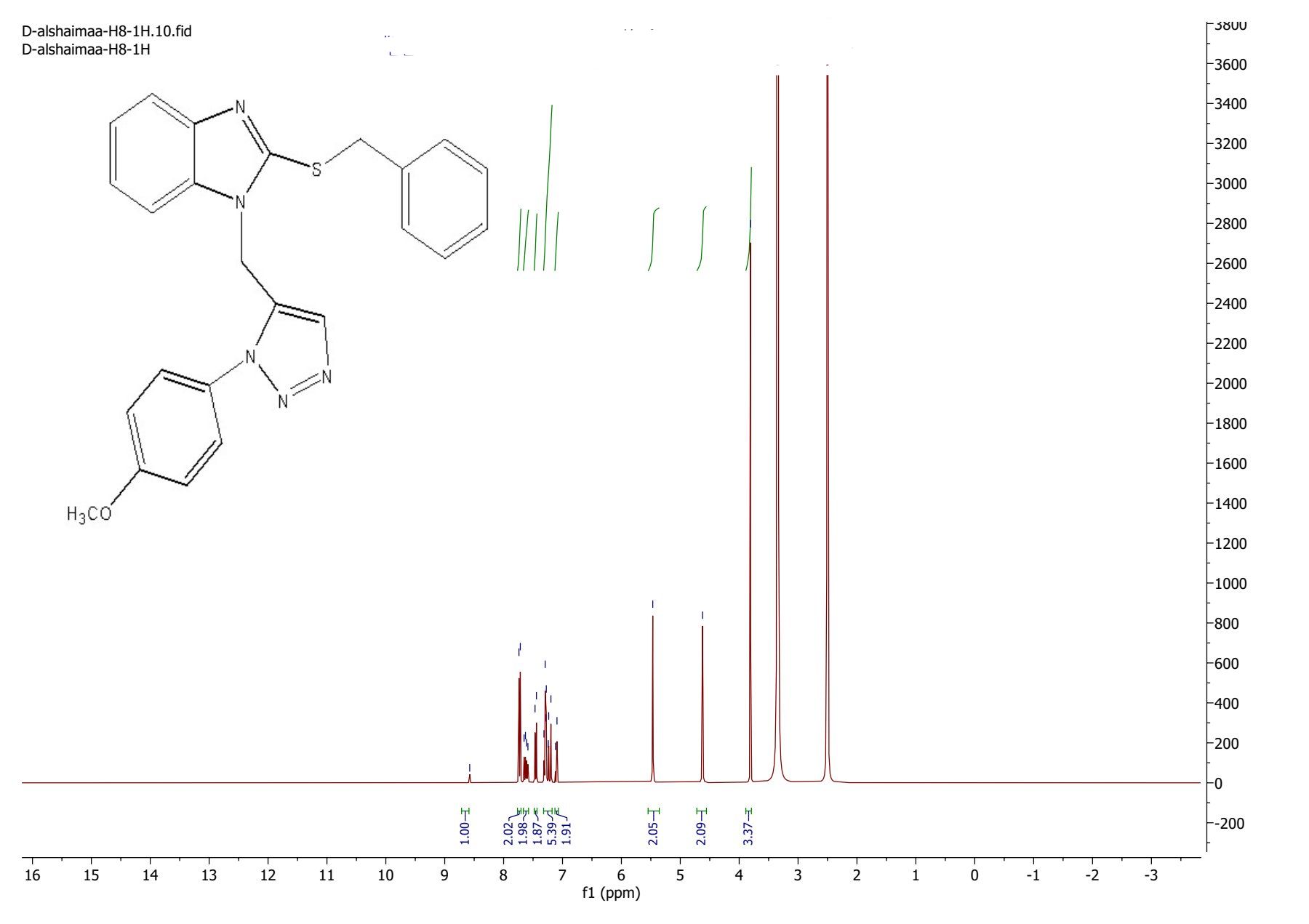
**

^1^H NMR (400 MHz, DMSO-d_6_): δ = 8.66 (s, 1H, triazole CH), 7.73 (d, *J* = 9.0 Hz, 2H, Ar-H), 7.65 – 7.56 (m, 2H, Ar-H-4,7), 7.49 – 7.42 (m, 2H, Ar-H), 7.34 – 7.15 (m, 5H, Ar-H), 7.10 (d, *J* = 9.0 Hz, 2H, Ar-H), 5.47 (s, 2H, NCH_2_(, 4.62 (s, 2H, SCH_2_), 3.81 (s, 3H, OCH_3_)

**^13^C NMR spectrum of (10g):**

**
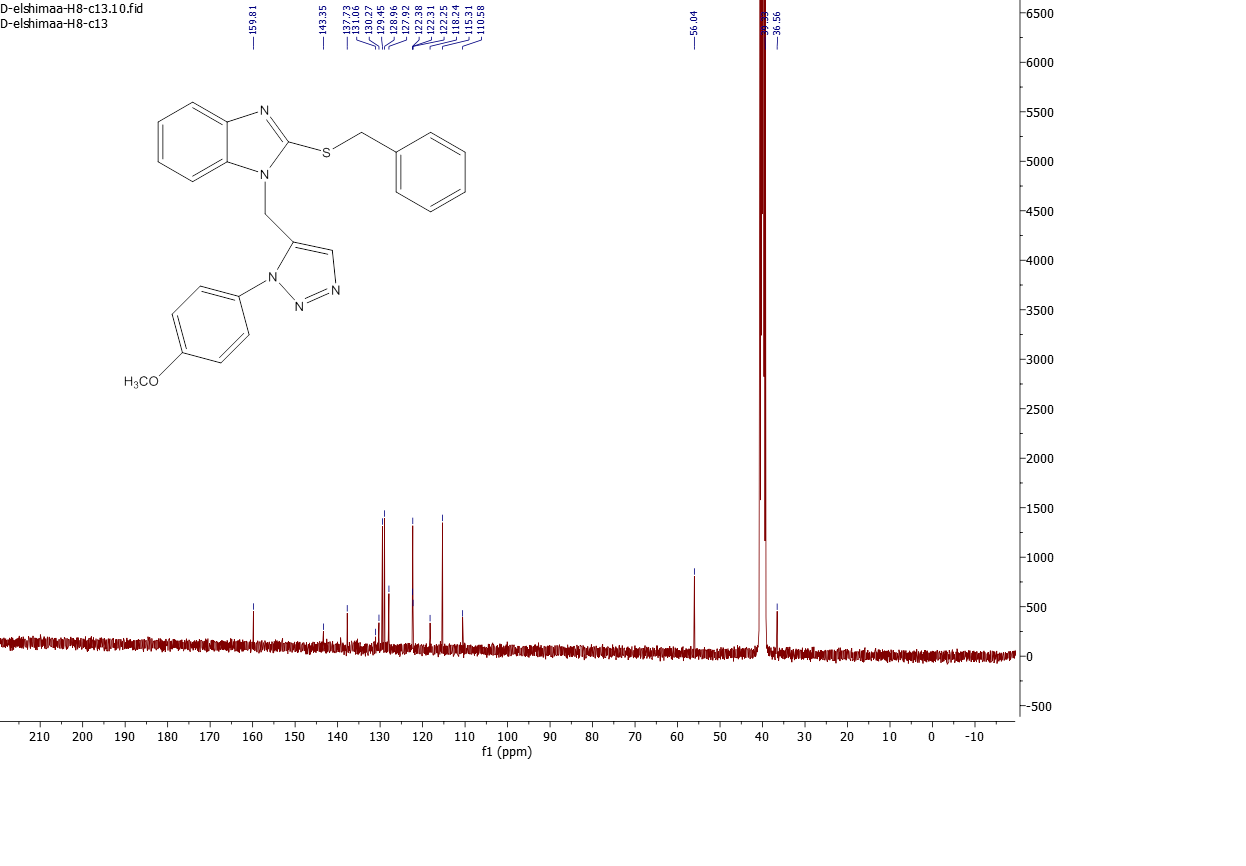
**

^13^C NMR (101 MHz, DMSO-*d_6_*) δ 159.8, 143.3, 137.7, 131, 130.2, 129.4, 128.9, 127.9, 122.3, 122.3, 122.2, 118.2, 115.3, 110.5, 56.0, 39.3, 36.5

**^1^H NMR spectrum of (10h):**

**
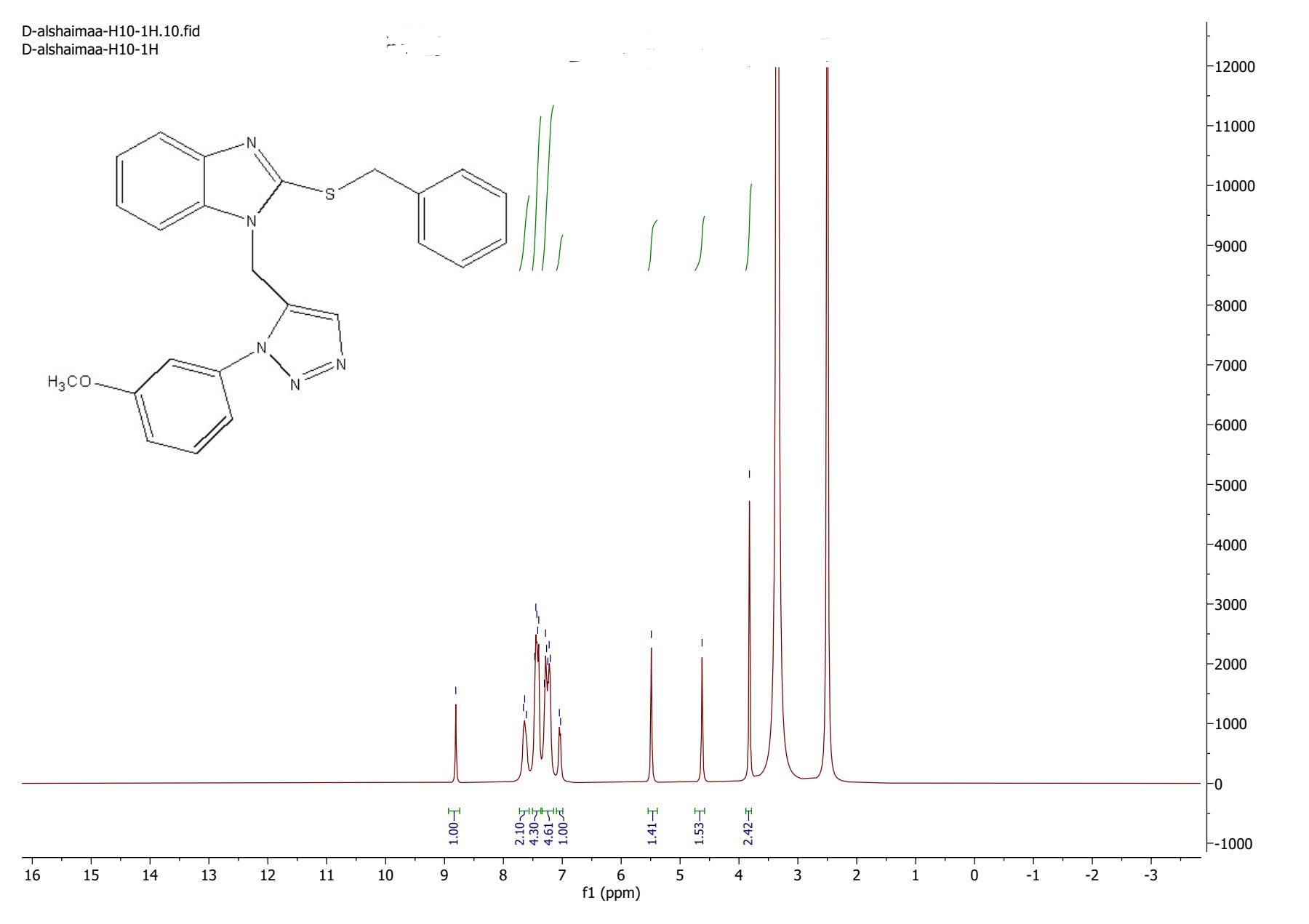
**

^1^H NMR (400 MHz, DMSO-d_6_): δ = 8.81 (s, 1H, triazole CH), 7.67 – 7.58 (m, 2H,Ar-H), 7.51 – 7.38 (m, 5H, Ar-H), 7.33 – 7.13 (m, 5H, Ar-H), 7.04 (d, *J* = 8.2 Hz, 1H, Ar-H), 5.49 (s, 2H, NCH_2_(, 4.63 (s, 2H, SCH_2_), 3.82 (s, 3H, OCH_3_).

**^13^C NMR spectrum of (10h):**

**
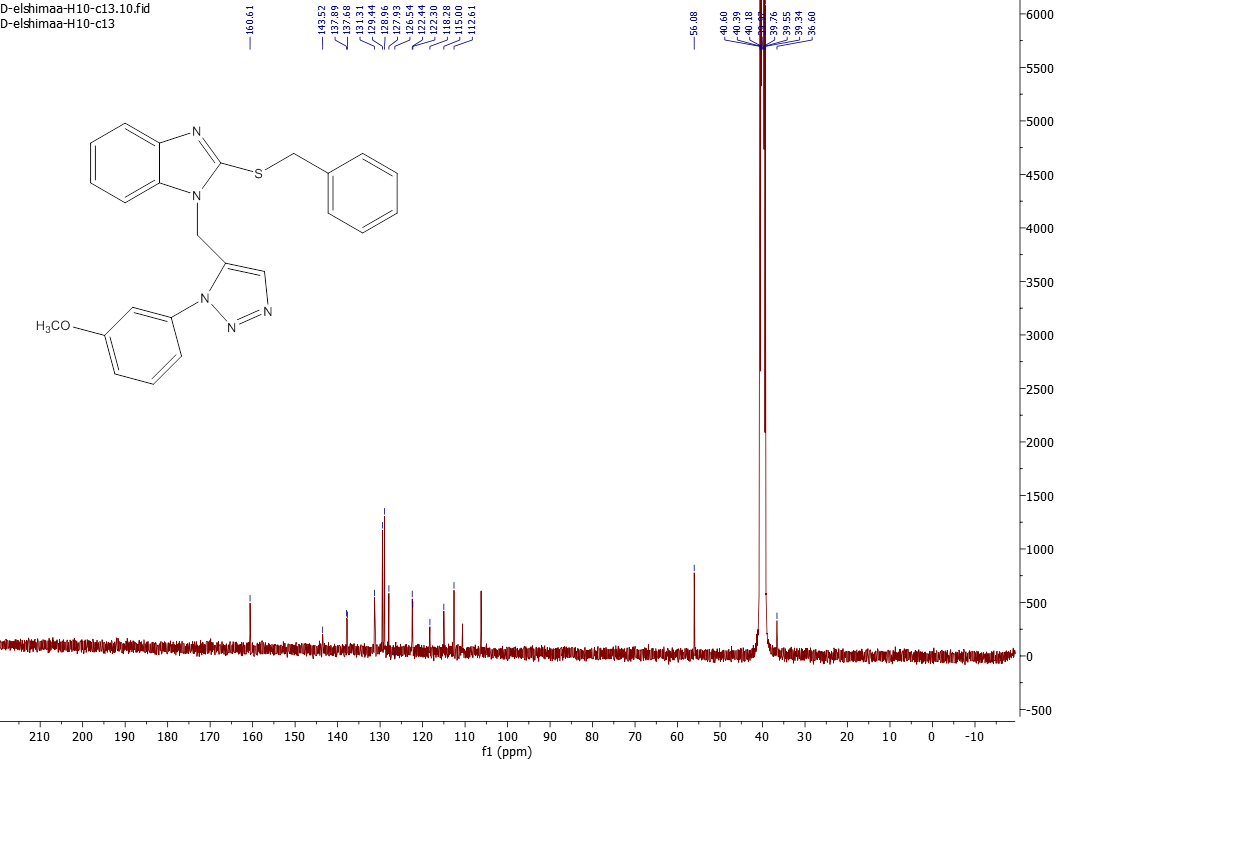
**

^13^C NMR (101 MHz, DMSO-*d_6_*) δ 160.6, 143.5, 137.8, 137.6, 131.3, 129.4, 128.9, 127.9, 126.5, 122.4, 122.3, 118.2, , 112.6, 110.6, 56.1, 39.3, 36.6

**^1^H NMR spectrum of (10i):**

**
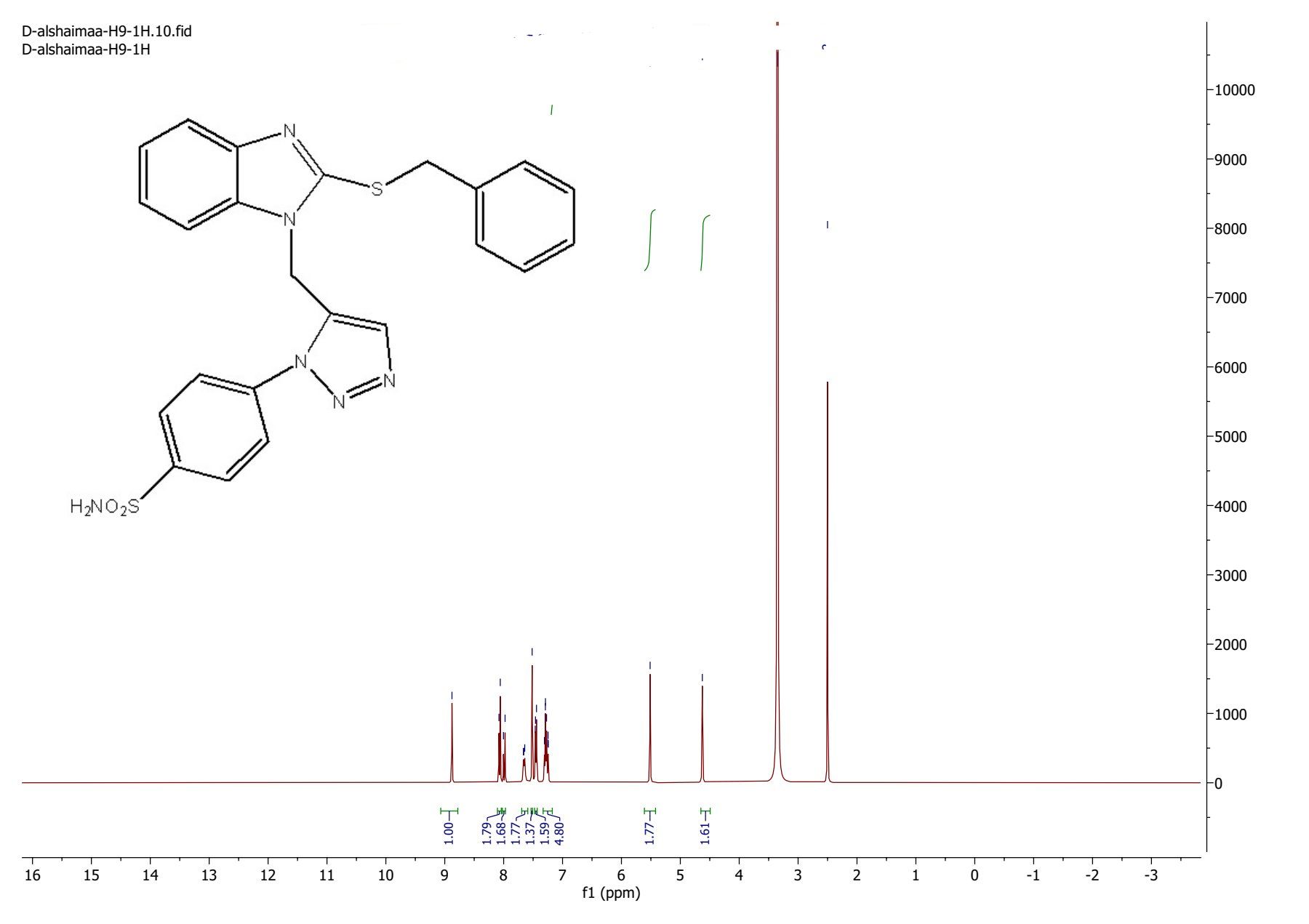
**

^1^H NMR (400 MHZ, DMSO-d_6_): δ= 8.88(s, 1H, triazole CH), 8.07 (d, *J* = 8.7 Hz, 2H, Ar-H-2 ̏, 6 ̏ ), 7.99 (d, 2H, Ar-H-3 ̏,5 ̏ ), 7.69 – 7.58 (m, 2H, Ar-H),7.52(S,2H, NH2), 7.45 (d, *J* = 6.9 Hz, 2H, Ar-H), 7.33 – 7.16 (m, 5H, Ar-H), 5.51(s,2H, NCH_2_(, 4.63(s,2H, S-CH_2_).

**^13^C NMR spectrum of (10i):**

**
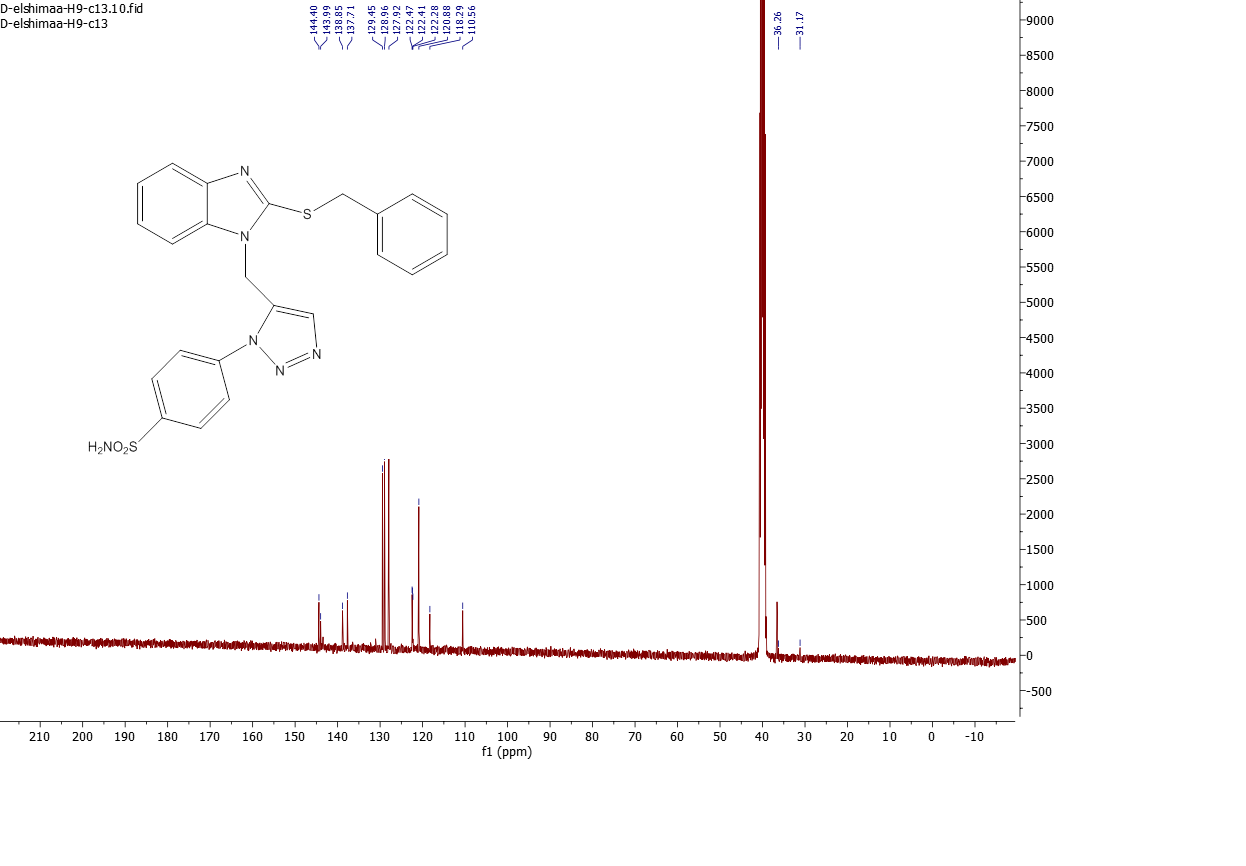
**

^13^C NMR (101 MHz, DMSO-*d*_6_) δ 144.4, 143.9, 138.8, 137.7, 129.4, 128.9, 127.9, 122.4, 122.4, 122.2, 120.8, 118.2, 110.5, 36.2, 31.1


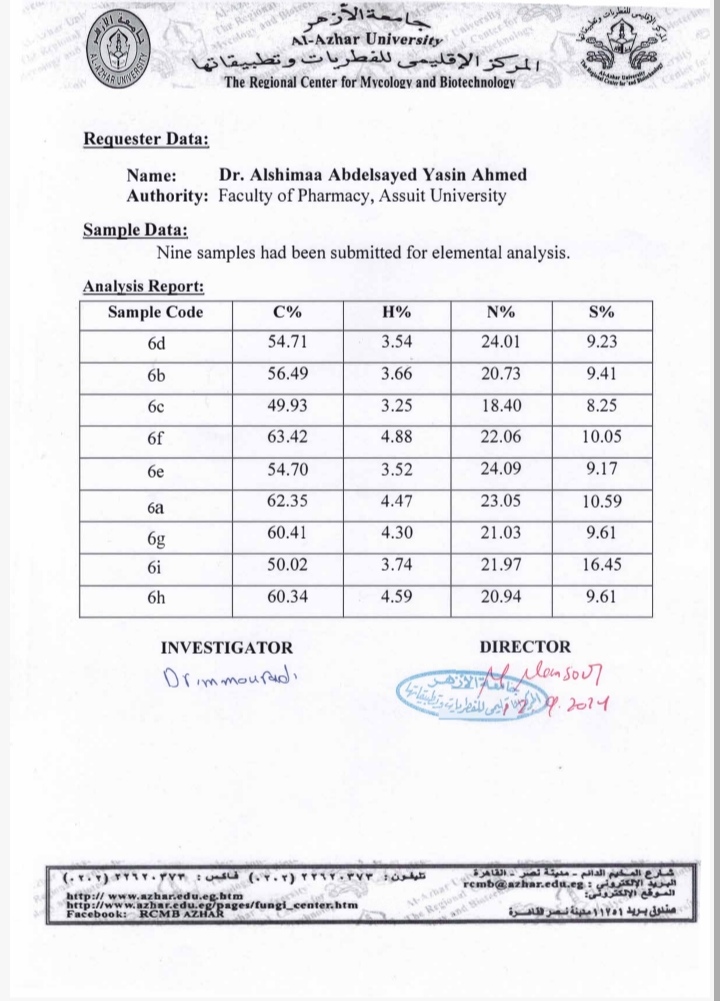


**
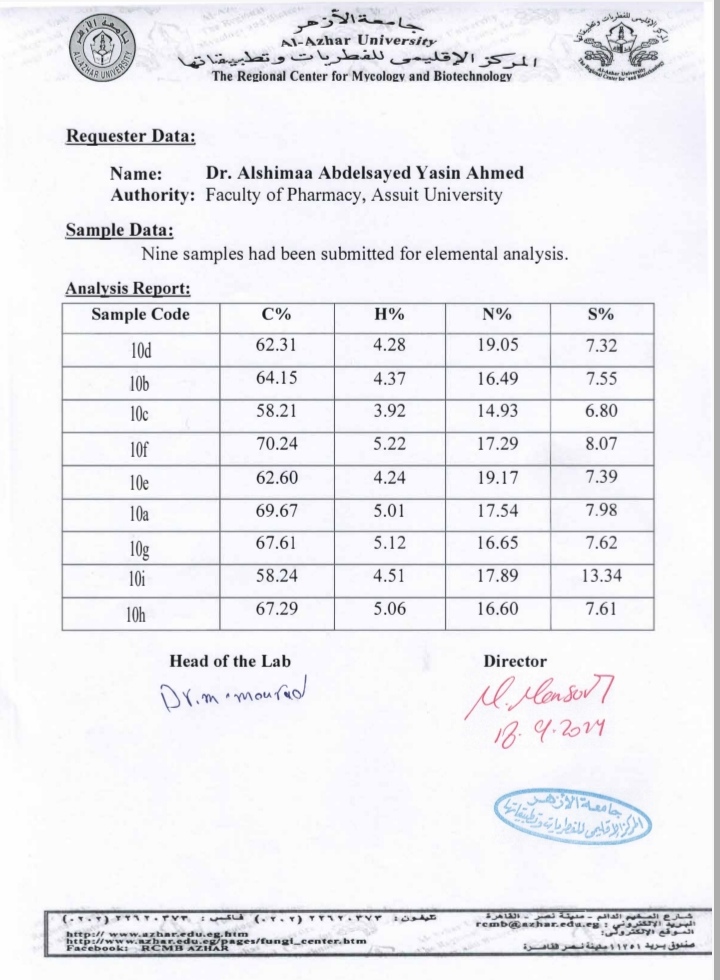
**

**Appendix A**

**EXPERIMENTAL**

**Chemistry**

**Materials and methods:** See Appendix A (Supplementary File)

Melting points were determined on Stuart scientific, model SMP3, UK and are uncorrected. Commercial-grade solvents and reagents were used to synthesize the specified compounds, The purification by column chromatography using silica gel and hexane: ethyl acetate as mobile phase. The progress of reactions was checked by TLC (pre-coated TLC sheets, 60G F254, Merk, Germany). UV light was used for detection at 254 nm wavelength (Spectroline, model CM-10, USA). The IR spectra were recorded on a thermoscientific Nicolet IS10 FTIR spectrometer (thermo Fischer scientific, USA) (KBr; ν max in cm^− 1^) at Faculty of Science, Assiut University, Assiut, Egypt. ^1^H NMR Spectra of some intermediates were recorded on a Varian EM- 360 L NMR spectrometer (60 MHz, Varian, CA, USA) at Faculty of Pharmacy, Assiut University, Assiut, Egypt. ^1^H and ^13^C NMR spectra of key intermediates and final compounds were scanned on Avance-III, High performance FT-NMR spectrum, Bruker biospin international AG Switzeraland at 400 MHz at Faculty of Science, Zagazig University, Egypt. Elemental microanalyses were performed on elemental analyzer model flash 2000 thermo fisher at the regional center for mycology and biotechnology (RCMB), faculty of science, Al-Azhar university, Nasr city, Cairo, Egypt.

**4.2. Biological evaluation**

**4.2.1. Cytotoxic activity using MTT Assay and evaluation of IC_50_**

**4.2.1.1. MTT assay**

MTT assay was carried out to study the effect of compounds on mammary epithelial cells (MCF-10A). The medium in which cells were propagated contained Dulbecco's modified Eagle's medium (DMEM)/ Ham's F-12 medium (1:1) supplemented with epidermal growth factor (20 ng/mL), hydrocortisone (500 ng/mL), insulin (10 μg/mL), 2 mM glutamine and 10% foetal calf serum. After every 2-3 days, the cells were passed using trypsin ethylenediamine tetra acetic acid (EDTA). The cells were seeded at a density of 10^4^ cells mL^-1^ in flat-bottomed culture plates containing 96 wells each. After 24 h, medium was removed from the plates and the compounds in (in 0.1% DMSO) were added (in 200 μL medium to yield a final concentration of 0.1% v/v) to the wells of plates. A single compound was designated with four wells followed by incubation of plates for 96h at 37°C. After incubation, medium was removed completely from the plates followed by addition of MTT (0.4 mg/mL in medium) to each well and subsequent incubation of plates for 3h. MTT (along with the medium) was removed and DMSO (150μL) was added to each well of the culture plates, followed by vortexing and subsequent measurement of absorbance (at 540 nm) using microplate reader. The data are shown as percentage inhibition of proliferation in comparison with controls containing 0.1% DMSO.

**4.2.1.2. Assay for antiproliferative effect**

To explore the antiproliferative potential of compounds MTT assay was performed according to previously reported procedure using different cell lines to explore the antiproliferative potential of compounds propidium iodide fluorescence assay was performed using different cell lines. To calculate the total nuclear DNA, a fluorescent dye (propidium iodide, PI) is used which can attach to the DNA, thus offering a quick and precise technique. PI cannot pass through the cell membrane and its signal intensity can be considered as directly proportional to quantity of cellular DNA. Cells whose cell membranes are damaged or have changed permeability are counted as dead ones. The assay was performed by seeding the cells of different cell lines at a density of 3000-7500 cells/well (in 200µl medium) in culture plates followed by incubation for 24h at 37 °C in humidified 5% CO_2_/95% air atmospheric conditions. The medium was removed; the compounds were added to the plates at 10 µM concentrations (in 0.1% DMSO) in triplicates, followed by incubation for 48 h. DMSO (0.1%) was used as control. After incubation, medium was removed followed by the addition of PI (25 µl, 50µg/mL in water/medium) to each well of the plates. At -80 °C, the plates were allowed to freeze for 24 h, followed by thawing at 25^o^C. A fluorometer (Polar-Star BMG Tech) was used to record the readings at excitation and emission wavelengths of 530 and 620 nm for each well. The percentage cytotoxicity of compounds was calculated using the following formula:


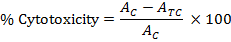


Where A*_TC_*= Absorbance of treated cells and A_C_= Absorbance of control. Erlotinib was used as positive control in the assay.

**4.2.1.3. EGFR inhibitory assay**

EGFR-TK assay was performed to evaluate the inhibitory potency of the most potent compounds against EGFR. Baculoviral expression vectors including pBlueBacHis2B and pFASTBacHTc were used separately to clone 1.6 kb cDNA coding for EGFR cytoplasmic domain (EGFR-CD, amino acids 645–1186). 5ʹ upstream to the EGFR sequence comprised a sequence that encoded (His)_6_. Sf-9 cells were infected for 72h for protein expression. The pellets of Sf-9 cells were solubilized in a buffer containing sodium vanadate (100 µM), aprotinin (10 µg/mL), triton (1%), HEPES buffer (50mM), ammonium molybdate (10 µM), benzamidine HCl (16 µg/mL), NaCl (10 mM), leupeptin (10 µg/mL) and pepstatin (10 µg/mL) at 0°C for 20 min at pH 7.4, followed by centrifugation for 20 min. To eliminate the non-specifically bound material, a Ni-NTA super flow packed column was used to pass through and wash the crude extract supernatant first with 10 mM and then with 100 mM imidazole. Histidine-linked proteins were first eluted with 250 and then with 500 mM imidazole subsequent to dialysis against NaCl (50 mM), HEPES (20 mM), glycerol (10%) and 1 µg/mL each of aprotinin, leupeptin and pepstatin for 120 min. The purification was performed either at 4 °C or on ice. To record autophosphorylation level, EGFR kinase assay was carried out on the basis of DELFIA/Time-Resolved Fluorometry. The compounds were first dissolved in DMSO absolute, subsequent to dilution to appropriate concentration using HEPES (25 mM) at pH 7.4. Each compound (10 µL) was incubated with recombinant enzyme (10 µL, 5 ng for EGFR, 1:80 dilution in 100 mM HEPES) for 10 min at 25^o^C, subsequent to the addition of 5X buffer (10 µL, containing 2 mM MnCl_2_, 100 µM Na_3_VO_4_, 20 mM HEPES and 1 mM DTT) and ATP-MgCl_2_ (20 µL, containing 0.1 mM ATP and 50 mM MgCl_2_) and incubation for 1h. The negative and positive controls were included in each plate by the incubation of enzyme either with or without ATP-MgCl_2_. The liquid was removed after incubation and the plates were washed thrice using wash buffer. Europium-tagged antiphosphotyrosine antibody (75 µL, 400 ng) was added to each well followed by incubation of 1h and then washing of the plates using buffer. The enhancement solution was added to each well and the signal was recorded at excitation and emission wavelengths of 340 at 615 nm. The autophosphorylation percentage inhibition by compounds was calculated using the following equation:


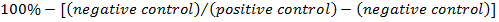


Using the curves of percentage inhibition of eight concentrations of each compound, IC_50_ was calculated. The majority of signals detected by antiphosphotyrosine antibody were from EGFR because the enzyme preparation contained low impurities.

**4.2.1.4. Caspase-3 activation assay**

Allow all reagents to reach room temperature before use. Gently mix all liquid reagents prior to use. Determine the number of 8-well strips needed for the assay. Insert these in the frame(s) for current use. Add 100 μl of the *Standard Diluent Buffer* to the zero standard wells. Well(s) reserved for chromogen blank should be left empty. Add 100 μl of standards and controls or diluted samples to the appropriate microtiter wells. The sample dilution chosen should be optimized for each experimental system. Tap gently on the side of plate to mix. Cover wells with *plate cover* and incubate for 2 hours at room temperature. Thoroughly aspirate or decant solution from wells and discard the liquid, Wash wells 4 times. Pipette 100 μl of *Caspase-3 (Active) Detection Antibod*y solution into each well except the chromogen blank(s). Tap gently on the side of the plate to mix. Cover plate with *plate cover* and incubate for 1 hour at room temperature. Thoroughly aspirate or decant solution from wells and discard the liquid, Wash wells 4 times. Add 100 μl Anti-Rabbit IgG HRP Working Solution to each well except the chromogen blank(s). Prepare the working dilution as described in Preparing IgG HRP. Cover wells with the *plate cover* and incubate for 30 minutes at room temperature. Thoroughly aspirate or decant solution from wells and discard the liquid. Wash wells 4 times. Add 100 μl of *Stabilized Chromogen* to each well. The liquid in the wells will begin to turn blue. Incubate for 30 minutes at room temperature and in the dark. The incubation time for chromogen substrate is often determined by the microtiter plate reader used. Many plate readers have the capacity to record a maximum optical density (O.D.) of 2.0. The O.D. values should be monitored, and the substrate reaction stopped before the O.D. of the positive wells exceeds the limits of the instrument. The O.D. values at 450 nm can only be read after the *Stop Solution* has been added to each well. If using a reader that records only to 2.0 O.D., stopping the assay after 20 to 25 minutes is suggested. Add 100 μl of *Stop Solution* to each well. Tap side of plate gently to mix. The solution in the wells should change from blue to yellow. Read the absorbance of each well at 450 nm having blanked the plate reader against a chromogen blank composed of 100 μl each of *Stabilized Chromogen* and *Stop Solution*. Read the plate within 2 hours after adding the *Stop Solution*. Use a curve fitting software to generate the standard curve. A four-parameter algorithm provides the best standard curve fit. Read the concentrations for unknown samples and controls from the standard curve. Multiply value(s) obtained for sample(s) by the appropriate dilution factor to correct for the dilution in step 3. Samples producing signals greater than that of the highest standard should be diluted in *Standard Diluent Buffer* and reanalyzed.

**4.2.1.5. Caspase-8 activation assay**

Cells were obtained from American Type Culture Collection, cells were grown in RPMI 1640 containing 10% fetal bovine serum at 37°C, stimulated with the compounds to be tested for caspase8, and lysed with Cell Extraction Buffer. This lysate was diluted in Standard Diluent Buffer over the range of the assay and measured for human active caspase-8 content. (*Cells are Plated in a density of 1.2 – 1.8 × 10,000 cells/well in a volume of 100µl complete growth medium + 100 ul of the tested compound per well in a 96-well plate for 24 hours before the enzyme assay for Tubulin*.). The absorbance of each microwell was read on a spectrophotometer at 450 nm. A standard curve is prepared from 7human Caspase-8 standard dilutions and human Caspase-8 concentration determined.

**4.2.1.6. Bax activation assay**

Bring all reagents, except the human Bax-α Standard, to room temperature for at least 30 minutes prior to opening. The human Bax-α Standard solution should not be left at room temperature for more than 10 minutes. All standards, controls and samples should be run in duplicate. Refer to the Assay Layout Sheet to determine the number of wells to be used and put any remaining wells with the desiccant back into the pouch and seal the ziploc. Store unused wells at 4 °C. Pipet 100 μL of Assay Buffer into the S0 (0 pg/mL standard) wells. Pipet 100 μL of Standards #1 through #6 into the appropriate wells. Pipet 100 μL of the Samples into the appropriate wells. Tap the plate gently to mix the contents. Seal the plate and incubate at room temperature on a plate shaker for 1 hour at ~500 rpm. Empty the contents of the wells and wash by adding 400 μL of wash solution to every well. Repeat the wash 4 more times for a total of 5 washes. After the final wash, empty or aspirate the wells and firmly tap the plate on a lint free paper towel to remove any remaining wash buffer. Pipet 100 μL of yellow Antibody into each well, except the Blank. Seal the plate and incubate at room temperature on a plate shaker for 1 hour at ~500 rpm. Empty the contents of the wells and wash by adding 400 μL of wash solution to every well. Repeat the wash 4 more times for a total of **5** washes. After the final wash, empty or aspirate the wells and firmly tap the plate on a lint free paper towel to remove any remaining wash buffer. Add 100 μL of blue Conjugate to each well, except the Blank. Seal the plate and incubate at room temperature on a plate shaker for 30 minutes at ~500 rpm. Empty the contents of the wells and wash by adding 400 μL of wash solution to every well. Repeat the wash 4 more times for a total of **5 washes**. After the final wash, empty or aspirate the wells and firmly tap the plate on a lint free paper towel to remove any remaining wash buffer. Pipet 100 μL of Substrate Solution into each well. Incubate for 30 minutes at room temperature on a plate shaker at ~500 rpm. Pipet 100 μL Stop Solution to each well. Blank the plate reader against the Blank wells, read the optical density at 450 nm. Calculate the average net Optical Density (OD) bound for each standard and sample by subtracting the average Blank OD from the average OD for each standard and sample. Using linear graph paper, plot the Average Net OD for each standard versus Bax concentration in each standard. Approximate a straight line through the points. The concentration of Bax in the unknowns can be determined by interpolation.

**4.2.1.7. Bcl-2inhibition assay**

Mix all the reagents thoroughly without foaming before use. Wash the microwells twice with approximately 300 μL Wash Buffer per well with thorough aspiration of microwell contents between washes. Take caution not to scratch the surface of the microwells. After the last wash, empty the wells and tap microwell strips on an absorbent pad or paper towel to remove excess Wash Buffer. Use the microwell strips immediately after washing or place upside down on a wet absorbent paper for not longer than 15 minutes. Do not allow wells to dry. Add 100 μL of Sample Diluent in duplicate to all standard wells and to the blank wells. Prepare standard (1:2 dilution) in duplicate ranging from 32 ng/mL to 0.5 ng/mL. Add 100 μL of Sample Diluent, in duplicate, to the blank wells. Add 80 μL of Sample Diluent, in duplicate, to the sample wells. Add 20 μL of each Sample, in duplicate, to the designated wells. Add 50 μL of diluted biotin-conjugate to all wells, including the blank wells. Cover with a plate cover and incubate at room temperature, on a microplate shaker at 100 rpm if available, for 2 hours. Remove plate cover and empty the wells. Wash microwell strips 3 times as described in step 2. Add 100 μL of diluted Streptavidin-HRP to all wells, including the blank wells. Cover with a plate cover and incubate at room temperature, on a microplate shaker at 100 rpm if available, for 1 hour. Remove the plate cover and empty the wells. Wash microwell strips 3 times as described in step 2. Proceed to the next step. Pipette 100 μl of mixed TMB Substrate Solution to all wells, including the blanks. Incubate the microwell strips at room temperature (18° to 25°C) for about 15 minutes, if available on a rotator set at 100 rpm. Avoid direct exposure to intense light. The point, at which the substrate reaction is stopped, is often determined by the ELISA reader. Many ELISA readers record absorbance only up to 2.0 O.D. Therefore, the color development within individual microwells must be watched by the person running the assay and the substrate reaction stopped before positive wells are no longer properly detectable. Stop the enzyme reaction by quickly pipetting 100 μL of Stop Solution into each well, including the blank wells. It is important that the Stop Solution is spread quickly and uniformly throughout the microwells to completely inactivate the enzyme. Results must be read immediately after the Stop Solution is added or within one hour if the microwell strips are stored at 2 - 8°C in the dark. Read the absorbance of each microwell on a spectrophotometer using 450 nm as the primary wavelength.

**Molecular Modelling**

All the molecular modeling calculations and docking simulation studies were performed on a Processor Intel(R) Pentium(R) CPU N3510@ 1.99GHz and 4 GB Memory with Microsoft Windows 8.1 pro (64 Bit) operating system using Molecular Operating Environment (MOE 2019.0102, 2020; Chemical Computing Group, Canada) as the computational software. All MOE minimizations were performed until a RMSD gradient of 0.01 Kcal/mol/Å with the force field (OPLS-AA) to calculate the partial charges automatically using Born solvation. Before simulations, the protein was solvated and protonated using QuickPrep function. Triangle matching with London dG scoring was chosen for initial placement, and then the top 30 poses were refined using force field (OPLS-AA) and GBVI/WSA dG scoring. The output database dock file was created with different poses for each ligand and arranged according to the final score function (S), which is the score of the last stage that was not set to zero.

**4.4. Statistical analysis**

Computerized Prism 5 program was used to statistically analyzed data using one-way ANOVA test followed by Tukey’s as post ANOVA for multiple comparison at P ≤.05. Data were presented as mean ± SEM.
